# Can transformative experiences bridge the gap between receiving communities and formerly incarcerated persons?

**DOI:** 10.1111/bjso.12886

**Published:** 2025-05-06

**Authors:** Linus Peitz, Harvey Whitehouse, Martha Newson

**Affiliations:** ^1^ University of Greenwich London UK; ^2^ University of Kent Canterbury UK; ^3^ University of Oxford Oxford UK

**Keywords:** formerly incarcerated persons, identity fusion, perceived chances, reintegration, transformative experiences, willingness to hire

## Abstract

The stigma of incarceration contributes to the global reoffending crisis and remains a barrier to reintegration into receiving communities. Recent research suggests that the key to solving this problem may lie in shared transformative experiences. We tested whether the salience of such experiences can overcome stigma among members of receiving communities when they act as gatekeepers for formerly incarcerated persons seeking employment. Across four experimental studies with seven samples of US and UK nationals (*N* = 2091), we examined the conditions under which transformative experiences can lead to identity fusion, a powerful form of social bonding and contribute to hiring and optimism about reintegration among prospective employers. In six of seven samples, those who reported stronger transformative experiences of their own were more fused to a job applicant, which was linked to positive attitudes towards them and willingness to hire them. Effects of formerly incarcerated persons' experiences varied between national samples and experience contexts: American citizens were more receptive to experiences in prison, while British citizens were more influenced by sports experiences. These findings highlight the potency of transformative experiences to forge connective bridges to stigmatized groups, despite cultural differences in perceptions of relevant social cues about formerly incarcerated people.

## INTRODUCTION

Inclusive societies are key to human flourishing by maximizing the engagement of available talent and encouraging participation in the workforce and in political processes (Acemoglu & Robinson, 2012). However, around 1.8 million people in the United States and around 95,000 people in the United Kingdom are excluded from this by virtue of being imprisoned and, even after they are released, most will be barred from participating fully in many aspects of social, economic and political life owing to the stigma associated with having a criminal record (Newton et al., 2019; Williams & Treffers, 2021). Such stigma manifests in persistent public opinion favouring harsher sentencing and resisting criminal justice reform (Pickett, 2019; Wood, 2011; Wood & Viki, 2001) but also in barriers to everyday reintegration efforts, for example, through the provision of employment opportunities (Varghese et al., 2021).

It has previously been proposed that social connections between members of receiving communities and formerly incarcerated persons can be elicited by highlighting shared transformative life experiences that serve as meaningful cues of a social bond (Whitehouse & Fitzgerald, 2020), including in an employment‐seeking context (Reich et al., 2024). Here, we build on this approach by examining the conditions under which transformative life experiences can elicit identity fusion, a particularly strong type of social bonding associated with pro‐social behaviour (Swann Jr. et al., 2012), and contribute to reintegration support in an employment‐seeking context. For the first time, we examine the interplay of gatekeepers' own transformative experiences and the *perceived* transformative experiences of formerly incarcerated persons. We focus on receiving communities in the United States and the United Kingdom, two countries with similar justice systems and particularly large prison populations (Fair & Walmsley, 2021).

### The potential role of shared experiences in tackling social stigma

Socio‐economic stability is consistently linked to reintegration success (Harding et al., 2017), and it is well established that employment (or the lack thereof) is an important dynamic (i.e. modifiable) risk factor for reoffending (Sampson & Laub, 1993; for a recent meta‐analysis, see Basto‐Pereira & Farrington, 2022). Consequently, many programmes and policies seek to facilitate economic reintegration by targeting vocational and educational needs among (formerly) incarcerated persons. However, research has produced mixed evidence as to their effectiveness, both concerning increased employability (Connell et al., 2023) and reduced reoffending rates (Newton et al., 2018; Visher et al., 2005; Zweig et al., 2011).

In addition to the specific legal hurdles formerly incarcerated persons may face when re‐entering the labour market (e.g. restrictions on freedom of movement, Pager, 2003), almost all are obliged to disclose their criminal records (Thomas & Hebenton, 2013). Studies conducted in the United States (Moses, 2014) and the United Kingdom (Sodexo, 2023; Working Chance, 2022) have shown that while most employers are open to hiring people with criminal records, this is often tied to various conditions. Moreover, many employers hold negative attitudes towards people who have served time in prison in contrast to those who have not (Moses, 2014; Working Links, 2010), which may prevent them from hiring.

Among the factors that have been linked to lay attitudes towards formerly incarcerated persons are personal characteristics, including differences based on demographics (Guy & Edens, 2006; Willis et al., 2013), belief systems (Tajalli et al., 2013) and exposure to people involved in the justice system (Hirschfield & Piquero, 2010; Lageson et al., 2019), as well as a person's criminal history (Clark, 2007). In a recent meta‐analysis, Rade et al. (2016) found that criminal records, including sexual crimes and higher levels of right‐wing ideology, predicted more negative attitudes towards incarcerated persons, whereas more frequent contact was associated with more favourable attitudes. The latter, being a modifiable factor, has been suggested as a pathway to improve public attitudes towards this group, drawing on the mechanisms underlying interpersonal contact, that is, increase perceived similarities and decrease perceived differences (Pettigrew & Tropp, 2008).

The recognition of shared characteristics, including physical appearance or demographics, personal values or histories, but also shared knowledge and experiences, reflects an integral part of organizing oneself and others into meaningful social groups and categories (Turner et al., 1987), which then influences how people engage with, and potentially support others as members of social in‐ or outgroups (Tajfel & Turner, 1986). While access to, and identification with social groups, particularly those associated with desirable characteristics, has been linked to psychological wellbeing and health (the social cure model; Jetten et al., 2012; Peitz & Newson, 2024), stigmatized group identities, particularly in the context of criminal justice, have been linked to poorer health and reintegration struggles (Kellezi et al., 2019; Kyprianides et al., 2019). In contexts where group identities are resisted due to stigma, or where shared group identities are not clearly established, two scenarios highly relevant when considering interactions between formerly incarcerated persons and receiving communities, shared experiences can be particularly important as they allow individuals to connect without the need for an explicitly shared identity (Drury, 2012). For example, Bradshaw and Muldoon (2020) found that family members of incarcerated persons were able to bond with and draw on resources from a support group by connecting over distinct shared experiences.

The communication of shared characteristics and experiences is also key when formerly incarcerated individuals re‐enter the labour market. According to Schriro (2012), stigmatization in this context can be overcome via the signalling of relevant group normative characteristics and qualities that are incongruent with stereotypical criminal behaviour. Reich (2017) showed that the perception of both desirable hard and soft skills in an applicant with a criminal record significantly contributed to employers' willingness to hire. Snider and Reysen (2014) found that people were more likely to hire formerly incarcerated persons when they perceived them as more likeable and more similar (e.g. law‐abiding) to themselves. Reich (2023) further stipulated that such signals are more potent when they come directly from the applicant rather than a third‐party source. The key is for receiving communities to perceive the capacity of those involved in the justice system to break criminal behaviour patterns to signal personal change. With this in mind, Reich et al. (2024) tested whether the disclosure of particularly important (life‐changing) experiences could function as a highly authentic signal which, if the meaning of the experience is shared between both applicant and employer, would lead to higher perceived similarity and social bonding. The implication of this could be substantial for marginalized or stigmatized groups, which we sought to examine here.

Reich et al.'s approach utilizes theoretical work in anthropology (Whitehouse, 2021) and empirical findings in social psychology (Swann Jr. et al., 2012) on the effects of identity fusion: an intense form of social bonding associated with strong forms of prosocial action. Identity fusion involves a synergistic relationship between personal and group identities: the personal self feels strengthened by the group and the any attack on the group is taken personally (Swann & Buhrmester, 2015). This fusion of personal and social identities is thought to occur via reflection on shared, personally transformative experiences and/or perceptions of shared biology (Vázquez et al., 2017; Whitehouse & Lanman, 2014) – both pathways involved in the sharing of personal essence with other group members. Studies show that people can become fused with extended in‐groups (Jong et al., 2015; Kavanagh et al., 2020), and, importantly, even outgroups such as rival ethnic or political groups or rival football fans (Buhrmester et al., 2022; Kunst et al., 2018; Newson et al., 2023a; Newson et al., 2023b).

Here, we focus on transformative experiences, which can, at times, produce even stronger ties than those based on kinship (Whitehouse et al., 2014). This is perhaps the most obvious pathway for receiving communities to fuse with and provide support for formerly incarcerated persons (Whitehouse & Fitzgerald, 2020). Experiences capable of eliciting fusion can be found in various contexts. Some experiences are implicitly shared by large parts of the general population, such as childbirth (Tasuji et al., 2020) or bereavement (Vázquez et al., 2019), while others are relevant for members of specific social groups, including experiences of national tragedies (Buhrmester et al., 2015; Jong et al., 2015) or even particularly painful sporting defeats (Newson et al., 2023a; Newson et al., 2023b). Dysphoric experiences are considered particularly potent at eliciting fusion because they tap into evolutionary adaptive responses to group threat (Whitehouse et al., 2017), although there is also evidence that euphoric experiences of transformation (e.g. following the consumption of psycho‐active drugs or winning sports event) can lead to fusion (Newson et al., 2016, 2021).

Testing the theorized role of dysphoric transformative experiences in an employment‐seeking context, Reich et al. (2024) conducted a study where participants imagined themselves as prospective employers interacting with a job applicant with a criminal record. In the experimental conditions, the applicant stated his intentions to desist from crime and that he felt transformed, either revealing a direct bereavement episode (having lost his mother to cancer) or an indirect bereavement episode (having witnessed his cellmate lose their mother to cancer). Reich and colleagues found that participants in the direct bereavement condition were more likely to hire the applicant and that the association between transformative experience disclosure and willingness to hire was explained (i.e. mediated) by the levels of identity fusion participants felt towards the applicant. Reich et al. further tested whether the experimental effects on fusion and willingness to help were dependent on (i.e. moderated by) the extent to which participants rated their own experiences of bereavement as transformative (either with reference to a general personal loss or specifically referring to cancer‐related loss). They found that those who rated personal bereavement as more transformative showed higher levels of fusion and support, but independently of what the applicant disclosed (i.e. the experimental condition). Interestingly, both ratings of general and cancer‐specific bereavement experiences were significant predictors, indicating that shared experiences capable of connecting receiving communities and formerly incarcerated people must not necessarily be identical.

### The present research

In this research, we expand on Reich et al.'s work and test whether an awareness of transformative experiences can elicit fusion with members of a stigmatized group across a variety of contexts. Specifically, we examined whether participants in the role of a prospective employer would become more fused to, and willing to hire, an applicant with a criminal record when they were exposed to a cue that the applicant has had a transformative experience. We presented cues of transformative experiences in different contexts which had previously been shown to elicit fusion, focusing on dysphoric events, including one that is implicitly shared among all human beings (S1, bereavement), and some that are relevant among members of particular social groups (S2, national tragedies for citizens, S3 bitter defeats for sports fans), as well as one that is uniquely relevant to formerly incarcerated persons and is generally not shared by the receiving communities (S4, imprisonment).

We also examined the extent to which such effects require participants to recognize the experience context as transformative. Initially (S1–3), we asked participants to think about the type of experience that the applicant disclosed in the experimental conditions (S1, having lost someone; S2, having experienced a natural disaster; S3, having suffered a painful sports defeat), presuming these are familiar episodes and that people who consider them more personally transformative will be more impacted by the disclosure of a similar experience. Eventually (S4), we also considered whether awareness of a personal experience that is not linked to the disclosed experience of the applicant can be linked with fusion and support, as long as participants rates both experiences as highly transformative; in this way the shared experience resides in the nature of feeling personally transformed, rather than in the nature of the event (Paul, 2014).

We tested the following main hypotheses

Potential employers will report greater identity fusion to job applicants who appear to have experienced personally transformative events (S1‐4).

Identity fusion predicts willingness to hire the applicant (S1‐4) and perceived chances to desist from crime (S2‐4).

Identity fusion acts as a mediator between transformative experiences and willingness to hire/ perceived chances to desist from crime.

The effect of transformative experiences on identity fusion (H1) is dependent on (i.e. moderated by) the extent to which the potential employers feel personally transformed themselves.

We tested our hypotheses across four studies, with seven samples of US and UK nationals. The United States has the largest population of incarcerated persons in the world, and the United Kingdom has the highest number of inmates in European countries (Fair & Walmsley, 2021). Thus, the research has the potential to advance our understanding of reintegration mechanisms for populations that are particularly impacted by failure to reintegrate formerly incarcerated persons, and where community members are more likely to assume the role of a gatekeeper at some point in their professional career. All studies were conducted as part of a larger body of research into the relevance of social bonds between reintegrating and receiving communities. Studies 1a, 3a and 4a were pre‐registered (https://osf.io/x8dz2; https://osf.io/nt2e4, https://osf.io/gpfrs), and studies 1b, 2, 3b and 4b reflect direct or conceptual replications of the pre‐registered study designs. Minor deviations from pre‐registered analysis plans are detailed in Appendix S1. Exploratory analyses are clearly labelled in the manuscript. Changes in the terminology used to describe variables are highlighted in Appendix S2. The significant correlations between identity fusion and willingness to hire in studies 1a, 2 and 3a, which are tested as part of larger mediation models tested here, have previously been reported in a separate manuscript that focuses on currently incarcerated people and public acceptance of people involved in the criminal justice system (Newson et al., 2024). The underlying mechanisms and experimental designs are reported here for the first time.

## STUDIES 1A AND 1B: BEREAVEMENT EXPERIENCES AS A SOURCE OF FUSION AND WILLINGNESS TO HIRE

The first study examined the impact of bereavement experiences on people's levels of fusion to a formerly incarcerated person and their willingness to offer employment. We sought to expand on Reich et al.'s (2024) experimental study design, in which the authors manipulated a job applicant's backstory, presenting a formerly incarcerated person's motivation to stay out of trouble with the law based on either (a) having gone through a transformative bereavement experience in prison, (b) having witnessed someone else going through a transformative bereavement experience in prison or (c) no particular experience. They found that both direct and indirect bereavement cues increased fusion and, indirectly, willingness to hire. In studies 1a and 1b, we tested this design using US and UK samples, respectively. As an extension of Reich et al., we sought to (a) increase the ecological validity of the original design by recruiting participants with hiring experience and (b) improve the robustness of the analyses by accounting for various known confounds for attitudes towards formerly incarcerated persons (i.e. personal contact to formerly incarcerated persons, past victimization of crime, political ideology, open‐mindedness and demographics).

### Method

#### Samples

For Study 1a we analysed the data of *N* = 320 US nationals (*M*_age_ = 44.84, *SD*_age_ = 13.06), the majority of whom were male (56.6%) and White (82.5%). For Study 1b, we analysed a sample of *N* = 327 UK nationals (*M*_age_ = 42.09, *SD*_age_ = 11.17), the majority of whom were male (54.7%) and White (92%).

In all studies, participants were recruited from the crowdsourcing platform Prolific and received monetary compensation. All participants had hiring experience and had never been to prison. Personal exposure to specific types of experiences that are referred to in the experimental conditions of s (i.e. bereavement experiences in S1, natural disaster experiences in S2, sporting disappointment experiences in S3) was not a recruitment criterion, based on the assumption that the experience was implicitly shared within the respective participant populations. The implications of this are discussed. Ethics for all studies were obtained from the School of Anthropology and Conservation ethics board at the University of Kent (all individual ethic IDs are listed in Appendix S1). Sample sizes were determined based on a‐priori power analyses of the pre‐registered hypotheses (using G*Power, Faul et al., 2007) to obtain .80 power and detect small‐medium effect sizes at the standard .05 alpha error probability, plus 10% oversampling to account for potential missing data (see the respective pre‐registration documents for full details).

#### Procedure and materials

All study materials and stimuli can be found in Appendix S2. Participants were randomly allocated to one of three experimental conditions (direct experience, indirect experience, control). These were introduced using vignettes based on Reich et al. (2024). All participants read an introduction text putting them into the shoes of an employer in a job‐hiring scenario in which the applicant had served time in prison and disclosed additional information during the interview. In the *direct experience* condition, participants learn that the applicant disclosed a transformative bereavement experience (their mother died of cancer while they were incarcerated). In the *indirect experience* condition, the applicant disclosed witnessing his cellmate's transformative bereavement experience, whereas no reference to a bereavement experience was made in the *control condition*. The conditions were dummy‐coded into categorical variables (1 = assigned to condition, 0 = not assigned to condition).

After completing an attention check, participants indicated how willing they were to hire the applicant (1 = totally unwilling, 6 = totally willing). Next, we measured identity fusion to the applicant using four items of Gómez et al.'s (2011) verbal identity fusion scale (‘I have a deep emotional bond with the applicant’, 1 = strongly disagree, 7 = strongly agree). To measure the extent to which participants' bereavement experiences were transformative, they were first asked to ‘take a moment to think about a past experience of loss and bereavement of family members in your own life. If you have not experienced the loss of a family member, think about a close friend or colleague that you have lost’. Then they responded to four items (‘This experience was personally transformative for me’, 1 = strongly disagree, 7 = strongly agree^1^; based on Buhrmester et al., 2018).

Additionally, participants completed measures previously associated with attitudes towards formerly incarcerated persons. These include the quantity of formerly incarcerated persons known to the participant (1 = none, 4 = many; Hirschfield & Piquero, 2010), political ideology via self‐placement on general, social and economic issues (0 = left, 10 = right, three items), open‐mindedness towards people's capacity to change (‘Everyone, no matter who they are, can significantly change their basic characteristics’, 1 = strongly disagree, 6 = strongly agree; eight items, Dweck, 2013) and whether the participant or their family have ever been a victim of crime (1 = Yes, 0 = No). Lastly, we measured basic socio‐economic characteristics, including age, gender (dummy coded as categorical variable 1 = male, 0 = non‐male), ethnicity (dummy coded as categorical variable 1 = White, 0 = non‐White), subjective socio‐economic status on a self‐placement scale (1 = worst off, 10 = best off; Adler et al., 1994), educational level (1 = no formal qualifications, 5 = postgraduate degree) and the number of hiring decisions participants were involved in (0 = none, 6 = more than 50).

#### Statistical analysis

SPSS v29 and Hayes' (2017) PROCESS macro were used for statistical analyses. Coefficients presented in text are standardized, and coefficients presented in figures are unstandardized to maximize the output available to readers. Measures previously linked to criminal justice outcomes were only included as covariates in our models if they showed significant bivariate correlations with outcome measures. As such, there are variations as to which control variables are included across studies. We explicitly state which variable was controlled for in each analysis (i.e. which displayed significant bivariate correlations with the outcome) and refer to the corresponding correlation table and full model results in the Supporting Information. Sample characteristics (incl. *n* of failed attention checks, demographic breakdown), descriptive statistics, internal reliability scores for multi‐item measures and bivariate correlation analyses, as well as the full results for all analyses reported in the manuscript can be found in Appendix S1.

### Results

#### Fusion is linked to direct bereavement cue and own bereavement experiences in S1a, but only to own bereavement experiences in S1b

First, we conducted a series of linear regression analyses to test whether fusion to the applicant was predicted by (1) the experimental conditions, (2) participants' rated transformativeness of a bereavement experiences, (3) and the respective interaction terms (i.e. whether ratings of transformativeness moderate the effects of the experimental conditions). The potential confounds considered in this study were age, gender, ethnicity, socio‐economic status, education, hiring experience, political ideology, open‐mindedness, contact with people who have been to prison and victim status. Variables displaying significant bivariate correlations with the respective dependent variables (identity fusion, willingness to hire) were added as controls in the regression analyses as indicated. All bivariate correlation coefficients can be found in the Appendix S1: Table A1.1 (Study 1a) and Table A2.1 (Study 1b).

Among the US sample (S1a), participants in the direct experience condition were significantly more fused to the formerly incarcerated person (*β* = 0.14, *p* = .031). This was in line with Reich et al.'s (2024) findings. However, there was no significant effect for participants in the indirect experience condition and those in the control condition (*β* = −0.02, *p* = .781), *F*(2, 317) = 3.57, *p* = .029, *R* ^2^ = .02 (see mean levels of fusion between conditions in Table 1). We also found that rating bereavement experiences as more transformative predicted higher levels of fusion to the formerly incarcerated person (*β* = 0.15, *p* = .009), in addition to the experimental effects and while controlling for political ideology and open‐mindedness, *F*(5, 314) = 7.06, *p* < .001, *R* ^2^ = .10 (see Appendix S1: Table A1.2 for full model results). However, the effects of the experimental conditions were not dependent on participants' transformative bereavement experiences, that is, there were no significant interactions (*p values* > .475).

**TABLE 1:** Descriptive statistics for outcome measures between experimental conditions in all studies.

Study	Study 1a (US)			Study 2 (US)			Study 3a (US)			Study 4a (US)	
Condition (*n*)	Direct (107)	Indirect (106)	Control (107)	Dysphoric (108)	Non‐dysphoric (106)	Control (105)	Dysphoric (99)	Non‐dysphoric (86)	Control (109)	Good prison conditions (131)	Bad prison conditions (131)
Identity Fusion	2.94 (1.24)	2.51 (1.23)	2.56 (1.38)	2.18 (1.03)	2.03 (0.86)	1.97 (0.95)	2.21 (0.93)	2.19 (0.94)	2.16 (0.83)	3.19 (1.54)	3.19 (1.29)
Participant experience transformativeness	5.62 (1.19)	5.22 (1.45)	5.58 (1.32)	3.68 (0.84)	3.72 (0.92)	3.54 (1.02)	3.44 (0.91)	3.41 (0.99)	3.37 (0.98)	6.05 (0.81)	6.07 (0.82)
Applicant experience transformativeness										5.68 (0.88)	6.00 (0.89)
Willingness to hire	4.21 (1.33)	4.02 (1.10)	3.95 (1.25)	4.79 (1.02)	4.59 (1.00)	4.57 (1.06)	4.59 (0.96)	4.62 (1.07)	4.60 (0.98)	4.58 (0.89)	4.59 (0.95)
Perceived chances to desist							3.58 (0.88)	3.52 (0.84)	3.58 (0.81)	3.82 (0.69)	3.80 (0.38)
**Study**	**Study 1b (UK)**						**Study 3b (UK)**			**Study 4b (UK)**	
**Condition (*n*)**	**Direct (110)**	**Indirect (112)**	**Control (105)**				**Dysphoric (105)**	**Non‐dysphoric (90)**	**Control (108)**	**Good prison (131)**	**Bad prison (135)**
Identity Fusion	3.07 (1.14)	3.17 (1.29)	2.95 (1.11)				2.35 (0.87)	2.57 (0.87)	2.40 (0.78)	2.94 (1.23)	2.86 (1.17)
Participant experience transformativeness	5.25 (1.31)	5.38 (1.13)	5.42 (1.23)				3.17 (0.89)	3.38 (0.78)	3.24 (0.88)	6.01 (1.11)	5.97 (1.15)
Applicant experience transformativeness										5.75 (0.98)	5.95 (0.95)
Willingness to hire	4.47 (1.02)	4.16 (1.07)	4.27 (0.98)				4.56 (0.99)	4.71 (0.84)	4.56 (1.09)	4.17 (0.97)	4.22 (0.97)
Perceived chances to desist							3.95 (0.63)	3.83 (0.69)	3.78 (0.75)	3.67 (0.61)	3.56 (0.72)

*Note*: Reported statistics for outcome variables are means and, in parentheses, standard deviations.

We conducted the same analyses with the British sample (S1b), but did not find a significant effect of the experimental conditions on fusion (*p values* > .159), *F*(2, 324) = 0.99, *p* = .372, *R* ^2^ = .01 (see mean levels in Table 1). However, we replicated the finding that participants who rated bereavement experiences as more transformative were more fused (*β* = 0.15, *p* = .004), while controlling for open‐mindedness and victim status, *F*(5, 321) = 8.96, *p* < .001, *R* ^2^ = .12. No significant interaction effects were found (*p values* > .294) (see Appendix S1: Table A2.2 for full regression analysis results).

#### Identity fusion predicts willingness to hire

**FIGURE 1:**
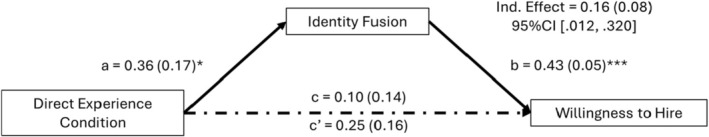
Direct and indirect effects of the direct bereavement condition on willingness to hire via identity fusion in Study 1a. a, b, c and c′ are path coefficients representing unstandardized regression weights and standard errors (in parentheses). *p < .05, ***p < .001.

Next, we conducted multiple linear regression analyses to test for both direct and indirect effects of the experimental conditions (via identity fusion) on participants' willingness to hire the formerly incarcerated person for a job. For the US sample (S1a), there were no direct effects of the experimental conditions (*p values* > .175), and participants' rated transformativeness of bereavement experiences was not linked to their willingness to hire (*p* = .958). Instead, we found that identity fusion significantly predicted willingness to hire (*β* = 0.46, *p <* .001), even when controlling for open‐mindedness, political ideology, contact with incarcerated persons, victim status and SES, *F*(9, 310) = 18.67, *p* < .001, *R* ^2^ = .35^2^ (see Appendix S1: Table A1.3 for full regression analysis results). Replicating Reich et al. (2024), we found a significant indirect effect of the direct bereavement condition, indicating that US participants who learned that the applicant had lost their mother to cancer while in prison were more likely to be fused to them and, relatedly, more willing to hire them (see Figure 1).

**FIGURE 2:**
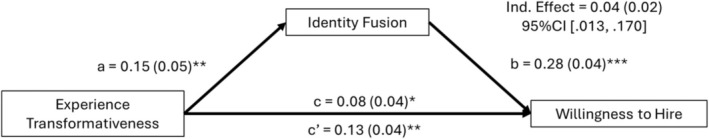
Direct and indirect effects of transformative experience on willingness to hire via identity fusion in Study 1b. a, b, c and c′ are path coefficients representing unstandardized regression weights and standard errors (in parentheses). *p < .05, **p < .01, ***p < .001.

For the UK sample (S1b), there was also no direct effect of the experimental conditions on willingness to hire (*p values* > .089). However, we found that participants who rated their bereavement experiences as more transformative were more likely to say they would hire the formerly incarcerated person (*β* = 0.12, *p =* .015), as were participants with higher levels of identity fusion (*β* = 0.32, *p <* .001) controlling for open‐mindedness, political ideology, age and SES, *F*(8, 318) = 15.00, *p* < .001, *R* ^2^ = .27 (See Appendix S1: Table A2.3 for full model results of the regression analyses predicting willingness to hire). In the absence of experimental effects, we conducted exploratory analyses whether the association between transformativeness ratings and willingness to hire could be explained (i.e. mediated) by levels of identity fusion. We found that British people who rated their bereavement experiences as more transformative were more strongly fused to formerly incarcerated persons and more likely to offer employment (Figure 2).

#### S1 summary

In S1a, we partially replicated Reich et al.'s (2024) main findings. That is, people in the United States, acting as prospective employers in a hiring scenario, were more fused and willing to hire, a formerly incarcerated person who disclosed a firsthand bereavement experience during a job interview. Participants' ratings of their own bereavement experiences as transformative predicted stronger fusion to formerly incarcerated persons but independently from the applicant's disclosed experience. A replication attempt with UK citizens (S1b) showed that cues of bereavement experiences of any kind (direct or indirect) were inconsequential and that levels of fusion and willingness to hire were instead both directly and indirectly linked with participants' ratings of their own bereavement experiences.

## STUDY 2: SHARED NATIONAL EXPERIENCES AS A SOURCE OF FUSION, WILLINGNESS TO HIRE AND PERCEIVED CHANCES TO DESIST

As the disclosure of a bereavement experience was able to trigger fusion in S1a, we tested whether the same could be achieved with references to shared group experiences. Rather than distinguishing between direct and indirect experiences, in Study 2, we focused on potential differences between dysphoric and non‐dysphoric experiences, given the theorized differences in transformative potential (Jong et al., 2015; Whitehouse, 2018). Participants were again allocated a hypothetical role as an employer to test if cues of shared group experiences, particularly dysphoric ones, were linked to identity fusion and (indirectly) willingness to hire and perceived chances to desist and whether such effects would interact with participants' own transformative experiences. Cues were disclosed as part of a background check of prospective applicants' social media profiles, which were edited to either refer to a dysphoric or a non‐dysphoric experience in a national context, or no shared experience at all. We chose Hurricane Katrina as a reference event for a shared dysphoric experience, as it is one of the most impactful natural disasters in modern US history (Deane et al., 2016), and the capacity of disasters to strengthen group ties (Segal et al., 2018; Wright et al., 1990). For a non‐dysphoric shared event, we referred to a federal holiday, Labor Day. We did not use this design in a UK context due to a lack of events with comparable scope.

### Method

#### Sample

Given the content of the experimental manipulation, we pre‐screened the sample to include only US nationals who were at least 10 years old when Hurricane Katrina happened. The final sample consisted of *N* = 319 participants (*M*_age_ = 42.49, *SD*_age_ = 11.69), the majority of whom were female (57.7%) and White (74%).

#### Procedure and materials

Participants were randomly allocated to one of three conditions (dysphoric experience, non‐dysphoric experience, control) and initially read the same introduction text as for S1a, but were told that they would look through an excerpt of the formerly incarcerated person's social media presence (Facebook) to gain a better understanding of them. In the two experimental conditions, participants saw a cue that emphasized the applicant's national identity (a picture prominently featuring the American flag). In the *dysphoric* condition, participants additionally saw a post about the impact of Hurricane Katrina, featuring pictures of a memorial for the victims and an archival picture of the initial destruction. In the *non‐dysphoric* condition, participants saw a post about a national federal holiday, Labor Day, featuring pictures of a memorial for American workers and a picture of a Labor Day parade. In the *control condition*, participants saw a post featuring an unidentifiable flag and another about attending a local music festival. All conditions featured two identical neutral posts.

Participants completed the same measures of willingness to hire, identity fusion, criminal justice covariates and demographic characteristics described for S1. Additionally, we measured how participants perceived the chances of the formerly incarcerated person to ‘stay out of trouble with the law’ (hereafter referred to as ‘perceived chances to desist’ for brevity) (1 = poor, 5 = excellent) as an outcome variable. Experience transformativeness was measured with the same scale as in S1, but participants were prompted to think of personal experiences of environmental disasters rather than bereavement experiences before responding to the individual items. We also included a measure of symbolic patriotism (‘How good does it make you feel when you see the American flag flying?’, 1 = Not at all, 5 = Extremely; Huddy & Khatib, 2007), to account for attachment to the group context. Lastly, we added the Social Desirability Inventory (16 items, Stöber, 2001) to ensure that support for a marginalized group was not explained by participants' tendencies to give socially acceptable answers.

### Results

#### Experience transformativeness, not experience cues, predicts fusion and (indirectly) willingness to hire and perceived chances to desist

The potential confounds considered in this study were age, gender, ethnicity, socio‐economic status, education, hiring experience, political ideology, open‐mindedness, contact with people who have been to prison, victim status, social desirability and symbolic patriotism. Variables displaying significant bivariate correlation with the respective dependent variable (identity fusion, willingness to hire) were added as controls in the regression analyses as indicated. A table of bivariate correlation coefficients can be found in the Appendix S1: Table B1. As in Study 1, a series of multiple linear regression analyses were conducted to predict fusion, willingness to hire and perceived chances to desist. Results showed no significant effects of the experimental conditions on the level of fusion, *F*(2, 316) = 1.39, *p* = .250, *R* ^2^ = .01. However, when adding the measures of how transformative participants rated their natural disaster experiences to the model, participants with stronger ratings were more fused (*β* = 0.21, *p <* .001), controlling for open‐mindedness, contact to formerly incarcerated persons, symbolic patriotism, gender, hiring experience, and social desirability, *F*(9, 309) = 5.60, *p* < .001, *R* ^2^ = .14 (see Appendix S1: Table B2 for full model results of regression analyses predicting identity fusion). The experimental conditions also had no significant direct effects on participants' willingness to hire, *F*(2, 316) = 1.42, *p* = .244, *R* ^2^ = .01, nor their perception that the applicant could desist from crime, *F*(2, 316) = 2.84, *p* = .060, *R* ^2^ = .02. When adding identity fusion, ratings of experiences as transformative and covariates to the model predicting willingness to hire, identity fusion was the strongest predictor^3^ (*β* = 0.25, *p <* .001), *F*(11, 307) = 7.92, *p* < .001, *R* ^2^ = .22 (see Appendix S1: Table B3 for full model results). Likewise, in the model for perceived chances to desist, *F*(11, 307) = 8.70, *p* < .001, *R* ^2^ = .18, identity fusion was the key predictor (*β* = 0.27, *p <* .001). Participants' transformative experiences related to natural disasters were not associated with either outcome variable (*p values* > .298) (see Appendix S1: Table B4 for full model results).

**FIGURE 3:**
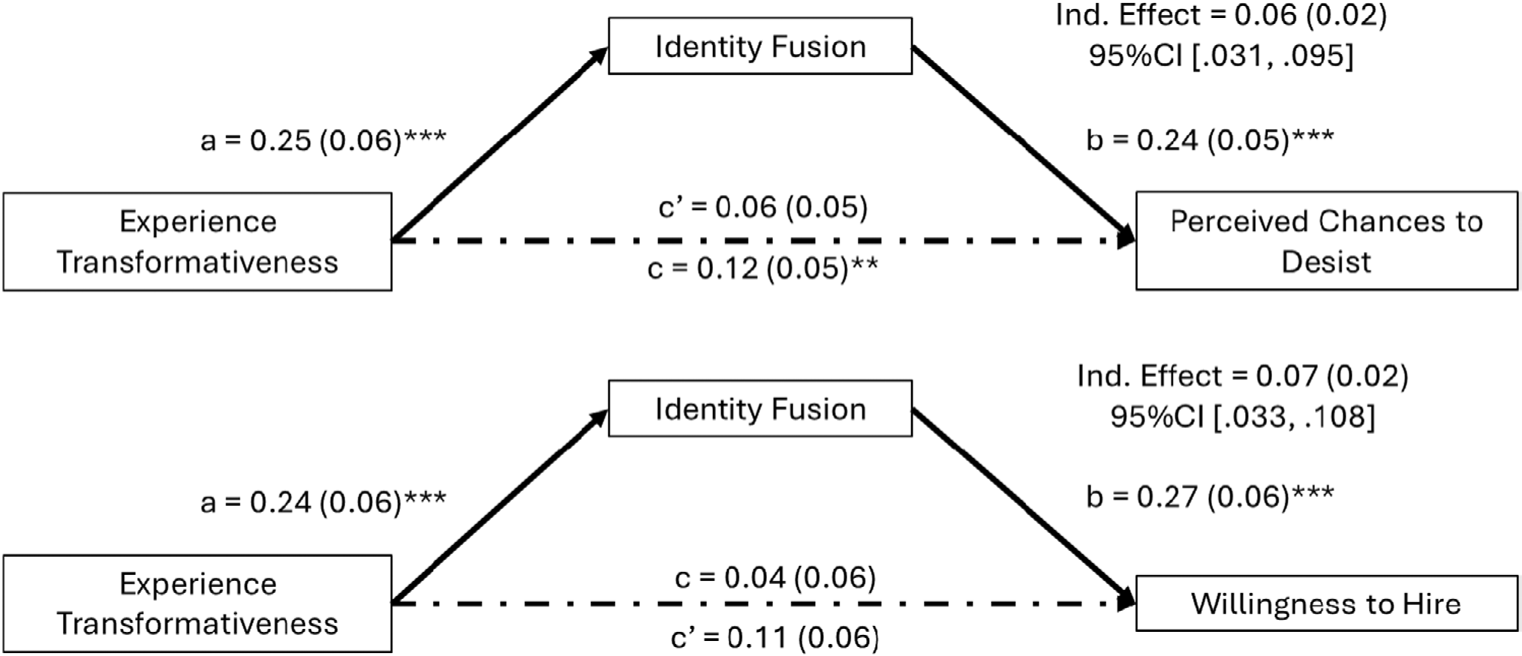
Direct and indirect effects of transformative natural disaster experiences on indicators of perceived chances to desist and willingness to hire in Study 2. a, b, c and c′ are path coefficients representing unstandardized regression weights and standard errors (in parentheses). **p < .01, ***p < .001.

We tested for potential indirect effects of transformative experiences via identity fusion, similar to those found in Study 1b. Mediation analyses revealed significant indirect effects of transformative experiences on both outcome variables, such that participants with stronger transformative natural disaster experiences were more strongly fused to a formerly incarcerated person. This was in turn linked to their willingness to hire and their perception that the applicant can desist (see Figure 3).

## STUDIES 3A AND 3B: SHARED SPORTS EXPERIENCES AS SOURCES OF FUSION, WILLINGNESS TO HIRE AND PERCEIVED CHANCES TO DESIST

In Study 3, we examined a social context that is known for its potential to evoke strong emotional responses and social bonding (Depetris‐Chauvin et al., 2020; Newson et al., 2016, 2021); passion for sport among American Football fans in the United States and football fans in the United Kingdom, respectively. Studies 3a and 3b thus examined how cues of sport experiences (dysphoric or non‐dysphoric) might influence participants' fusion to, and willingness to hire, a formerly incarcerated person. The design and procedures were similar to those reported in Study 2 (see Appendix S2).

### Methods

#### Samples

Given the content of the experimental manipulation, we pre‐screened the samples to include only people who consider themselves sports fans and engage with the sport (e.g. watching/playing the sport). For S3a, we analysed the data of *N* = 294 US American Football fans (*M*_age_ = 40.92, *SD*_age_ 12.47), the majority of whom were male (96.9%) and white (76.2%). For S3b, we analysed the data of *N* = 303 UK football fans (*M*_age_ = 41.12, *SD*_age_ 14.25), the majority of whom were male (80.5%) and white (82.5%).

#### Procedure and materials

Compared to S2, vignette materials differed concerning identity and experience cues. The experimental conditions contained a post about attending a sport training session to highlight the shared interest in sports. In the *dysphoric* condition, the applicant also posted about attending a high‐stakes game in which their team entered as the clear underdog and ended up dramatically losing the game. In the *non‐dysphoric* condition, the applicant posted about attending their team's game, having a meal at the stadium, and an unlucky result with little associated affect. In the *control condition*, the applicant posted about attending an event from a running club and trying out a new recipe (materials in Appendix S2: C).

Participants completed the same measures as detailed for S2. Transformative experiences were measured with reference to ‘past experiences of bitter sport disappointments and defeats of your favourite team’. We also captured participants' passion for the sport by asking how strongly they supported their favourite team (1 = Not at all, 5 = Massive fan).

### Results

#### Fusion and willingness to hire are unrelated to experimental cues, experience transformativeness in S3a

The potential confounds considered in this study were age, gender, ethnicity, socio‐economic status, education, hiring experience, political ideology, open‐mindedness, contact with people who have been to prison, victim status, social desirability and sports fandom. Variables displaying significant bivariate correlation with the respective dependent variable (identity fusion, willingness to hire) were added as controls in the regression analyses as indicated. All bivariate correlation coefficients can be found in the Appendix S1: Table C1.1 (Study 3a) and Table C2.1 (Study 3b). In the US sample (S3a), we found no effects of the experimental conditions on identity fusion, *F*(2, 291) = 0.07, *p* = .931. There were also no effects of how transformative participants rated their own experiences of sports defeat or sport fandom (*p values* > .315). Instead, participants who had more contact with formerly incarcerated persons (*β* = 0.25, *p* < .001) and those who did not describe themselves as male (*β* = −0.14, *p* < .001) tended to be more fused, *F*(8, 285) = 3.86, *p* < .001, *R* ^2^ = .10. Consequently, there were no significant interactions between experimental conditions and ratings of sport experiences as transformative or fandom (*p* values > .197) (see Appendix S1: Table C1.2 for full model results). In S3a, there were also no significant experimental effects on willingness to hire, *F*(2, 291) = 0.02, *p* = .978, *R* ^2^ = .00, and perceived chances of the applicant to desist, *F*(2, 291) = 1.24, *p* = .884, *R* ^2^ = .00. When adding the other predictor variables, identity fusion emerged as the key predictor of willingness to hire (*β* = 0.31, *p* < .001, *F*(10, 283) = 9.17, *p* < .001, *R* ^2^ = .25) and perceived chances to desist (*β* = 0.35, *p* < .001, *F*(8, 285) = 7.74, *p* < .001, *R* ^2^ = .18), whereas ratings of sport experiences as transformative and fandom were not significant predictors (*p* values > .143) while controlling for effects of open‐mindedness, incarcerated person contact, age, past victimization, and socio‐economic status^4^ (see Appendix S1, Table C1.3 for full model results). Thus, neither participants' rated transformativeness of sport experiences nor those observed among the formerly incarcerated persons elicited fusion, willingness to hire or perceived chances to desist from crime among US American Football fans.

#### Experience cues predict fusion and willingness to hire/perceived chances to desist, depending on sport experience transformativeness in S3b

**FIGURE 4:**
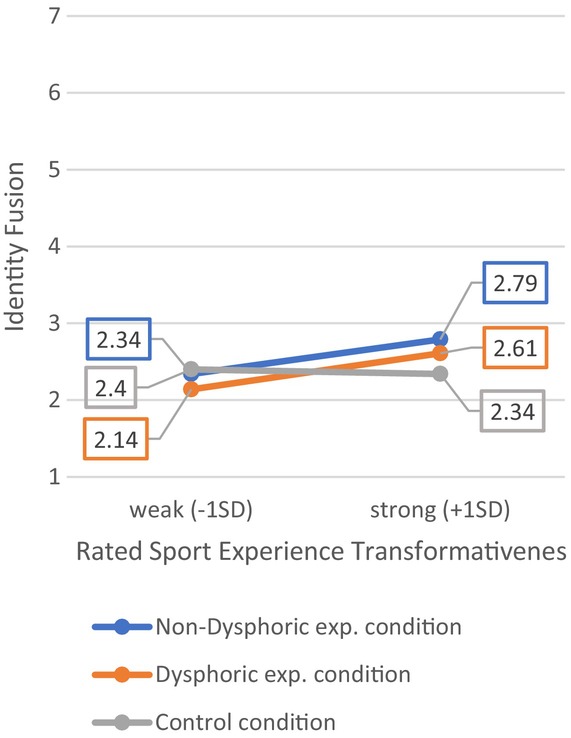
Conditional effects of experimental conditions on identity fusion at ±1 *SD* of sport experiences transformativeness in Study 3b.

In the British sample (S3b), there were no direct effects of the experimental conditions on identity fusion, *F*(2, 300) = 1.88, *p* = .155, *R* ^2^ = .01. However, we found that stronger ratings of sports experiences as transformative predicted fusion to the applicant (*β* = 0.25, *p* < .001), controlling for open‐mindedness, victim status and social desirability scores, *F*(7, 295) = 4.27, *p* < .001, *R* ^2^ = .09. Most importantly, there were significant interaction effects between both experimental conditions and the transformativeness of sport experiences, such that participants who were exposed to the cues of dysphoric and non‐dysphoric football experiences were significantly more fused to the applicant when they rated their own sports experiences as more transformative, *F*(9, 293) = 4.20, *p* < .001, *R* ^2^ = .11 (Figure 4, see Appendix S1: Table C2.2 for full model results).

**FIGURE 5:**
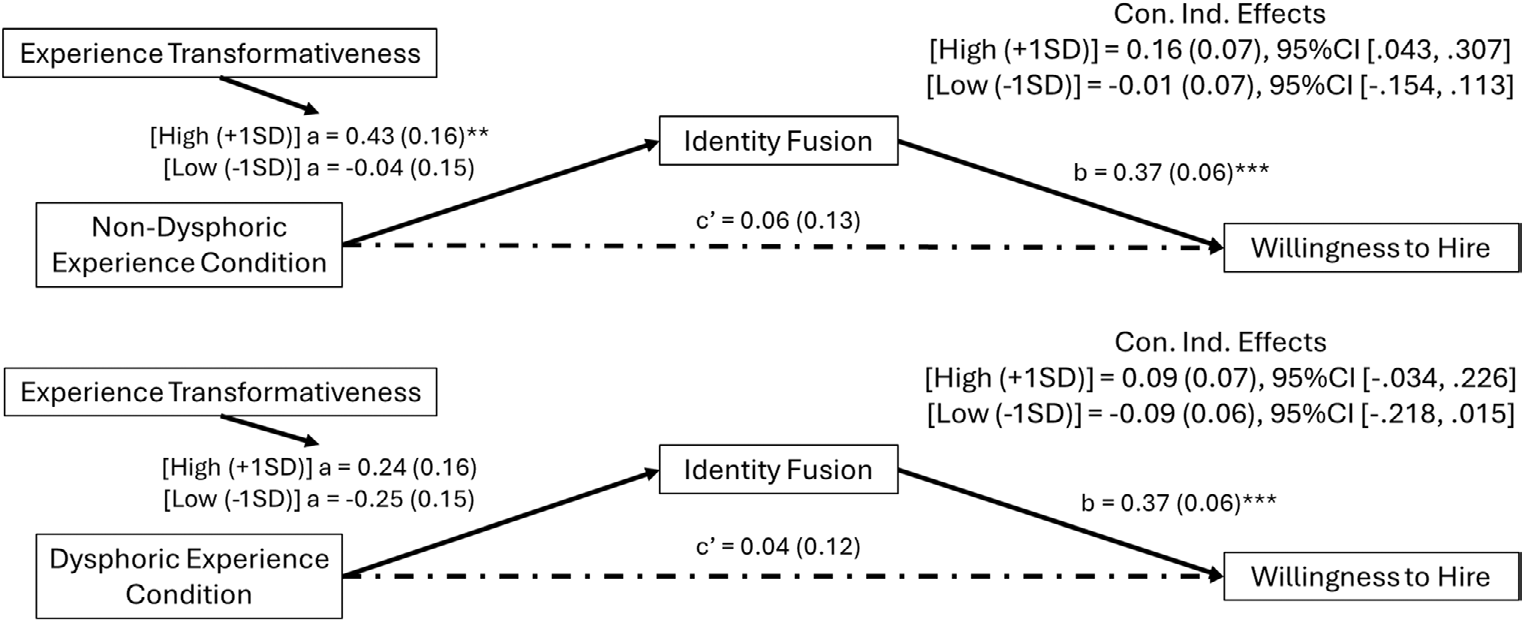
Direct and conditional indirect effects of experimental conditions on willingness to hire via identity fusion. a, b and c′ are path coefficients representing unstandardized regression weights and standard errors (in parentheses). *p < .05, **p < .01, ***p < .001.

Predicting UK participants' willingness to hire the applicant, regression analysis showed no effects of the experimental conditions, *F*(2, 300) = 0.76, *p* = .469, *R* ^2^ = .01. However, when testing for conditional indirect effects via identify fusion the model was significant, *F*(8, 293) = 9.67, *p* < .001, *R* ^2^ = .21. As shown in Figure 5, we found the anticipated significant effects of identity fusion, as well as conditional indirect effects of the experimental conditions (moderated mediation index, dysphoric exp. condition = 0.11, SE = 0.06, 95% CI [0.009, 0.225]; regular exp. condition = 0.10, SE = 0.06, 95% CI [0.003, 0.217], see Appendix S1: Table C2.3 for full model results). Among those who rated their experiences of sport defeat as highly (+1*SD*) transformative, exposure to an applicant's sports experiences was associated with stronger fusion, and indirectly, with more willingness to hire, compared to those who rated their experiences as less (−1*SD*) transformative (see Figure 5). Highly similar patterns were observed for the outcome variable *perceived chances to desist*, as participants in the experimental conditions were more fused to the applicant and thought he could desist if they rated their experiences as more, rather than less, transformative (see Appendix S1: Table C2.4 for full model results).

#### S3 summary

US American Football fans (S3a) were overall not receptive to cues of shared experiences related to sports, nor did their own sport experiences and fandom contribute to identity fusion, willingness to hire or perceived chances to desist. In contrast, among British football fans (S3b), self‐reported levels of bonding with a formerly incarcerated person who disclosed painful or intense sports experiences were dependent on how participants rated their own experiences of sports defeat, wherein those who thought them more transformative were more fused and more willing to hire the applicant. Given the similarities in design and sample demographics, it is plausible that national differences hold the key to explaining differences between the studies. Although American Football and football are the most popular spectator sports in their respective countries (World Population Review, 2024), the importance of fans' sport experiences likely reflects national and regional sport cultures and histories, which are hardly comparable between these countries (Brown, 2007). National differences in receiving communities' attitudes towards the criminal justice system and reintegration mechanisms could also play a role and are further addressed in the general discussion.

## STUDY 4: THE OVERLAP BETWEEN EMPLOYERS' AND APPLICANTS' EXPERIENCE TRANSFORMATIVENESS AS A SOURCE OF FUSION AND WILLINGNESS TO HIRE

Studies 1–3 demonstrated how challenging it is to elicit identity fusion and reintegration support via experience cues; instead, they suggested that rating experiences as transformative was vital to activate a path to bonding. Although these studies focussed on the effects of life experiences in a shared social context, participants' views of their own experiences as transformative were also linked to fusion and support without indicators that targets (i.e. job applicants) shared an experience in this particular context (S1a, S2). The theorized shared component of the transformative path could instead have been reflected in an overlap of the transformativeness of experiences. It is plausible that people who have never been to prison would evaluate time served in prison itself as highly transformative; thus, those who are aware of an experience they consider highly transformative may find it easier to fuse to and willing to hire a formerly incarcerated person. This led us to conduct a final set of studies that focussed in more detail on participants' perceptions of formerly incarcerated persons' prison experience.

With samples of US (S4a) and UK (S4b) nationals, we tested the hypotheses that people who rate a life experiences of any kind (negative or positive, in any context) as highly transformative find it easier to fuse to, and thus be more willing to hire an formerly incarcerated person. We tested whether this association is dependent on (i.e. moderated by) their perception of how transformative the applicant's prison experience was. We manipulated the information disclosed about the applicant's prison conditions to describe them as either particularly bad or good, suspecting that more challenging prison conditions would be perceived as more transformative and, thus, lead to stronger bonding. This assumption was based on the premise that large sections of the public perceive ‘tough on crime’ policies and adverse prison conditions as successful motivators to desist from crime (Maahs & Pratt, 2016; Mauer, 1999), even though the effectiveness of such approaches is disputed in the academic literature (Dölling et al., 2009).

### Methods

#### Samples

For S4a, we analysed a sample consisting of *N* = 262 American citizens (*M*_age_ = 46.55), the majority of whom were female (50.4%) and White (78.2%). For S4b, we analysed a sample of *N* = 266 UK nationals (*M*_age_ = 45.84), the majority of whom were male (50.4%) and White (92.5%).

#### Procedure and materials

At the start of the survey, participants were asked to recall an impactful event in their life and describe it in a few sentences (to ensure engagement). This was counterbalanced (i.e. half the sample was asked to recall a positive event, the other half to recall a negative event) to ensure the sample was not biased towards either type of experience. After this, participants completed Buhrmester et al.'s (2018) transformative experience measure to indicate how transformative they rate the chosen event. Next, participants completed questions about their demographics and the same control variables as described in Study 3 as a filler task. They were then randomly allocated to one of two conditions where vignettes described the hiring scenario from Study 1 (using the interview disclosure design) and the applicant disclosed either that he is motivated to stay out of trouble based on his experience in a particularly good or a particularly bad prison. The vignette in the *good‐prison condition* contained a description of the applicant's prison conditions as ‘overall very good, no overcrowding, clean toilets and bathrooms, plenty of opportunities for sports and education’. He also mentions that ‘the atmosphere in the prison was very calm, there was not much violence or self‐harm among inmates’. In comparison the bad‐prison condition included a description of the prison as ‘overall very poor, overcrowded, dirty toilets and bathrooms, almost no opportunities for sports and education’. He also mentions that ‘the atmosphere in the prison was very tense, that there was a lot of violence and self‐harm among inmates’. After reading one of the experimental vignettes, participants completed Buhrmester et al.'s (2018) four‐item transformative experience scale, but this time, they rated the applicant's prison experience (i.e. how transformative they considered the experience to have been for the applicant). Overall, participants completed the scale twice, once to rate the transformativeness of their chosen personal life experience and once to rate the transformativeness of the applicant's prison experience. For the UK sample the scale showed low internal reliability (*α* = .59), and thus, we used a single‐item indicator for transformative experiences (‘This experience was personally transformative for me/him’) in S4b. Finally, as with the previous studies, participants completed the remaining outcome measures of willingness to hire and perceived chances to desist.

### Results

#### Effects of participants' experience on fusion and hiring depend on perceived transformativeness of applicant's experience and prison condition in S4a

The potential confounds considered in this study were age, gender, ethnicity, socio‐economic status, education, hiring experience, political ideology, open‐mindedness, contact with people who have been to prison, victim status and social desirability. Variables displaying significant bivariate correlation with the respective dependent variable (identity fusion, willingness to hire) were added as controls in the regression analyses as indicated. All bivariate correlation coefficients can be found in the Appendix S1: Table D1.1 (Study 4a) and Table D2.1 (Study 4b).

As hypothesized, we found that US participants in S4a who rated their chosen experience as more transformative were more fused to the applicant, *F*(1, 260) = 4.38, *p* = .037, *R* ^2^ = .02. Moreover, there was a significant interaction between ratings of participants' own experiences and the prison experience of the applicant as transformative (*β* = 1.20, *p* = .040, *F*(7, 254) = 9.50, *p* < .001, *R* ^2^ = .21), while controlling for open‐mindedness, victim status, social desirability and ethnicity (see full model results in Appendix S1: Table D1.2). Those participants who rated their experiences as more transformative were more fused to the applicant, but only if they rated the applicant's prison experience as highly transformative, too.

**FIGURE 6:**
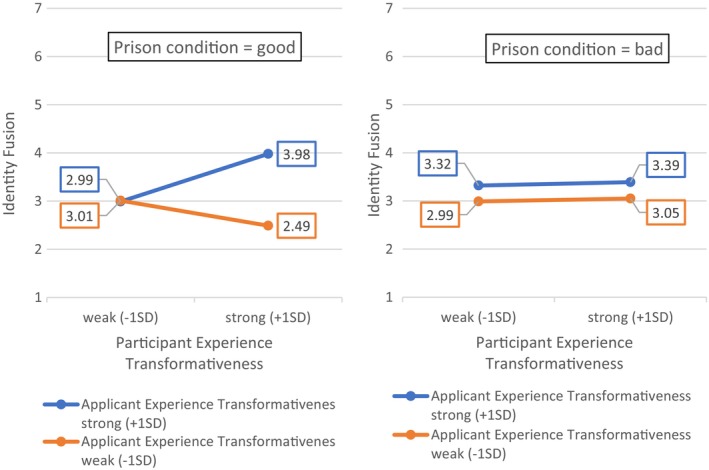
Conditional effects of (±1*SD*) participant experience transformativeness on identity fusion at ±1*SD* of applicant experience transformativeness between the ‘good prison’ and ‘bad prison’ conditions.

The perception of the applicant's prison experience was further impacted by the descriptions of prison conditions in the experimental conditions. Participants in the *bad prison condition* rated the applicant's experience as significantly more transformative (*M* = 6.00, *SD* = 0.89) than those in the *good prison condition* (*M* = 5.68, *SD* = 0.88), *t*(260) = −2.89, *p* = .004. Consequently, we conducted exploratory analyses to examine the possibility of a three‐way interaction between participants' experience transformativeness, applicants' experience transformativeness, and prison condition (i.e. moderated moderation) to predict identity fusion. The resulting model showed a significant three‐way interaction (*B* = −0.51, *SE* = 0.21, *p* = .015, *F*(11, 250) = 6.82, *p* < .001, *R* ^2^ = .23) (see Appendix S1: Table D1.3 for full model results). The conditional effects (as shown in Figure 6) revealed that the perceived overlap between participants' own and applicants' perceived transformation boosted identity fusion, particularly in the *good prison* condition.

**FIGURE 7:**
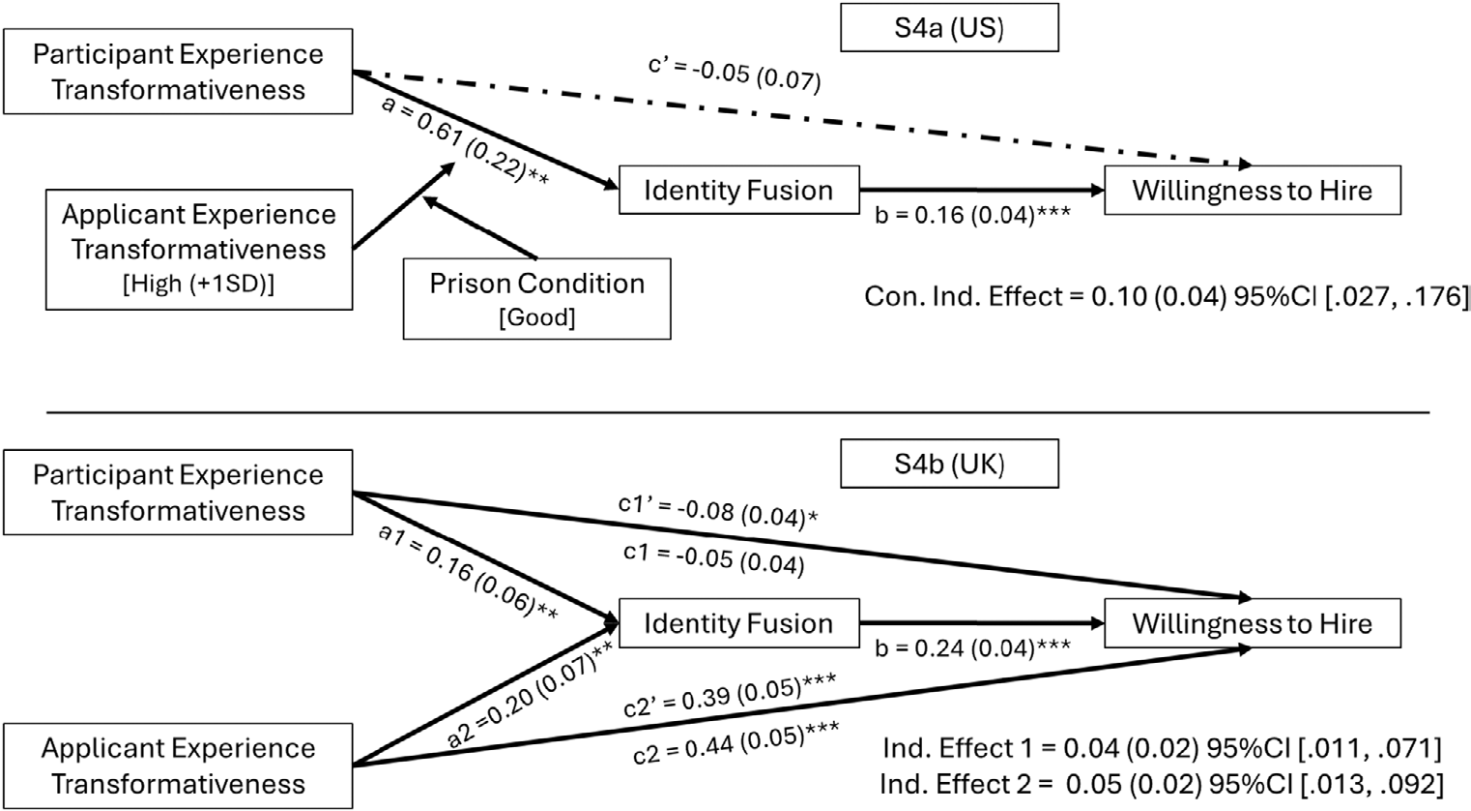
Direct and conditional indirect (S4a)/indirect (S4b) effects of experience transformativeness on willingness to hire. a, b, c and c' are path coefficients representing unstandardized regression weights and standard errors (in parentheses). The indirect effects for study S4b were estimated in two separate models. *p < .05, **p < .01, ***p < .001.

Next, we tested this interaction as part of conditional moderated mediation models to predict participants' willingness to hire and perceived chances to desist, controlling for open‐mindedness, victim status, social desirability and ethnicity. As shown in Figure 7, we found no significant direct effects of transformative experiences on willingness to hire, as well as the anticipated significant positive effect of identity fusion. Importantly, the index of moderated mediation was significant (*B* = −0.08, *SE* = 0.04, 95% CI [−0.168, −0.016]), as was the index of conditional moderated mediation for the *good prison condition* (*B* = 0.08, *SE*, 0.03, 95% CIs [0.026, 0.151], see Appendix S1: Table D1.3 for full model results). Those in the good prison condition, who perceived the applicant's experience as highly transformative and rated their own life experience as highly transformative were more strongly fused to the applicant and were more likely to offer him a job in the hiring scenario. A highly similar pattern of effects was observed for the same model predicting perceived chances of the applicant to desist (see Appendix S1, Table D1.3 for full model results).

#### Participant and applicant experience transformativeness independently predict fusion, willingness to hire and chances to desist in S4b

In the UK sample (S4b), we replicated the hypothesized effect that ratings of participants' experiences as transformative predict fusion to the applicant (*β* = 0.16, *p* = .008). Contrary to our hypothesis, there was no significant interaction between the participant and applicant experience ratings (own × perceived, *p* = .738). Instead, we found that rating applicant's experience as more transformative independently contributed to levels of fusion (*β* = 0.17, *p* = .006), while controlling for open‐mindedness and gender, *F*(4, 261) = 10.56, *p* < .001, *R* ^2^ = .14 (see Appendix S1: Table D2.2 for full model results). Moreover, how transformative an applicant's experience was rated did not differ significantly between the prison conditions, as participants considered time served in a bad prison as marginally more transformative (*M* = 5.95, *SD* = 0.95) than time served in a good prison (*M* = 5.75, *SD* = 0.98), *t*(264) = −1.69, *p* = .092.

Without the hypothesized interactions, we tested for simple direct and indirect effects of both own and perceived experience transformativeness via identity fusion on willingness to hire and perceived chances to desist from crime, respectively (controlling for open‐mindedness, political ideology and gender). As shown in Figure 7, fusion was associated with significantly higher willingness to hire. Participants' ratings of their *own* experiences had a small but significant negative effect on willingness to hire, meaning that participants who described their chosen life event as more transformative were less willing to hire the applicant. In contrast, evaluations of the applicant's experience had a strong positive effect, such that participants who evaluated the prison experience as more transformative were more willing to hire them. Importantly, both evaluations of own and perceived experiences had significant indirect effects via fusion, strengthening the positive effect of perceived experiences, and suppressing the negative effect of own experience ratings. A highly similar pattern emerged when testing the effects on perceived chances to desist (see Appendix S1, Table D2.3 for full model results).

#### S4 summary

The results in Study 4 provided further evidence that experiences that are rated as transformative, both participants' own and those of the applicant, are associated with fusion, willingness to hire and perceived chances to desist from crime. However, only among US participants did we find the hypothesized interdependent associations where fusion and willingness to hire hinged on high transformativeneness ratings of a applicant's time in prison. Contrary to our hypothesis, fusion and willingness to hire were predicted by describing an applicant's prison conditions as good, rather than bad, even though the latter was, on average, rated as more transformative. This raises questions as to whether dysphoria is as important for evaluating others' experiences, particularly when the context is not shared. Participants who considered their own experiences as more transformative may not have recognized the meaning of a dysphoric prison experience if they had not had one themselves. Another interpretation is that participants with stronger transformative experiences recognized similarities in the constructive nature of a supportive, rather than a dysphoric, prison experience. Among UK participants (S4b), experiences rated as transformative, both participants' own and the applicant's, contributed independently to fusion, willingness to hire and perceived chances to desist.

## GENERAL DISCUSSION

Across four studies and seven samples of receiving communities in the United States and the United Kingdom, we investigated the role of transformative experiences in forming opinions about formerly incarcerated persons. Firstly, we examined (H1) whether potential employers would become more fused to formerly incarcerated persons (job applicants) when they disclosed (direct vs. indirect; dysphoric vs. non‐dysphoric) personal experiences in various contexts (personal loss, group loss, sports defeat) and (H2) whether fusion, in turn, predicts their willingness to hire and their expectations that the applicant could desist from crime. While fusion consistently predicted the outcomes as hypothesized,^5^ we found limited support for H1. Results of Study 1a partially replicated Reich et al.'s (2024) finding that direct bereavement experience cues can lead to fusion in a US context, but we did not find direct experimental effects with a UK sample (S1b), nor in the follow‐up studies using examining different contexts (S2, S3a, S3b). Consequently, we only found evidence for a simple mediated relationship between experimental cues and willingness to hire via identity fusion (H3) in study 1a.

We had further hypothesized (H4) that the effect of perceived transformation on fusion would depend on (i.e. be moderated by) the extent to which participants recognized their own experiences as transformative. Initially (S1‐3), we focused on participants' experiences in a context that was relevant to the type of experiences disclosed by the applicant (bereavement, natural disasters, sports defeat). We found evidence for the hypothesis among a sample of UK football fans (S3b), where experimental cues of the applicant being a football fan (disclosing either a dysphoric or non‐dysphoric sports experience) lead to increased identity fusion and, indirectly, willingness to hire, only among those who rated personal experiences of sports defeat as highly transformative (adding conditional support for H3). We did not find such interaction effects among US sports fans (S3a), nor in the study contexts of individual (S1) and group loss (S2). Instead, we found that those who rated their own (bereavement and natural disaster) experiences as more transformative were more fused to the applicant, regardless of what was disclosed. This led us to test whether a sense of sharing transformative experiences was inferred by the participants (S4), such that employers' own experiences (of any kind, positive or negative) predict fusion when they (i.e. the experiences) and the targets' experiences (their time in prison) were evaluated as similarly (strongly) transformative. We found evidence for this interaction (H4) in the United States (S4a), but not the United Kingdom (S4b), where more transformative experiences of (hypothetical) employers' and applicants' independently contributed to stronger fusion.

Overall, this suggests that transformative experiences can be a pathway to identity fusion where the target is a stranger and a member of a stigmatized group, but our findings also raise questions as to the importance of dysphoria in the recognition of shared experiences, and to what extent the potency of experience contexts is bound to cultural relevance.

Research on the *shared dysphoria pathway* to identity fusion has robustly demonstrated how enduring a harrowing or challenging ordeal can lead to a lasting sense of connectedness with others. This phenomenon has been evidenced across varied contexts, including soldiers on the frontline who share the traumas of combat (Whitehouse et al., 2014), members of fraternities subjected to physically and emotionally intense hazing rituals (Whitehouse et al., 2017) and football fans united in the emotional aftermath of a bitter team defeat (Newson et al., 2021). These studies illustrate that shared dysphoria, or collective adversity, can be a profound mechanism for group bonding, fostering a sense of personal transformation tied closely to one's identity.

Building on this, our findings extend prior work by showing that (a) cues of others' dysphoric transformative experiences (i.e. recognizing that someone has undergone an ordeal without directly sharing the event) can foster a sense of bonding with a stranger (Jong et al., 2015; and first shown in an employment context by Reich et al., 2024). We also show that (b) recognition of one's own experiences as transformative is linked with fusion to a stranger, even (c) when there is only indirect awareness (S3b) or no awareness that a stranger might share experiences in a particular context (S1, S2, S4). This nuanced extension not only aligns with earlier work on the reflective and meaning‐making opportunities presented by transformative events (Jong et al., 2015; Whitehouse, 2018), but also foregrounds the role of the transformational process itself as a bonding mechanism. In particular, the results of S4 show that transformation of both parties can be important, but experiences can be very different in nature. Unlike traditional perspectives that emphasize the event as the central focus of autobiographical memory formation, this paper underscores the transformative journey as a potentially independent pathway for fostering connection and identity fusion.

Our work adds to previous literature suggesting that transformative experiences provide individuals with a unique opportunity to effectively ‘rewrite’ their sense of self (Newson et al., 2021; Whitehouse & Lanman, 2014). This rewriting process includes incorporating others who have undergone similar transformative experiences into their sense of identity. As Fivush (2011) highlights, autobiographical memory traditionally frames the self around discrete, salient events, anchoring the identity to specific, temporally bound moments. However, philosophical perspectives on the nature of the self‐highlight that identity is not solely defined by specific events, but by the continuity and narrative coherence of transformative processes (Paul, 2014 or Zahavi, 2005).

In this light, we argue that while discrete shared events contribute to identity fusion through autobiographical memory, the process of transformation itself holds equal, if not greater, weight in redefining the self. This framing aligns with theoretical insights into the ‘experiential self’, which views transformation as an ongoing narrative that bridges past, present and future identities (Neisser, 1988). By shifting focus to the experiential and reflective dimensions of transformation, our findings open new avenues for exploring how shared transformations—whether direct or indirect—shape relational and collective identity.

During interactions with stigmatized groups, dissimilar characteristics (incl. self‐defining experiences) are naturally salient and a commonality in transformativeness could be the lowest common denominator, powerful enough to elicit fusion. This echoes Echterhoff's (2012) idea of a subjective sense of sharing, formulated as part of his shared‐reality theory, that a person can experience a shared reality as long as they believe that their own inner state (here; feeling transformed) and the corresponding reference (a life‐changing event) match that of another person, regardless of whether this is actually the case. From that perspective, the results of S4 would suggest that US participants could see matching elements in their transformative experiences, which UK participants did not.

The different patterns of interactions between participants' own experiences and a formerly incarcerated person's perceived experiences in the study pairs invite considerations of cultural differences for the evaluation of formerly incarcerated persons and their personal experiences between the two national groups. American participants appeared more receptive to information about a job applicant's prison experience. In Study 1 they showed higher levels of fusion when the incarcerated person disclosed a first‐hand bereavement experience while incarcerated (S1a) and in Study 4, the perception of more transformative experiences was crucial for fusion, but only when it was disclosed that prison experiences were made in a positive institutional environment. In contrast, exposure to experience cues with reference to a shared national (S2) or sports context (S3a) did not elicit fusion among Americans. Unlike their US counterparts, British participants were not influenced by details about applicants' prison experiences, such as the disclosure of personal loss (S1b). Neither were evaluations of prison experiences impacted by descriptions of positive versus negative prison conditions (S4b). However, UK nationals picked up on sports cues, that is, whether an applicant was a fellow football fan or not, and when he was depicted as one, those with more transformative fan experiences were more fused and willing to hire (S3b). The fact that US and UK samples reported similar strength transformative experiences in all respective study pairs indicates that result patterns reflect cultural differences as to how cues and contexts were perceived as relevant in a reintegration scenario, rather than sampling biases.

For context, in Europe and particularly the United Kingdom, football has a long history with fandom associated with meaningful geographical, social and political differences (Spaaij & Viñas, 2013; Testa & Armstrong, 2010). Thus, public engagement with a team's performance (as cued in S3) was likely a more meaningful social cue among UK nationals, and less so among US nationals. Conversely, US samples' receptiveness to information about applicants' adverse prison experiences could reflect their familiarity and awareness of this issue. Data suggest that most US citizens are aware that incarcerated persons regularly experience violence, self‐harm, overcrowding, or inadequate care, and more than 1 in 4 categorize average US prison conditions as ‘inhumane’ (YouGov, 2023). In contrast, comparable data from the United Kingdom showed that less than 1 in 10 British respondents considered prison conditions ‘too harsh’ (YouGov, 2024). Thus, US participants might find information about incarceration experiences more insightful, and they implicitly acknowledged the benefits of good prison conditions in our final study. The implication is that efforts aimed at addressing gatekeepers' reservations to hire formerly incarcerated persons should consider the target's receptiveness towards culturally relevant information, for example, emphasize the role of transformative experiences in culturally salient domains to humanize formerly incarcerated persons.

### Limitations and future directions

Finally, while we offer potential explanations for the mixed findings based on cultural differences, these are post‐hoc rationalizations, and further research is required to substantiate such ideas. Moreover, the implications of our findings must also be interpreted in light of several methodological limitations. It was hypothesized that dysphoric experiences are particularly potent signals of having undergone a personally transformative event (Newson et al., 2016, 2021; Whitehouse, 2018), but we found no differences between dysphoric and non‐dysphoric experience cues. It is possible that the potency of dysphoric experiences was limited by participants' preexisting levels of empathy for the target or that the choice of context did not elicit strong enough reactions or memories. It is plausible that significant effects could have been found using more emotionally laden dysphoric scenarios, in combination with targeted recruitment of a more closely defined social group (e.g. for group loss—referring to the impact of a terror attack with a sample of people from the affected area, e.g. see Jong et al., 2015; sports defeat—referring to a specific sporting event with a sample of fans from an impacted team). Relatedly, while we assumed that the chosen experiences (bereavement, natural disaster, sports defeat) were at least implicitly shared within a pool of recruited participants (S1‐3), we did not confirm whether participants had a relevant type of experience, something future studies could address with additional screening questions. Without a relevant reference experience, participants could have expressed how transformative they imagined such an event to be, which could have undermined the potential impact of relating to an experience cue. Future studies could also further contrast dysphoric experience cues with more clearly defined matched alternatives. The findings that learning about experiences in good (rather than bad) prison conditions facilitated fusion for Americans invites possible distinctions between constructive and destructive dysphoric experiences in future studies. Similarly, future research should consider the role of euphoric experience cues shared universally (e.g. milestones or achievements) or in domain contexts (e.g. group success). We also note that we have not contrasted this experience pathway with other proposed pathways to fusion, such as shared biology.

In the absence of experimental effects and interactions in most studies, we rely mainly on cross‐sectional associations to interpret our findings and thus, causal conclusions are limited. Moreover, our studies were by no means representative of their relative countries or target groups. For example, our sport fan samples were heavily biased towards male participants due to natural sports fan populations, and our study touching on transformative disaster experiences did not include participants born after 1995 so that they were more likely to have memories of Hurricane Katrina and contained few members of ethnic minority groups, who would have been more likely to be negatively impacted by such types of events (Smiley et al., 2012). Furthermore, our scenarios were entirely hypothetical. As much as we tried to improve ecological validity and include robust controls, more complex scenarios will be vital to better understand the effects and to test whether they can overcome direct and indirect biases against formerly incarcerated persons.

## CONCLUSION

Results suggest that personally transformative experiences are consistently powerful in tackling one of the greatest stigmas in modern society: having served time in prison. The interplay between gatekeepers' (i.e. potential employers') awareness of their own transformative experiences in a number of domains and their perceptions of a formerly incarcerated person's transformative experiences resulted in bonding to the applicant in both the United Kingdom and United States, which was linked to job offers and optimism that the applicant could desist from crime. These findings have obvious practical relevance for re‐inclusion efforts aimed at reducing recidivism.

## AUTHOR CONTRIBUTIONS

**Linus Peitz:** Conceptualization; methodology; data curation; formal analysis; writing – original draft; writing – review and editing. **Harvey Whitehouse:** Writing – original draft; writing – review and editing; funding acquisition. **Martha Newson:** Conceptualization; methodology; funding acquisition; writing – original draft; writing – review and editing.

## CONFLICT OF INTEREST

None of the authors have a conflict of interest to disclose.

## Supporting information


Appendix S1.

Appendix S2.

## Data Availability

The data that support the findings of this study are openly available via the Open Science Framework at https://osf.io/8vfu5/?view_only=e00bce25ee204219949a3a8010290242.

## Footnotes

^1^
We acknowledge that participants were not given the option to indicate having no reference experience at all. As a result, some participants may have provided ratings based on imagined or hypothetical transformation. While this cannot be empirically disproven, the significant correlations between transformativeness ratings and key outcomes suggest that even imagined experiences were meaningfully associated with fusion. Additional analyses of response patterns were conducted and showed no detectable random or repetitive responding to this measure (or its versions in Studies 2 and 3), and no concerns were raised by participants regarding inadequate response options.

^2^
These associations were previously reported in Newson et al. (2024).

^3^
Previously reported in Newson et al. (2024).

^4^
Previously reported in Newson et al. (2024).

^5^
Previously reported in Newson et al. (2024).
