# Supplementary material for: Can transformative experiences bridge the gap between receiving communities and formerly incarcerated persons?
Source: Br J Soc Psychol. 2025 May 6;64(3):e12886. doi: 10.1111/bjso.12886 (PMC12053958; doi:10.1111/bjso.12886)
Supplement: Supplementary file 1 — Appendix S1. [file BJSO-64-0-s002.docx]

Supplementary Information 1

Contents

[Supplementary Information A: Results from Studies 1a (US) & 1b (UK) 2](#_Toc156309506)

[Supplementary Information B: Results from Study 2 (US) 8](#_Toc156309507)

[Supplementary Information C: Results from Studies 3a (US) & 3b (UK) 11](#_Toc156309508)

[Supplementary Information D: Results from Studies 4a (US) & 4b (UK) 23](#_Toc156309509)

# Supplementary Information A: Studies 1a (US) & 1b (UK)

**Study details**

For Study 1a, 330 American citizens were recruited online. Ten participants were excluded because they failed the attention check. The final sample consisted of 320 participants (M_age_ = 44.84, SD_age_ 13.06), the majority of whom were male (56.6%; 42.5% female, 0.6% non-binary/ third gender, 0.3% undisclosed), and white (82.5%; 6.3% Asian, 5% Black, 3.1% Hispanic, 1.6% Mixed, 1.3% Other, 0.3% undisclosed). Ethical approval for this study was granted by the ethics board of the School of Anthropology and Conservation at the University of Kent : Ethics ID: 20231678778665296.

For Study 1b, a sample of 335 British citizens was recruited. Eight participants were excluded because they did not pass an attention check. The final sample consisted of 327 participants, (M_age_ = 42.09, SD_age_ = 11.17), the majority of whom were male (54.7%; 44.6% female, 0.6% undisclosed), and white (92%; 4.9% Asian, 1.8% Mixed, 0.6% Black, 0.6% undisclosed). Ethical approval for this study was granted by the ethics board of the School of Anthropology and Conservation at the University of Kent: Ethics ID: 20231674482788242.

Deviations from preregistration

For the analyses of experimental effects on identity fusion, dummy-coded regression was used instead of ANOVA to ensure methodological consistency and coherence in presenting results across multiple steps of analyses. The corresponding effects are presented as model 1 in Tables A1.2 and A2.2.

| **Table A1.1**  *Cronbach Alphas, Descriptive Statistics and Correlations of Variables in Study 1a (US)* | | | | | | | | | | | | | | | | | |
| --- | --- | --- | --- | --- | --- | --- | --- | --- | --- | --- | --- | --- | --- | --- | --- | --- | --- |
| Variables | α | M | (SD) | 2. | 3. | 4. | 5. | 6. | 7. | 8. | 9. | 10. | 11. | 12. | 13. | 14. | 15. |
| 1. Willingness to hire |  | 4.06 | 1.23 | .09 | -.03 | .53*** | .14* | -.09 | -.07 | -.04 | -.11* | -.02 | .11 | -.19*** | .33*** | .15** | .11* |
| 1. Direct experience |  | 0.33 | 0.47 |  | -.50*** | .15** | .08 | -.02 | -.05 | -.02 | -.01 | -.03 | .12* | -.09 | -.01 | .10 | .18** |
| 1. Indirect experience |  | 0.33 | 0.47 |  |  | -.09 | -.13* | .05 | .04 | .03 | .07 | -.01 | -.03 | .08 | -.06 | -.15** | -.14* |
| 1. Identity fusion | .88 | 2.67 | 1.30 |  |  |  | .19*** | -.05 | -.06 | -.05 | -.05 | -.10 | .04 | -.15** | .21*** | .09 | .09 |
| 1. Experience transformativeness | .88 | 5.48 | 1.33 |  |  |  |  | .12* | -.21*** | .06 | .03 | .04 | .03 | -.10 | .16** | .11* | .08 |
| 1. Age |  | 44.84 | 13.06 |  |  |  |  |  | -.08 | .21*** | -.01 | .03 | .25*** | .09 | -.03 | .00 | .02 |
| 1. Gender |  | 0.57 | 0.50 |  |  |  |  |  |  | -.11 | .14* | .00 | .08 | .20*** | -.02 | -.00 | -.03 |
| 1. Ethnicity |  | 0.83 | 0.38 |  |  |  |  |  |  |  | -.08 | -.09 | .10 | .02 | -.02 | -.03 | -.11 |
| 1. SES |  | 5.33 | 1.73 |  |  |  |  |  |  |  |  | .47*** | .12* | .06 | -.08 | -.17*** | -.10 |
| 1. Education |  | 3.71 | 1.01 |  |  |  |  |  |  |  |  |  | .06 | -.05 | -.14* | -.18*** | -.07 |
| 1. Hiring experience |  | 2.25 | 1.65 |  |  |  |  |  |  |  |  |  |  | .01 | .09 | .16** | .08 |
| 1. Political ideology | .96 | 4.09 | 2.96 |  |  |  |  |  |  |  |  |  |  |  | -.08 | .04 | -.04 |
| 1. Open-mindedness | .96 | 3.96 | 1.08 |  |  |  |  |  |  |  |  |  |  |  |  | .15** | .05 |
| 1. Contact ex-prisoners |  | 0.86 | 0.76 |  |  |  |  |  |  |  |  |  |  |  |  |  | .33*** |
| 1. Victim of crime |  | 0.60 | 0.49 |  |  |  |  |  |  |  |  |  |  |  |  |  |  |
| *Note.* Gender 1 = Male, Ethnicity 1 = White, Victim status 1 = Yes  **p <* .05, ***p* < .01, ****p* <.001 | | | | | | | | | | | | | | | | | |

| **Table A1.2**  *Multiple Regression Analyses Predicting Identity Fusion to the Ex-Prisoner in Study 1a (US)* | | | | | | | | | | | | | | | | |
| --- | --- | --- | --- | --- | --- | --- | --- | --- | --- | --- | --- | --- | --- | --- | --- | --- |
| Variables | Model 1 | | | | Model 2 | | | | Model 3 | | | | Model 4 | | | |
|  | B | SE | *β* | *p* | B | SE | *β* | *p* | B | SE | *β* | *p* | B | SE | *β* | *p* |
| Dummy I: Direct experience | 0.38 | 0.18 | .14 | .031 | 0.37 | 0.17 | .14 | .032 | 0.38 | 0.17 | .14 | .026 | 0.93 | 0.79 | .34 | .240 |
| Dummy II: Indirect experience | -0.05 | 0.18 | -.02 | .781 | 0.02 | 0.18 | .01 | .934 | 0.06 | 0.17 | .02 | .733 | 0.40 | 0.70 | .15 | .567 |
| Experience transformativeness | - | - | - | - | 0.18 | 0.05 | .18 | .001 | 0.14 | 0.05 | .15 | .009 | 0.19 | 0.09 | .20 | .038 |
| Open-mindedness | - | - | - | - | - | - | - | - | 0.21 | 0.07 | .18 | .001 | 0.22 | 0.07 | .18 | .001 |
| Political ideology | - | - | - | - | - | - | - | - | -0.05 | 0.02 | -.11 | .036 | -0.05 | 0.02 | -.12 | .035 |
| Dummy I X Experience transformativeness | - | - | - | - | - | - | - | - | - | - | - | - | -0.10 | 0.14 | -.21 | .476 |
| Dummy II X Experience transformativeness | - | - | - | - | - | - | - | - | - | - | - | - | -0.06 | 0.12 | -.12 | .621 |
| Model Summary | *F*(2,317) = 3.57, *p* = .029, R^2^ = .02 | | | | *F*(3, 316) = 6.10, *p* < .001, R^2^ = .06 | | | | *F*(5, 314) = 7.06, *p* < .001, R^2^ = .10 | | | | *F*(7, 312) = 5.09, *p* < .001, R^2^ = .10 | | | |

| **Table A1.3**  *Multiple Regression Analyses Predicting Willingness to Hire the Ex-Prisoner in Study 1a (US)* | | | | | | | | | | | | |
| --- | --- | --- | --- | --- | --- | --- | --- | --- | --- | --- | --- | --- |
| Variables | Model 1 | | | | Model 2 | | | | Model 3 | | | |
|  | B | SE | *β* | *p* | B | SE | *β* | *p* | B | SE | *β* | *p* |
| Dummy I: direct experience | 0.26 | 0.17 | .10 | .120 | 0.07 | 0.14 | .03 | .621 | 0.10 | 0.14 | .04 | .494 |
| Dummy II: indirect experience | 0.07 | 0.17 | .03 | .697 | 0.09 | 0.14 | .04 | .531 | 0.19 | 0.14 | .07 | .176 |
| Identity fusion | - | - | - | - | 0.50 | 0.05 | .53 | .000 | 0.43 | 0.05 | .46 | .000 |
| Experience transformativeness | - | - | - | - | - | - | - | - | 0.00 | 0.04 | .00 | .958 |
| Open-mindedness | - | - | - | - | - | - | - | - | 0.24 | 0.05 | .21 | .000 |
| Political ideology | - | - | - | - | - | - | - | - | -0.04 | 0.02 | -.10 | .036 |
| Contact ex-prisoner | - | - | - | - | - | - | - | - | 0.11 | 0.08 | .07 | .198 |
| Victim status | - | - | - | - | - | - | - | - | 0.08 | 0.12 | .03 | .512 |
| SES | - | - | - | - | - | - | - | - | -0.04 | 0.03 | -.06 | .233 |
| Model Summary | *F*(2,317) = 1.31, *p* = .271, R^2^ = .01 | | | | *F*(3, 316) = 40.92, *p* < .001, R^2^ = .28 | | | | *F*(9, 310) = 18.67, *p* < .001, R^2^ = .35 | | | |
|  |  | | | |  | | | |  | | | |
| Dummy I: Indirect effect via fusion |  |  |  |  |  |  |  |  | B = 0.16, SE = 0.08, 95%CI [.012, .320] | | | |
| Dummy I: Total effect |  |  |  |  |  |  |  |  | B = 0.25, SE = 0.16, 95%CI [-.057, .564] | | | |
| Dummy II: Indirect effect via fusion |  |  |  |  |  |  |  |  | B = 0.03, SE = 0.07, 95%CI [-.116, .186] | | | |
| Dummy II: Total effect |  |  |  |  |  |  |  |  | B = 0.22, SE = 0.16, 95%CI [-.089, .535] | | | |
| *Note.* Victim status 1 = Yes | | | | | | | | | | | | |

| **Table A2.1**  *Cronbach Alphas, Descriptive Statistics and Correlations of Variables in Study 1b (UK)* | | | | | | | | | | | | | | | | | |
| --- | --- | --- | --- | --- | --- | --- | --- | --- | --- | --- | --- | --- | --- | --- | --- | --- | --- |
| Variables | α | M | (SD) | 2. | 3. | 4. | 5. | 6. | 7. | 8. | 9. | 10. | 11. | 12. | 13. | 14. | 15. |
| 1. Willingness to hire |  | 4.30 | 1.03 | .12* | -.10 | .41*** | .15** | -.15** | -.04 | .08 | -.14* | -.01 | .01 | -.19*** | .33*** | .05 | .05 |
| 1. Direct experience |  | 0.34 | 0.47 |  | -.51*** | .00 | -.06 | -.08 | -.02 | -.03 | -.03 | .02 | .07 | -.00 | -.02 | -.06 | .03 |
| 1. Indirect experience |  | 0.34 | 0.48 |  |  | .07 | .02 | .05 | -.00 | -.05 | .12* | .07 | -.04 | .08 | -.02 | -.01 | .05 |
| 1. Identity fusion | .85 | 3.07 | 1.19 |  |  |  | .16** | -.03 | .10 | -.01 | -.09 | .02 | -.10 | -.07 | .28*** | .12* | -.03 |
| 1. Experience transformativeness | .83 | 5.35 | 1.22 |  |  |  |  | .13* | -.14** | -.01 | -.02 | .07 | -.13* | .04 | .02 | .04 | .05 |
| 1. Age |  | 42.09 | 11.17 |  |  |  |  |  | .05 | .16** | .24*** | -.01 | .23*** | .21*** | -.10 | .10 | .11 |
| 1. Gender |  | 0.55 | 0.50 |  |  |  |  |  |  | .03 | .07 | -.01 | .03 | .08 | .07 | .11 | .03 |
| 1. Ethnicity |  | 0.92 | 0.27 |  |  |  |  |  |  |  | .07 | -.03 | .02 | .01 | -.03 | .12* | .06 |
| 1. SES |  | 5.75 | 1.43 |  |  |  |  |  |  |  |  | .30*** | .17** | .13* | -.04 | -.08 | -.05 |
| 1. Education |  | 3.92 | 0.85 |  |  |  |  |  |  |  |  |  | -.01 | -.08 | -.01 | -.03 | .01 |
| 1. Hiring experience |  | 2.04 | 1.57 |  |  |  |  |  |  |  |  |  |  | .15** | .02 | .10 | .11 |
| 1. Political ideology | .93 | 3.97 | 2.07 |  |  |  |  |  |  |  |  |  |  |  | -.21*** | .04 | -.02 |
| 1. Open-mindedness | .93 | 3.87 | 0.91 |  |  |  |  |  |  |  |  |  |  |  |  | .01 | -.02 |
| 1. Contact ex-prisoners |  | 0.60 | 0.61 |  |  |  |  |  |  |  |  |  |  |  |  |  | .06 |
| 1. Victim status |  | 0.71 | 0.46 |  |  |  |  |  |  |  |  |  |  |  |  |  |  |
| *Note.* Gender 1 = Male, Ethnicity 1 = White, Victim status 1 = Yes  **p <* .05, ***p* < .01, ****p* <.001 | | | | | | | | | | | | | | | | | |

| **Table A2.2**  *Multiple Regression Analyses Predicting Identity Fusion to the Ex-Prisoner in Study 1b (UK)* | | | | | | | | | | | | | | | | |
| --- | --- | --- | --- | --- | --- | --- | --- | --- | --- | --- | --- | --- | --- | --- | --- | --- |
| Variables | Model 1 | | | | Model 2 | | | | Model 3 | | | | Model 4 | | | |
|  | B | SE | *β* | *p* | B | SE | *β* | *p* | B | SE | *β* | *p* | B | SE | *β* | *p* |
| Dummy I: direct bereavement | 0.13 | 0.16 | .05 | .440 | 0.15 | 0.16 | .06 | .342 | 0.20 | 0.15 | .08 | .189 | -0.22 | 0.67 | -.09 | .743 |
| Dummy II: indirect bereavement | 0.23 | 0.16 | .09 | .160 | 0.23 | 0.16 | .09 | .145 | 0.28 | 0.15 | .11 | .072 | -0.46 | 0.72 | -.19 | .521 |
| Experience transformativeness |  |  |  |  | 0.16 | 0.05 | .16 | .003 | 0.15 | 0.05 | .15 | .004 | 0.08 | 0.09 | .08 | .382 |
| Open-mindedness |  |  |  |  |  |  |  |  | 0.36 | 0.07 | .28 | .000 | 0.37 | 0.07 | .28 | .000 |
| Contact ex-prisoners |  |  |  |  |  |  |  |  | 0.22 | 0.10 | .11 | .031 | 0.22 | 0.10 | .11 | .034 |
| Dummy I X Exp transformativeness |  |  |  |  |  |  |  |  |  |  |  |  | 0.08 | 0.12 | .17 | .522 |
| Dummy II X Exp transformativeness |  |  |  |  |  |  |  |  |  |  |  |  | 0.14 | 0.13 | .30 | .295 |
| Model Summary | *F*(2,324) = 0.99, *p* = .372, R^2^ = .01 | | | | *F*(3, 323) = 3.60, *p* = .014, R^2^ = .03 | | | | *F*(5, 321) = 8.96, *p* < .001, R^2^ = .12 | | | | *F*(7, 319) = 6.55, *p* < .001, R^2^ = .13 | | | |

| **Table A2.3**  *Multiple Regression Analyses Predicting Willingness to Hire the Ex-Prisoner in Study 1b (UK)* | | | | | | | | | | | | |
| --- | --- | --- | --- | --- | --- | --- | --- | --- | --- | --- | --- | --- |
| Variables | Model 1 | | | | Model 2 | | | | Model 3 | | | |
|  | B | SE | *β* | *p* | B | SE | *β* | *p* | B | SE | *β* | *p* |
| Dummy I: direct bereavement | 0.21 | 0.14 | .10 | .140 | 0.16 | 0.13 | .07 | .208 | 0.21 | 0.12 | .10 | .090 |
| Dummy II: indirect bereavement | -0.11 | 0.14 | -.05 | .451 | -0.19 | 0.13 | -.09 | .143 | -0.10 | 0.12 | -.05 | .399 |
| Identity fusion |  |  |  |  | 0.36 | 0.04 | .41 | .000 | 0.28 | 0.04 | .32 | .000 |
| Experience transformativeness |  |  |  |  |  |  |  |  | 0.10 | 0.04 | .12 | .015 |
| Open-mindedness |  |  |  |  |  |  |  |  | 0.24 | 0.06 | .21 | .000 |
| Political ideology |  |  |  |  |  |  |  |  | -0.05 | 0.03 | -.10 | .052 |
| Age |  |  |  |  |  |  |  |  | -0.01 | 0.01 | -.09 | .074 |
| SES |  |  |  |  |  |  |  |  | -0.04 | 0.04 | -.06 | .264 |
|  |  |  |  |  |  |  |  |  |  |  |  |  |
| Dummy I: Indirect effect via fusion |  |  |  |  |  |  |  |  | B = 0.05, SE = 0.04, 95%CI [-.027, .137] | | | |
| Dummy I: Total effect |  |  |  |  |  |  |  |  | B = 0.26, SE = 0.13, 95%CI [.006, .51] | | | |
| Dummy II: Indirect effect via fusion |  |  |  |  |  |  |  |  | B = 0.08, SE = 0.05, 95%CI [-.003, .176] | | | |
| Dummy II: Total effect |  |  |  |  |  |  |  |  | B = -0.02, SE = 0.13, 95%CI [-.277, .232] | | | |
| Model Summary | *F*(2,324) = 2.65, *p* = .072, R^2^ = .02 | | | | *F*(3, 323) = 24.46, *p* <.001, R^2^ = .19 | | | | *F*(8, 318) = 15.00, *p* <.001, R^2^ = .27 | | | |

# Supplementary Information B: Study 2 (US)

**Study details**

333 US citizens were recruited online. Given the content of the experimental manipulation, we pre-screened the sample to include only US nationals who were at least 10 years old when Hurricane Katrina happened. 14 participants were excluded because they failed an attention check. The final sample consisted of 319 participants, (M_age_ = 42.49, SD_age_ 11.69), the majority of whom were female (57.7%; 41.4% male, 0.6% non-binary, 0.3% prefer not to say), and white (74%; 7.8% Black, 6.3% Hispanic, 5% Asian, 3.4% Mixed, 2.5% Other, 0.9% Prefer not to say). Ethical approval for this study was granted by the ethics board of the School of Anthropology and Conservation at the University of Kent: Ethics ID: 20231680606154343.

| **Table B1**  *Cronbach Alphas, Descriptive Statistics and Correlations of Variables in Study 2 (US)* | | | | | | | | | | | | | | | | | | | | |
| --- | --- | --- | --- | --- | --- | --- | --- | --- | --- | --- | --- | --- | --- | --- | --- | --- | --- | --- | --- | --- |
| Variables | α | M | (SD) | 2. | 3. | 4. | 5. | 6. | 7. | 10. | 11. | 12. | 13. | 14. | 15. | 16. | 17. | 18. | 19. | 20. |
| 1. Willingness to hire |  | 4.65 | 1.03 | .65*** | .30*** | .09 | -.04 | .11 | -.09 | -.13* | -.06 | .06 | -.13* | -.11 | -.03 | -.20*** | .28*** | .22*** | .11* | -.04 |
| 1. Perceived chances to desist |  | 3.57 | .81 |  | .33*** | .13* | -.06 | .16** | .04 | -.03 | -.04 | -.05 | -.01 | -.05 | -.01 | -.13* | .24*** | .12* | -.00 | .05 |
| 1. Identity fusion | .91 | 2.06 | .95 |  |  | .09 | -.02 | .26*** | .16** | -.06 | .15** | -.09 | .09 | .03 | .12* | -.03 | .12* | .11* | -.05 | .17** |
| 1. Dysphoric experience |  | .34 | .47 |  |  |  | -.50*** | .02 | .05 | .04 | .00 | -.15** | .07 | .01 | .00 | .06 | .03 | .01 | .05 | .08 |
| 1. Non-dysphoric experience |  | .33 | .47 |  |  |  |  | .05 | -.05 | -.04 | -.01 | .04 | -.01 | .04 | .06 | -.12* | .01 | .03 | .08 | -.07 |
| 1. Experience transformativeness | .87 | 3.65 | .93 |  |  |  |  |  | .32*** | .16** | -.15* | -.01 | .11 | .08 | .18** | .08 | .09 | .18** | -.07 | .16** |
| 1. Symbolic patriotism |  | 2.82 | 1.37 |  |  |  |  |  |  | .37*** | .01 | .05 | .16** | -.03 | .17** | .57*** | -.06 | .00 | -.11* | .18** |
| 1. Age |  | 42.49 | 11.69 |  |  |  |  |  |  |  | -.17** | .22*** | .03 | .07 | .18** | .30*** | -.05 | .02 | -.06 | .11 |
| 1. Gender |  | .41 | .49 |  |  |  |  |  |  |  |  | -.05 | .04 | .01 | .08 | .08 | .04 | .01 | -.00 | .01 |
| 1. Ethnicity |  | .74 | .44 |  |  |  |  |  |  |  |  |  | .08 | -.01 | .09 | .07 | -.04 | .01 | .07 | -.12* |
| 1. SES |  | 4.92 | 1.64 |  |  |  |  |  |  |  |  |  |  | .38*** | .15** | .04 | -.13* | -.16** | -.14* | .12* |
| 1. Education |  | 3.42 | 1.08 |  |  |  |  |  |  |  |  |  |  |  | .19*** | -.12* | -.04 | -.08 | -.09 | .04 |
| 1. Hiring experience |  | 1.51 | 1.69 |  |  |  |  |  |  |  |  |  |  |  |  | .09 | -.13* | .14* | .07 | .06 |
| 1. Ideology | .97 | 3.64 | 2.96 |  |  |  |  |  |  |  |  |  |  |  |  |  | -.18*** | .06 | -.14* | .08 |
| 1. Open mindedness | .95 | 3.46 | .95 |  |  |  |  |  |  |  |  |  |  |  |  |  |  | .16* | .10 | -.02 |
| 1. Contact ex-prisoner |  | 1.79 | .69 |  |  |  |  |  |  |  |  |  |  |  |  |  |  |  | .22*** | -.10 |
| 1. Victim status |  | .65 | .48 |  |  |  |  |  |  |  |  |  |  |  |  |  |  |  |  | -.13** |
| 1. Social desirability | .82 | 8.74 | 3.92 |  |  |  |  |  |  |  |  |  |  |  |  |  |  |  |  |  |
| *Note.* Gender 1= Male, Ethnicity 1 = White, Victim status 1 = Yes  **p <* .05, ***p* < .01, ****p* <.001 | | | | | | | | | | | | | | | | | | | | |

| **Table B2**  *Multiple Regression Analyses Predicting Identity Fusion to the Ex-Prisoner (S2)* | | | | | | | | | | | | | | | | | | | | |
| --- | --- | --- | --- | --- | --- | --- | --- | --- | --- | --- | --- | --- | --- | --- | --- | --- | --- | --- | --- | --- |
| Variables | Model 1 | | | | Model 2 | | | | Model 3 | | | | Model 4 | | | | Model 5 | | | |
|  | B | SE | *β* | *p* | B | SE | *β* | *p* | B | SE | *β* | *p* | B | SE | *β* | *p* | B | SE | *β* | *p* |
| Dummy I: dysphoric experience | 0.21 | 0.13 | .11 | .105 | 0.17 | 0.13 | .09 | .167 | 0.15 | 0.12 | .07 | .228 | 0.86 | 0.50 | .43 | .088 | 0.22 | 0.29 | .11 | .442 |
| Dummy II: non-dysphoric experience | 0.07 | 0.13 | .03 | .612 | 0.03 | 0.13 | .02 | .805 | 0.03 | 0.12 | .01 | .822 | -0.06 | 0.48 | -.03 | .899 | -0.16 | 0.29 | -.08 | .572 |
| Experience transformativeness |  |  |  |  | 0.23 | 0.06 | .23 | <.001 | 0.22 | 0.06 | .21 | <.001 | 0.26 | 0.09 | .26 | .004 | 0.22 | 0.06 | .22 | <.001 |
| Symbolic patriotism |  |  |  |  | 0.06 | 0.04 | .09 | .120 | 0.05 | 0.04 | .07 | .221 | 0.05 | 0.04 | .08 | .175 | 0.03 | 0.07 | .05 | .617 |
| Open-mindedness |  |  |  |  |  |  |  |  | 0.09 | 0.05 | .09 | .091 | 0.09 | 0.05 | .09 | .087 | 0.09 | 0.06 | .09 | .097 |
| Contact ex-prisoners |  |  |  |  |  |  |  |  | 0.08 | 0.08 | .06 | .274 | 0.08 | 0.08 | .06 | .288 | 0.08 | 0.08 | .06 | .304 |
| Gender |  |  |  |  |  |  |  |  | 0.33 | 0.10 | .17 | .002 | 0.34 | 0.10 | .18 | .001 | 0.33 | 0.10 | .17 | .002 |
| Hiring experience |  |  |  |  |  |  |  |  | 0.03 | 0.03 | .05 | .358 | 0.03 | 0.03 | .05 | .389 | 0.03 | 0.03 | .04 | .429 |
| Social desirability |  |  |  |  |  |  |  |  | 0.03 | 0.01 | .12 | .033 | 0.03 | 0.01 | .11 | .038 | 0.03 | 0.01 | .11 | .042 |
| Dummy I X Exp transformativeness |  |  |  |  |  |  |  |  |  |  |  |  | -0.20 | 0.14 | -.37 | .148 |  |  |  |  |
| Dummy II X Exp transformativeness |  |  |  |  |  |  |  |  |  |  |  |  | 0.02 | 0.13 | .04 | .864 |  |  |  |  |
| Dummy I X Football fandom |  |  |  |  |  |  |  |  |  |  |  |  |  |  |  |  | -0.02 | 0.09 | -.04 | .789 |
| Dummy II X Football fandom |  |  |  |  |  |  |  |  |  |  |  |  |  |  |  |  | 0.07 | 0.09 | .11 | .458 |
| Model Summary | *F*(2,316) = 1.39, *p* = .250, R^2^ = .01 | | | | *F*(4, 314) = 6.87, *p* <.001, R^2^ = .08 | | | | *F*(9, 309) = 5.60, *p* < .001, R^2^ = .14 | | | | *F*(11, 307) = 4.68, *p* <. 001, R^2^ = .14 | | | | *F*(11, 307) = 3.74, *p* < .001, R^2^ = .14 | | | |
| *Note.* Gender 1 = Male | | | | | | | | | | | | | | | | | | | | |

| **Table B3**  *Multiple Linear Regression predicting Willingness to Hire Ex-Prisoner Based on Experimental Conditions (Model 1), Identity Fusion (Model 2) and Experience transformativeness, Symbolic Patriotism, Identity Fusion and Relevant Control Variables (Model 3)* | | | | | | | | | | | | |
| --- | --- | --- | --- | --- | --- | --- | --- | --- | --- | --- | --- | --- |
| Variables | Model 1 | | | | Model 2 | | | | Model 3 | | | |
|  | B | SE | *β* | *p* | B | SE | *β* | *p* | B | SE | *β* | *p* |
| Dummy I: Dysphoric experience | 0.22 | 0.14 | .10 | .128 | 0.16 | 0.14 | .07 | .274 | 0.14 | 0.13 | .07 | .275 |
| Dummy II: Non-dysphoric experience | 0.02 | 0.14 | .01 | .872 | 0.00 | 0.14 | .00 | .988 | -0.07 | 0.13 | -.03 | .571 |
| Identity fusion |  |  |  |  | 0.32 | 0.06 | .29 | .000 | 0.27 | 0.06 | .25 | .000 |
| Experience transformativeness |  |  |  |  |  |  |  |  | 0.05 | 0.06 | .05 | .424 |
| Symbolic patriotism |  |  |  |  |  |  |  |  | -0.01 | 0.05 | -.01 | .835 |
| Open-mindedness |  |  |  |  |  |  |  |  | 0.19 | 0.06 | .18 | .001 |
| Political ideology |  |  |  |  |  |  |  |  | -0.05 | 0.02 | -.14 | .031 |
| Contact ex-prisoners |  |  |  |  |  |  |  |  | 0.20 | 0.08 | .14 | .014 |
| Victim status |  |  |  |  |  |  |  |  | 0.09 | 0.12 | .04 | .455 |
| Age |  |  |  |  |  |  |  |  | -0.01 | 0.01 | -.07 | .236 |
| SES |  |  |  |  |  |  |  |  | -0.07 | 0.03 | -.10 | .051 |
| Model Summary | *F*(2,316) = 1.42, *p* = .244, R^2^ = .01 | | | | *F*(3, 315) = 10.66, *p* < .001, R^2^ = .09 | | | | *F*(11, 307) = 7.92, *p* < .001, R^2^ = .22 | | | |
| *Note.* Victim status 1 = Yes | | | | | | | | | | | | |

| **Table B4**  *Multiple Linear Regression predicting Perceived Future Chances of the Ex-Prisoner Based on Experimental Conditions (Model 1), Identity Fusion (Model 2) and Experience transformativeness, Symbolic Patriotism, Identity Fusion and Relevant Control Variables (Model 3)* | | | | | | | | | | | | |
| --- | --- | --- | --- | --- | --- | --- | --- | --- | --- | --- | --- | --- |
| Variables | Model 1 | | | | Model 2 | | | | Model 3 | | | |
|  | B | SE | *β* | *p* | B | SE | *β* | *p* | B | SE | *β* | *p* |
| Dummy I: Dysphoric experience | 0.24 | 0.11 | .14 | .032 | 0.18 | 0.10 | .11 | .088 | 0.16 | 0.10 | .10 | .110 |
| Dummy II: Non-dysphoric experience | 0.02 | 0.11 | .01 | .830 | 0.01 | 0.10 | .00 | .958 | -0.04 | 0.10 | -.02 | .698 |
| Identity fusion |  |  |  |  | 0.28 | 0.05 | .33 | .000 | 0.23 | 0.05 | .27 | .000 |
| Experience transformativeness |  |  |  |  |  |  |  |  | 0.05 | 0.05 | .06 | .299 |
| Symbolic patriotism |  |  |  |  |  |  |  |  | 0.04 | 0.04 | .06 | .343 |
| Open-mindedness |  |  |  |  |  |  |  |  | 0.14 | 0.05 | .17 | .002 |
| Political ideology |  |  |  |  |  |  |  |  | -0.04 | 0.02 | -.15 | .027 |
| Contact ex-prisoner |  |  |  |  |  |  |  |  | 0.07 | 0.06 | .06 | .261 |
| Model Summary | *F*(2,316) = 2.84, *p* = .060, R^2^ = .02 | | | | F(3, 315)=14.62, *p* <.001, R^2^=.12 | | | | *F*(11, 307) = 8.70, *p* < .001, R^2^ = .18 | | | |

# Supplementary Information C: Studies 3a (US) & 3b (UK)

**Study details**

For S3a, 330 US citizens with an interest in American football were recruited online. 36 participants were excluded because they did not pass an attention check. The final sample consisted of 294 participants, (M_age_ = 40.92, SD_age_ 12.47), the majority of whom were male (96.9%; 2.4% female, 0.3% non-binary/third gender, 0.3% undisclosed, and white (76.2%; 7.1 % Black, 7.1% Hispanic, 4.1% Mixed, 3.7% Asian, 1.4% Other, 0.3% undisclosed). Ethical approval for this study was granted by the ethics board of the School of Anthropology and Conservation at the University of Kent: Ethics ID: 20231679036795309.

For S3b, 331 British citizens with an interest in soccer were recruited. 28 participants were excluded because they did not pass an attention check. The final sample consisted of 303 participants, (M_age_ = 41.12, SD_age_ 14.25), the majority of whom were male (80.5%; 18.8% female; 0.7% undisclosed), and white (82.5%; 9.2% Asian, 4% Mixed, 1.7% Black, 1% Other, 0.3% North African, 1.3% undisclosed). Ethical approval for this study was granted by the ethics board of the School of Anthropology and Conservation at the University of Kent: Ethics ID: 20231675692193254.

Deviations from preregistration

For the analyses of experimental effects on identity fusion, dummy-coded regression was used instead of ANOVA to ensure methodological consistency and coherence in presenting results across multiple steps of analyses. The corresponding effects are presented as model 1 in Tables C1.2 and C2.2.

| **Table C1.1**  *Cronbach Alphas, Descriptive Statistics and Correlations of Variables in Study 3a (US)* | | | | | | | | | | | | | | | | | | | | |
| --- | --- | --- | --- | --- | --- | --- | --- | --- | --- | --- | --- | --- | --- | --- | --- | --- | --- | --- | --- | --- |
| Variables | α | M | (SD) | 2. | 3. | 4. | 5. | 6. | 7. | 8. | 9. | 10. | 11. | 12. | 13. | 14. | 15. | 16. | 17. | 18. |
| 1. Willingness to hire |  | 4.60 | 1.00 | .57*** | .34*** | -.01 | .01 | .07 | .09 | -.16** | -.03 | -.07 | -.03 | -.09 | .11 | -.22*** | .20*** | .24*** | .16** | -.03 |
| 1. Perceived future chances |  | 3.56 | 0.84 |  | .37*** | .01 | -.03 | -.01 | .01 | -.01 | .00 | -.11 | .03 | -.12* | .10 | .00 | .19** | .15** | .03 | -.04 |
| 1. Identity fusion | .88 | 2.18 | 0.89 |  |  | .02 | .00 | .11 | .10 | .06 | -.12* | -.15 | .07 | -.05 | .04 | -.06 | .04 | .25*** | -.02 | .13 |
| 1. Dysphoric experience |  | 0.34 | 0.47 |  |  |  | -.46*** | .03 | -.08 | -.02 | .04 | .01 | .07 | .03 | .01 | -.02 | -.06 | -.16** | .01 | .08 |
| 1. Non-dysphoric experience |  | 0.29 | 0.46 |  |  |  |  | .01 | .07 | -.03 | -.06 | -.10 | -.05 | -.01 | -.07 | -.02 | .02 | .04 | .01 | -.03 |
| 1. Experience transformativeness | .84 | 3.41 | 0.96 |  |  |  |  |  | .54*** | -.03 | .18** | .06 | .10 | .07 | .07 | -.01 | .23*** | .15* | .04 | .06 |
| 1. Sport fandom |  | 3.58 | 1.04 |  |  |  |  |  |  | .01 | .10 | -.00 | .15* | .04 | .05 | .02 | .22*** | .10 | -.01 | -.01 |
| 1. Age |  | 40.92 | 12.47 |  |  |  |  |  |  |  | -.03 | .14* | .12* | .08 | .45*** | .12* | -.02 | .09 | .06 | -.08 |
| 1. Gender |  | 0.97 | 0.17 |  |  |  |  |  |  |  |  | .09 | .05 | .04 | -.00 | -.05 | .06 | .02 | .02 | -.01 |
| 1. Ethnicity |  | 0.76 | 0.43 |  |  |  |  |  |  |  |  |  | .08 | .02 | .07 | .07 | .03 | -.08 | -.00 | -.05 |
| 1. SES |  | 5.37 | 1.62 |  |  |  |  |  |  |  |  |  |  | .37*** | .12* | .15* | .00 | -.08 | -.09 | .12* |
| 1. Education |  | 3.65 | 1.00 |  |  |  |  |  |  |  |  |  |  |  | .01 | -.08 | -.07 | -.04 | .04 | .03 |
| 1. Hiring experience |  | 2.09 | 1.81 |  |  |  |  |  |  |  |  |  |  |  |  | .12* | .08 | .07 | .13* | -.02 |
| 1. Ideology | .94 | 4.69 | 2.61 |  |  |  |  |  |  |  |  |  |  |  |  |  | -.18** | -.08 | -.13* | .05 |
| 1. Open-mindedness | .95 | 3.61 | 0.92 |  |  |  |  |  |  |  |  |  |  |  |  |  |  | .08 | -.05 | .10 |
| 1. Contact ex-prisoner |  | 1.86 | 0.69 |  |  |  |  |  |  |  |  |  |  |  |  |  |  |  | .35*** | -.10 |
| 1. Victim status |  | 0.61 | 0.49 |  |  |  |  |  |  |  |  |  |  |  |  |  |  |  |  | -.17** |
| 1. Social desirability | .82 | 9.20 | 3.93 |  |  |  |  |  |  |  |  |  |  |  |  |  |  |  |  |  |
| *Note.* Gender 1= Male, Ethnicity 1 = White, Victim status 1 = Yes  **p <* .05, ***p* < .01, ****p* <.001 | | | | | | | | | | | | | | | | | | | | |

| **Table C1.2**  *Multiple Regression Analyses Predicting Identity Fusion to the Ex-Prisoner in Study 3a (US)* | | | | | | | | | | | | | | | | | | | | |
| --- | --- | --- | --- | --- | --- | --- | --- | --- | --- | --- | --- | --- | --- | --- | --- | --- | --- | --- | --- | --- |
| Variables | Model 1 | | | | Model 2 | | | | Model 3 | | | | Model 4 | | | | Model 5 | | | |
|  | B | SE | *β* | *p* | B | SE | *β* | *p* | B | SE | *β* | *p* | B | SE | *β* | *p* | B | SE | *β* | *p* |
| Dummy I: dysphoric experience | 0.05 | 0.12 | .03 | .709 | 0.05 | 0.12 | .03 | .705 | 0.14 | 0.12 | .07 | .262 | -0.11 | 0.45 | -.06 | .813 | -0.17 | 0.44 | -.09 | .697 |
| Dummy II: non-dysphoric experience | 0.03 | 0.13 | .01 | .826 | 0.02 | 0.13 | .01 | .871 | 0.03 | 0.12 | .01 | .836 | -0.54 | 0.46 | -.28 | .237 | -0.42 | 0.44 | -.21 | .347 |
| Experience transformativeness |  |  |  |  | 0.07 | 0.07 | .08 | .255 | 0.06 | 0.06 | .07 | .316 | -0.00 | 0.09 | -.00 | .988 | 0.06 | 0.06 | .07 | .348 |
| Football fandom |  |  |  |  | 0.05 | 0.06 | .05 | .444 | 0.04 | 0.06 | .05 | .448 | 0.03 | 0.06 | .04 | .560 | -0.02 | 0.09 | -.03 | .806 |
| Contact ex-prisoners |  |  |  |  |  |  |  |  | 0.33 | 0.08 | .25 | .000 | 0.33 | 0.08 | .25 | .000 | 0.33 | 0.08 | .26 | .000 |
| Gender |  |  |  |  |  |  |  |  | -0.74 | 0.30 | -.14 | .013 | -0.80 | 0.30 | -.16 | .008 | -0.77 | 0.30 | -.15 | .011 |
| Dummy I X Exp transformativeness |  |  |  |  |  |  |  |  |  |  |  |  | 0.07 | 0.13 | .14 | .574 |  |  |  |  |
| Dummy II X Exp transformativeness |  |  |  |  |  |  |  |  |  |  |  |  | 0.17 | 0.13 | .31 | .198 |  |  |  |  |
| Dummy I X Football fandom |  |  |  |  |  |  |  |  |  |  |  |  |  |  |  |  | 0.09 | 0.12 | .17 | .469 |
| Dummy II X Football fandom |  |  |  |  |  |  |  |  |  |  |  |  |  |  |  |  | 0.12 | 0.12 | .24 | .297 |
| Model Summary | *F*(2,291) = 0.07, *p* = .931, R^2^ = .00 | | | | *F*(4, 289) = 1.04, *p* = .386, R^2^ = .01 | | | | *F*(6, 287) = 4.97, *p* < .001, R^2^ = .09 | | | | *F*(8, 285) = 3.93, *p* < .001, R^2^ = .10 | | | | *F*(8, 285) = 3.86, *p* < .001, R^2^ = .10 | | | |
| *Note.* Gender 1 = Male | | | | | | | | | | | | | | | | | | | | |

| **Table C1.3**  *Multiple Linear Regression predicting Willingness to Hire Ex-Prisoner in Study 3a (US)* | | | | | | | | | | | | |
| --- | --- | --- | --- | --- | --- | --- | --- | --- | --- | --- | --- | --- |
| Variables | Model 1 | | | | Model 2 | | | | Model 3 | | | |
|  | B | SE | *β* | *p* | B | SE | *β* | *p* | B | SE | *β* | *p* |
| Dummy I: Dysphoric experience | -0.10 | 0.14 | -.01 | .940 | -0.03 | 0.13 | -.01 | .830 | 0.02 | 0.13 | .01 | .886 |
| Dummy II: Non-dysphoric experience | 0.02 | 0.14 | .01 | .890 | 0.01 | 0.14 | .00 | .946 | -0.01 | 0.13 | -.01 | .924 |
| Identity fusion |  |  |  |  | 0.38 | 0.06 | .34 | .000 | 0.35 | 0.06 | .31 | .000 |
| Experience transformativeness |  |  |  |  |  |  |  |  | -0.06 | 0.07 | -.06 | .328 |
| Sport fandom |  |  |  |  |  |  |  |  | 0.05 | 0.06 | .05 | .410 |
| Open-mindedness |  |  |  |  |  |  |  |  | 0.18 | 0.06 | .16 | .003 |
| Political ideology |  |  |  |  |  |  |  |  | -0.05 | 0.02 | -.13 | .018 |
| Contact ex-prisoners |  |  |  |  |  |  |  |  | 0.17 | 0.09 | .11 | .054 |
| Victim status |  |  |  |  |  |  |  |  | 0.27 | 0.12 | .13 | .020 |
| Age |  |  |  |  |  |  |  |  | -0.01 | 0.00 | -.18 | .001 |
| Model Summary | *F*(2,291) = 0.02, *p* = .978, R^2^ = .00 | | | | *F*(3, 290) = 12.57, *p* < .001, R^2^ = .12 | | | | *F*(10, 283) = 9.17, *p* < .001, R^2^ = .25 | | | |
| *Note.* Victim status 1 = Yes | | | | | | | | | | | | |

| **Table C1.4**  *Multiple Linear Regression predicting Perceived Future Chances of the Ex-Prisoner in Study 3a (US)* | | | | | | | | | | | | |
| --- | --- | --- | --- | --- | --- | --- | --- | --- | --- | --- | --- | --- |
| Variables | Model 1 | | | | Model 2 | | | | Model 3 | | | |
|  | B | SE | *β* | *p* | B | SE | *β* | *p* | B | SE | *β* | *p* |
| Dummy I: Dysphoric experience | -0.00 | 0.12 | -.00 | .985 | -0.02 | 0.11 | -.01 | .867 | 0.03 | 0.11 | .02 | .801 |
| Dummy II: Non-dysphoric experience | -0.06 | 0.12 | -.03 | .653 | -0.07 | 0.11 | -.04 | .569 | -0.05 | 0.11 | -.03 | .651 |
| Identity fusion |  |  |  |  | 0.35 | 0.05 | .37 | .000 | 0.33 | 0.05 | .35 | .000 |
| Experience transformativeness |  |  |  |  |  |  |  |  | -0.08 | 0.06 | -.10 | .144 |
| Sport fandom |  |  |  |  |  |  |  |  | -0.02 | 0.05 | -.02 | .742 |
| Open-mindedness |  |  |  |  |  |  |  |  | 0.18 | 0.05 | .19 | .001 |
| Contact ex-prisoner |  |  |  |  |  |  |  |  | 0.09 | 0.07 | .07 | .222 |
| SES |  |  |  |  |  |  |  |  | 0.01 | 0.03 | .02 | .669 |
| Model Summary | *F*(2, 291) = 1.24, *p* = .884, R^2^ = .00 | | | | *F*(3, 290) = 15.17, *p* < .001, R^2^ = .14 | | | | *F*(8, 285) = 7.74, *p* < .001, R^2^ = .18 | | | |

| **Table C2.1**  *Cronbach Alphas, Descriptive Statistics and Correlations of Variables in Study 3b (UK)* | | | | | | | | | | | | | | | | | | | | |  |
| --- | --- | --- | --- | --- | --- | --- | --- | --- | --- | --- | --- | --- | --- | --- | --- | --- | --- | --- | --- | --- | --- |
| Variables | α | M | (SD) | 2. | 3. | 4. | 5. | 6. | 7. | 8. | 9. | 10. | 11. | 12. | 13. | 14. | 15. | 16. | 17. | 18. | |
| 1. Willingness to hire |  | 4.60 | 0.98 | .49*** | .35*** | -.03 | .07 | .04 | .06 | -.10 | .01 | .04 | -.07 | -.07 | -.06 | -.19*** | .26*** | .13* | -.07 | .11* | |
| 1. Perceived chances to desist |  | 3.85 | 0.69 |  | .30*** | .10 | -.02 | .03 | .07 | .00 | .05 | .12* | .03 | -.01 | .04 | -.09 | .19*** | .09 | .03 | .04 | |
| 1. Identity fusion | .84 | 2.43 | 0.84 |  |  | -.07 | .11 | .19** | .09 | .03 | .09 | -.04 | -.08 | -.11 | .01 | .06 | .17** | .01 | -.12* | .14* | |
| 1. Dysphoric experience |  | 0.35 | 0.48 |  |  |  | -.47*** | -.07 | -.06 | -.10 | -.10 | -.01 | .03 | .04 | -.04 | .02 | -.02 | -.06 | .02 | .01 | |
| 1. Non-dysphoric experience |  | 0.30 | 0.46 |  |  |  |  | .09 | -.04 | .10 | .01 | -.02 | -.05 | -.05 | -.04 | -.09 | .00 | .09 | .10 | -.07 | |
| 1. Experience transformativeness | .80 | 3.26 | 0.85 |  |  |  |  |  | .49*** | -.01 | .15* | -.11* | -.05 | -.04 | .06 | -.06 | .07 | .09 | .00 | .05 | |
| 1. Sport fandom |  | 3.63 | 1.11 |  |  |  |  |  |  | -.05 | .23*** | .03 | -.02 | -.15 | .03 | -.01 | .08 | -.01 | -.13* | .02 | |
| 1. Age |  | 41.12 | 14.25 |  |  |  |  |  |  |  | -.03 | .29*** | .17** | -.09 | .45*** | .19*** | -.13* | .14* | .21*** | .13* | |
| 1. Gender |  | 0.81 | 0.40 |  |  |  |  |  |  |  |  | .06 | .01 | -.07 | .06 | .11 | -.05 | -.00 | -.08 | .06 | |
| 1. Ethnicity |  | 0.83 | 0.38 |  |  |  |  |  |  |  |  |  | .01 | -.13* | .18 | .07 | -.05 | .02 | .06 | -.01 | |
| 1. SES |  | 5.82 | 1.40 |  |  |  |  |  |  |  |  |  |  | .23*** | .24*** | .09 | -.06 | .01 | -.00 | .19** | |
| 1. Education |  | 3.68 | 0.84 |  |  |  |  |  |  |  |  |  |  |  | .12* | -.16** | .03 | -.11 | .05 | -.01 | |
| 1. Hiring experience |  | 1.52 | 1.61 |  |  |  |  |  |  |  |  |  |  |  |  | .07 | -.09 | .08 | .11 | .06 | |
| 1. Political Ideology | .95 | 4.50 | 2.07 |  |  |  |  |  |  |  |  |  |  |  |  |  | -.26*** | -.04 | -.03 | .00 | |
| 1. Open-mindedness | .92 | 3.44 | 0.82 |  |  |  |  |  |  |  |  |  |  |  |  |  |  | -.02 | -.07 | .08 | |
| 1. Contact ex-prisoner |  | 1.57 | 0.62 |  |  |  |  |  |  |  |  |  |  |  |  |  |  |  | .15** | -.09 | |
| 1. Victim status |  | 0.59 | 0.49 |  |  |  |  |  |  |  |  |  |  |  |  |  |  |  |  | -.12* | |
| 1. Social desirability | .73 | 10.18 | 3.17 |  |  |  |  |  |  |  |  |  |  |  |  |  |  |  |  |  | |
| *Note.* Gender 1 = Male, Ethnicity 1 = White, Victim status 1 = Yes  **p <* .05, ***p* < .01, ****p* <.001 | | | | | | | | | | | | | | | | | | | | |  |

| **Table C2.2**  *Multiple Regression Analyses Predicting Identity Fusion to the Ex-Prisoner(UK) in Study 3b* | | | | | | | | | | | | | | | | | | | | |
| --- | --- | --- | --- | --- | --- | --- | --- | --- | --- | --- | --- | --- | --- | --- | --- | --- | --- | --- | --- | --- |
| Variables | Model 1 | | | | Model 2 | | | | Model 3 | | | | Model 4 | | | | Model 5 | | | |
|  | B | SE | *β* | *p* | B | SE | *β* | *p* | B | SE | *β* | *p* | B | SE | *β* | *p* | B | SE | *β* | *p* |
| Dummy I: dysphoric experience | -0.05 | 0.12 | -.03 | .661 | -0.04 | 0.11 | -.02 | .742 | -0.01 | 0.11 | -.01 | .902 | -1.01 | 0.42 | -.57 | .017 | -1.01 | 0.40 | -.57 | .012 |
| Dummy II: non-dysphoric experience | 0.17 | 0.12 | .10 | .147 | 0.15 | 0.12 | .08 | .205 | 0.20 | 0.12 | .11 | .097 | -0.79 | 0.48 | -.43 | .105 | -0.45 | 0.41 | -.24 | .279 |
| Experience transformativeness | - | - | - | - | 0.17 | 0.07 | .17 | .009 | 0.16 | 0.06 | .17 | .010 | -0.04 | 0.10 | -.04 | .714 | 0.19 | 0.06 | .19 | .004 |
| Football fandom | - | - | - | - | 0.01 | 0.05 | .01 | .921 | -0.01 | 0.05 | -.02 | .817 | -0.00 | 0.05 | -.00 | .987 | -0.17 | 0.08 | -.23 | .038 |
| Open-mindedness | - | - | - | - | - | - | - | - | 0.14 | 0.06 | .14 | .014 | 0.15 | 0.06 | .14 | .010 | 0.15 | 0.06 | .15 | .008 |
| Victim status | - | - | - | - | - | - | - | - | -0.28 | 0.10 | -.10 | .069 | -0.19 | 0.10 | -.11 | .051 | -0.17 | 0.10 | -.10 | .080 |
| Social desirability | - | - | - | - | - | - | - | - | 0.03 | 0.10 | .11 | .043 | 0.03 | 0.02 | .12 | .027 | 0.03 | 0.02 | .11 | .045 |
| Dummy I X Exp transformativeness |  |  |  |  |  |  |  |  |  |  |  |  | 0.31 | 0.13 | .59 | .014 | - | - | - | - |
| Dummy II X Exp transformativeness |  |  |  |  |  |  |  |  |  |  |  |  | 0.30 | 0.14 | .57 | .035 | - | - | - | - |
| Dummy I X Football fandom |  |  |  |  |  |  |  |  |  |  |  |  | - | - | - | - | 0.27 | 0.10 | .58 | .010 |
| Dummy II X Football fandom |  |  |  |  |  |  |  |  |  |  |  |  | - | - | - | - | 0.17 | 0.11 | .35 | .115 |
| Model Summary | *F*(2,300) = 1.88, *p* = .155, R^2^ = .01 | | | | *F*(4, 298) = 3.36, *p* = .010, R^2^ = .04 | | | | *F*(7, 295) = 4.27, *p* < .001, R^2^ = .09 | | | | F(9, 293) = 4.20, *p* < .001, R^2^ = .11 | | | | *F*(9, 293) = 4.14, *p* < .001, R^2^ = .11 | | | |

**Figure C2**

*Conditional Effects of Experimental Conditions on Identity fusion at +/-1SD of Experience Transformativeness and at +/-1SD of Football Fandom in S3b*

| **Table C2.3**  *Multiple Linear Regression Predicting Willingness to Hire Ex-Prisoner in Study 3b (UK) Based on the Experimental conditions only (Model 1), and Based on the Experimental conditions via Identity Fusion as moderated by Experience transformativeness (Model 2.1) or Sport Fandom (Model 2.2)* | | | | | | | | | | | | |
| --- | --- | --- | --- | --- | --- | --- | --- | --- | --- | --- | --- | --- |
| Variables | Model 1 | | | | Model 2.1 | | | | Model 2.2 | | | |
|  | B | SE | *β* | *p* | B | SE | *β* | *p* | B | SE | *β* | *p* |
| Dummy I: Dysphoric experience | 0.01 | 0.14 | .00 | .963 | 0.04 | 0.12 | .02 | .752 | 0.03 | 0.12 | .01 | .805 |
| Dummy II: Non-dysphoric experience | 0.16 | 0.14 | .07 | .269 | 0.06 | 0.13 | .03 | .665 | 0.06 | 0.13 | .03 | .653 |
| Identity fusion | - | - | - | - | 0.37 | 0.06 | .32 | <.001 | 0.39 | 0.06 | .33 | <.001 |
| Experience transformativeness | - | - | - | - | - | - | - | - | -0.07 | 0.06 | -.06 | .254 |
| Football fandom | - | - | - | - | 0.02 | 0.05 | .02 | .673 | - | - | - | - |
| Open-mindedness | - | - | - | - | 0.19 | 0.07 | .16 | .005 | 0.19 | 0.07 | .16 | .004 |
| Political ideology | - | - | - | - | -0.08 | 0.03 | -.16 | .003 | -0.08 | 0.03 | -.17 | .002 |
| Contact ex-prisoners | - | - | - | - | 0.20 | 0.08 | .12 | .020 | 0.20 | 0.08 | .13 | .016 |
| Social desirability | - | - | - | - | 0.02 | 0.02 | .07 | .194 | 0.02 | 0.02 | .07 | .180 |
|  |  |  |  |  |  |  |  |  |  |  |  |  |
|  |  |  |  |  | Moderator = Experience transformativeness | | | | Moderator = Football fandom | | | |
| Dummy I: Con. indirect effect (-1SD of moderator) |  |  |  |  | B = -0.10, SE = 0.06, 95%CI [-.224, .015] | | | | B = -0.12, SE = 0.07, 95%CI [-.265, -.003] | | | |
| Dummy I: Con. indirect effect (+1SD of moderator) |  |  |  |  | B = 0.09, SE = 0.07, 95%CI [-.030, .227] | | | | B = 0.10, SE = 0.07, 95%CI [-.030, .236] | | | |
| Dummy I: Index of moderated mediation |  |  |  |  | Index = 0.11, SE = 0.06, 95%CI [.009, .225] | | | | Index = 0.10, SE = 0.05, 95%CI [.017, .199] | | | |
| Dummy II: Con. indirect effect (-1SD of moderator) |  |  |  |  | B = -0.02, SE = 0.07, 95%CI [-.149, .118] | | | | B = -0.01, SE = 0.07, 95%CI [-.135, .129] | | | |
| Dummy II: Con. indirect effect (+1SD of moderator) |  |  |  |  | B = 0.16, SE = 0.07, 95%CI [.043, .227] | | | | B = 0.14, SE = 0.07, 95%CI [.007, .288] | | | |
| Dummy II: Index of moderated mediation |  |  |  |  | Index = 0.10, SE = 0.06, 95%CI [.003, .217] | | | | Index = 0.07, SE = 0.05, 95%CI [-.021, .158] | | | |
| Model Summary | *F*(2,300) = 0.76, *p* = .469, R^2^ = .01 | | | | *F*(8, 293) = 9.67, *p* < .001, R^2^ = .21 | | | | *F*(9, 293) = 8.91, *p* < .001, R^2^=.22 | | | |

| **Table C2.4**  *Multiple Linear Regression Analyses Predicting Perceived Future Chances of the Ex-Prisoner in Study 3b (UK) Based on the Experimental conditions only (Model 1), and Based on the Experimental conditions via Identity Fusion on Experience transformativeness (Model 2.1) or Sport Fandom (Model 2.2)* | | | | | | | | | | | | |
| --- | --- | --- | --- | --- | --- | --- | --- | --- | --- | --- | --- | --- |
| Variables | Model 1 | | | | Model 2.1 | | | | Model 2.2 | | | |
|  | B | SE | *β* | *p* | B | SE | *β* | *p* | B | SE | *β* | *p* |
| Dummy I: Dysphoric experience | 0.18 | 0.10 | .12 | .067 | 0.20 | 0.09 | .14 | .024 | 0.20 | 0.09 | .13 | .028 |
| Dummy II: Non-dysphoric experience | 0.06 | 0.10 | .04 | .575 | 0.03 | 0.09 | .02 | .761 | 0.02 | 0.09 | .02 | .790 |
| Identity fusion | - | - | - | - | 0.24 | 0.05 | .29 | <.001 | 0.24 | 0.05 | .29 | <.001 |
| Experience transformativeness | - | - | - | - | - | - | - | - | -0.01 | 0.04 | -.02 | .780 |
| Football fandom | - | - | - | - | 0.02 | 0.03 | .03 | .523 | - | - | - | - |
| Open-mindedness | - | - | - | - | 0.13 | 0.05 | .15 | .006 | 0.13 | 0.05 | .15 | .005 |
| Ethnicity | - | - | - | - | 0.25 | 0.10 | .13 | .013 | 0.25 | 0.10 | .13 | .013 |
|  |  |  |  |  |  |  |  |  |  |  |  |  |
|  |  |  |  |  | Moderator = Experience transformativeness | | | | Moderator = Football fandom | | | |
| Dummy I: Con. indirect effect (-1SD of moderator) |  |  |  |  | B = -0.06, SE = 0.04, 95%CI [-.146, .004] | | | | B = -0.09, SE = 0.04, 95%CI [-.176, -.012] | | | |
| Dummy I: Con. indirect effect (+1SD of moderator) |  |  |  |  | B = 0.05, SE = 0.05, 95%CI [-.028, .148] | | | | B = 0.06, SE = 0.04, 95%CI [-.014, .159] | | | |
| Dummy I: Index of moderated mediation |  |  |  |  | Index = 0.07, SE = 0.04, 95%CI [.004, 140] | | | | Index = 0.07, SE = 0.03, 95%CI [.016, .133] | | | |
| Dummy II: Con. indirect effect (-1SD of moderator) |  |  |  |  | B = -0.02, SE = 0.04, 95%CI [-.108, .059] | | | | B = -0.01, SE = 0.04, 95%CI [-.010, .160] | | | |
| Dummy II: Con. indirect effect (+1SD of moderator) |  |  |  |  | B = 0.09, SE = 0.04, 95%CI [.014, .180] | | | | B = 0.07, SE = 0.04, 95%CI [.007, .288] | | | |
| Dummy II: Index of moderated mediation |  |  |  |  | Index = 0.06, SE = 0.04, 95%CI [.004, .140] | | | | Index = 0.04, SE = 0.03  , 95%CI [-.015, .097] | | | |
| Model Summary | *F*(2,300) = 1.75, *p* = .175, R^2^ = .01 | | | | *F*(6, 296) = 8.51, *p* < .001, R^2^ = .15 | | | | *F*(F(6, 296) = 8.44, *p* < .001, R^2^ = .15 | | | |
| *Note.* Ethnicity 1 = White | | | | | | | | | | | | |

# Supplementary Information D: Studies 4a (US) & 4b (UK)

**Study details**

For S4a, 272 American citizens was recruited online. Ten participants were excluded because they did not pass an attention check. The final sample consisted of 262 participants (M_age_ = 46.55), the majority of whom were female (50.4%, 47.7% male, 1.9% non-binary/third gender), white (78.2%; 11.1% Black, 5% Hispanic, 2.7% Asian, 2.3% mixed, 0.4% other, 0.4% prefer not to say) and had experience making hiring decisions (98.1%). Ethical approval for this study was granted by the ethics board of the School of Anthropology and Conservation at the University of Kent: Ethics ID: 20231684156406455.

For S4b, 270 UK nationals was recruited. 4 participants were excluded because they failed an attention check. The final sample consisted of N = 266 participants, (M_age_ = 45.84), the majority of whom were male (50.4%, 49.2% female, 0.4% non-binary/third gender), white (92.5%; 2.6% Black, 1.5% Asian, 3% mixed, 0.4% prefer not to say) and had experience making hiring decisions (99.2%). Ethical approval for this study was granted by the ethics board of the School of Anthropology and Conservation at the University of Kent: Ethics ID: 20231702891636577.

| **Table D1.1**  *Cronbach Alphas, Descriptive Statistics and Correlations of Variables in Study 4a (US)* | | | | | | | | | | | | | | | | | | | |
| --- | --- | --- | --- | --- | --- | --- | --- | --- | --- | --- | --- | --- | --- | --- | --- | --- | --- | --- | --- |
| Variables | α | M | (SD) | 2. | 3. | 4. | 5. | 6. | 7. | 8. | 9. | 10. | 11. | 12. | 13. | 14. | 15. | 16. | 17. |
| 1. Willingness to hire |  | 4.58 | 0.92 | .64*** | .27*** | .00 | .08 | .28*** | -.08 | -.07 | -.02 | -.10 | -.06 | -.00 | -.25*** | .35*** | .12 | -.04 | -.02 |
| 1. Perceived chances to desist |  | 3.81 | 0.69 |  | .30*** | -.02 | .17** | .33*** | .06 | -.06 | .03 | -.04 | -.02 | .03 | -.21** | .30*** | .02 | -.08 | .04 |
| 1. Identity fusion | .91 | 3.19 | 1.41 |  |  | .00 | .13* | .19** | -.03 | .12 | -.17** | .03 | .03 | .09 | .04 | .15* | .07 | -.28*** | .20** |
| 1. Prison condition ^a^ |  | 0.50 | 0.50 |  |  |  | .01 | .18** | .09 | -.02 | .07 | .03 | -.05 | -.03 | .03 | -.01 | .00 | .05 | .03 |
| 1. Personal Experience transformativeness | .72 | 6.06 | 0.82 |  |  |  |  | .33*** | .17** | -.10 | -.01 | .07 | -.05 | -.05 | -.02 | .31*** | -.02 | .04 | .08 |
| 1. Perceived Experience transformativeness | .80 | 5.84 | 0.90 |  |  |  |  |  | .15* | -.01 | .02 | -.04 | -.06 | -.01 | -.18** | .30*** | -.02 | .17** | .05 |
| 1. Age |  | 46.55 | 13.89 |  |  |  |  |  |  | -.08 | .13* | .13* | -.03 | .12 | -.04 | -.01 | -.09 | .09 | .15* |
| 1. Gender |  | 0.48 | .50 |  |  |  |  |  |  |  | -.09 | .06 | .15* | .15* | .17** | -.08 | -.04 | -.05 | .07 |
| 1. Ethnicity |  | 0.78 | 0.41 |  |  |  |  |  |  |  |  | -.00 | .06 | -.00 | -.11 | -.05 | -.18** | -.03 | -.14* |
| 1. SES |  | 5.54 | 1.75 |  |  |  |  |  |  |  |  |  | .45*** | .20** | .02 | .03 | -.18** | -.15* | .05 |
| 1. Education |  | 3.81 | 1.03 |  |  |  |  |  |  |  |  |  |  | .08 | .01 | -.01 | -.09 | -.16** | -.12* |
| 1. Hiring experience |  | 2.31 | 1.72 |  |  |  |  |  |  |  |  |  |  |  | .04 | .11 | .10 | -.08 | .12 |
| 1. Political ideology | .96 | 4.07 | 3.00 |  |  |  |  |  |  |  |  |  |  |  |  | -.23*** | .04 | -.01 | .09 |
| 1. Open-mindedness | .94 | 4.29 | 1.07 |  |  |  |  |  |  |  |  |  |  |  |  |  | .16** | .08 | .11 |
| 1. Contact ex-prisoner |  | 0.84 | 0.73 |  |  |  |  |  |  |  |  |  |  |  |  |  |  | .24*** | .09 |
| 1. Victim status |  | 0.53 | 0.50 |  |  |  |  |  |  |  |  |  |  |  |  |  |  |  | -.05 |
| 1. Social desirability | .82 | 9.06 | 3.99 |  |  |  |  |  |  |  |  |  |  |  |  |  |  |  |  |
| *Note.* ^a^ 1 = good , Gender 1 = male, Ethnicity 1 = white  * *p* < .05, ** *p* < .01, *** *p* < .001 | | | | | | | | | | | | | | | | | | | |

| **Table D1.2**  *Multiple Linear Regression Models Predicting Fusion to the Ex-Prisoner based on Personal- and Perceived Experience Transformativeness, Their Interaction Term and Control Variables in Study 4a (US)* | | | | | | | | | | | | |
| --- | --- | --- | --- | --- | --- | --- | --- | --- | --- | --- | --- | --- |
| Variables | Model 1 | | | | Model 2 | | | | Model 3 | | | |
|  | B | SE | *β* | *p* | B | SE | *β* | *p* | B | SE | *β* | *p* |
| Participant experience transformativeness | 0.22 | 0.11 | .13 | .037 | -1.12 | 0.62 | -.65 | .072 | -1.11 | 0.58 | -.64 | .057 |
| Applicant experience transformativeness | - | - | - | - | -1.05 | 0.64 | -.67 | .104 | -0.91 | 0.60 | -.58 | .130 |
| Participant X Applicant experience transformativeness | - | - | - | - | 0.22 | 0.11 | 1.27 | .041 | 0.20 | 0.10 | 1.20 | .040 |
| Open-mindedness | - | - | - | - | - | - | - | - | 0.09 | 0.08 | .07 | .248 |
| Victim status | - | - | - | - | - | - | - | - | -0.91 | 0.16 | -.32 | .000 |
| Social desirability | - | - | - | - | - | - | - | - | 0.05 | 0.02 | .14 | .018 |
| Ethnicity | - | - | - | - | - | - | - | - | -0.51 | 0.19 | -.15 | .010 |
| Model Summary | *F*(1,260) = 4.38, *p* = .037, R^2^ = .02 | | | | *F*(3,258) = 4.96, *p* = .002, R^2^ = .06 | | | | *F*(7,254) = 9.50, *p* <.001, R^2^ = .21 | | | |
| *Note.* Victim status 1 = Yes, Ethnicity 1 = White | | | | | | | | | | | | |

| **Table D1.3**  *Multiple Linear Regression Analyses Predicting Willingness to Hire Ex-Prisoner (Model 2) and Perceived Chances of the Ex-Prisoner(Model 3) Based on the al Moderated Mediation Model (Model 11) in Hayes’ (2022) Process Macro in Study 4a (US)* | | | | | | | | | | | | |
| --- | --- | --- | --- | --- | --- | --- | --- | --- | --- | --- | --- | --- |
| Variables | Model 1 (Identity Fusion) | | | | Model 2 (Willingness to hire) | | | | Model 3 (Perceived chances to desist) | | | |
|  | B | SE | *t* | *p* | B | SE | *t* | *p* | B | SE | *t* | *p* |
| Identity Fusion | - | - | - | - | 0.16 | 0.04 | 3.99 | <.001 | 0.13 | 0.03 | 4.20 | <.001 |
| Personal Experience transformativeness (X) | -5.76 | 2.10 | -2.75 | .006 | -0.05 | 0.07 | -0.75 | .457 | 0.05 | 0.05 | 1.07 | .284 |
| Perceived Experience transformativeness (W) | -5.61 | 2.20 | -2.55 | .011 | - | - | - | - | - | - | - | - |
| X*W | 1.03 | 0.36 | 2.88 | .004 | - | - | - | - | - | - | - | - |
| Prison (Z) | -16.16 | 7.35 | -2.20 | .029 | - | - | - | - | - | - | - | - |
| X*Z | 2.89 | 1.23 | 2.35 | .020 | - | - | - | - | - | - | - | - |
| W*Z | 2.89 | 1.27 | 2.27 | .024 | - | - | - | - | - | - | - | - |
| X*W*Z | -0.51 | 0.21 | -2.45 | .015 |  |  |  |  |  |  |  |  |
| Victim status | -0.94 | 0.16 | -5.86 | <.001 | -0.01 | 0.11 | -0.09 | .930 | -0.04 | 0.08 | -0.46 | .648 |
| Open-mindedness | 0.11 | 0.08 | 1.40 | .162 | 0.29 | 0.05 | 5.65 | <.001 | 0.16 | 0.04 | 4.07 | <.001 |
| Social desirability | 0.05 | 0.02 | 2.31 | .022 | -0.02 | 0.01 | -1.65 | .100 | -0.01 | 0.01 | -0.55 | .580 |
| Ethnicity | -0.45 | 0.19 | -2.33 | .021 | 0.06 | 0.13 | 0.45 | .656 | 0.14 | 0.10 | 1.41 | .161 |
|  |  | | | |  | | | |  | | | |
| Model Summary | *F*(11, 250) = 6.82, *p* < .001, R^2^ = .23 | | | | *F*(6, 255) = 9.58, *p* < .001, R^2^ = .18 | | | | *F*(6, 255) = 8.44, *p* < .001, R^2^ = .17 | | | |
|  |  | | | |  | | | |  | | | |
|  | al X*W interaction at value of Z = 1 (good prison ); *F*(1,250) = 9.51, *p* = .002 | | | | Index of moderated moderated mediation;  Index = -0.08, SE = 0.04, 95% CI [-.168, -.016] | | | | Index of moderated moderated mediation;  Index = -0.07, SE = 0.03, 95% CI [-.132, -.014] | | | |
|  | al X*W interaction at value of Z = 2 (bad prison ); *F*(1,250) = 0.00, *p* = .976 | | | | Index of al moderated mediation by W at value of Z = 1 (good prison );  Index = 0.08, SE = 0.03, 95% CI [.026, .151] | | | | Index of al moderated mediation by W at value of Z = 1 (good prison );  Index = 0.07, SE = 0.03, 95% CI [.022, .119] | | | |
|  |  | | | | Index of al moderated mediation by W at value of Z = 2 (bad prison );  Index = 0.00, SE = 0.02, 95% CI [-.047, .037] | | | | Index of al moderated mediation by W at value of Z = 2 (bad prison );  Index = 0.00, SE = 0.02, 95% CI [-.040, .031] | | | |
| *Note.* Prison- (Z) 1 = good prison, 2 = bad prison, Victim status 1 = Yes, Ethnicity 1 = White | | | | | | | | | | | | |

| **Table D2.1**  *Cronbach Alphas, Descriptive Statistics and Correlations of Variables in Study 4b (UK)* | | | | | | | | | | | | | | | | | | | |
| --- | --- | --- | --- | --- | --- | --- | --- | --- | --- | --- | --- | --- | --- | --- | --- | --- | --- | --- | --- |
| Variables | α | M | (SD) | 2. | 3. | 4. | 5. | 6. | 7. | 8. | 9. | 10. | 11. | 12. | 13. | 14. | 15. | 16. | 17. |
| 1. Willingness to hire |  | 4.20 | .97 | .52*** | .42*** | .03 | .02 | .52*** | .00 | .06 | .01 | -.01 | -.09 | .38*** | -.20*** | .01 | .09 | .00 | -.05 |
| 1. Perceived chances to desist |  | 3.62 | .67 |  | .27*** | -.08 | .04 | .34*** | .00 | -.01 | .09 | -.04 | -.01 | .26*** | -.08 | .04 | .01 | .02 | -.06 |
| 1. Identity fusion | .88 | 2.90 | 1.20 |  |  | -.03 | .18** | .24*** | .11 | .16** | -.07 | -.05 | -.05 | .26*** | -.11 | -.03 | .10 | -.06 | .05 |
| 1. Prison ^a^ |  | 1.51 | .50 |  |  |  | -.02 | .10 | -.06 | -.05 | -.02 | -.01 | .03 | .00 | .01 | .04 | -.07 | .07 | .00 |
| 1. Participant experience transformativeness |  | 5.99 | 1.13 |  |  |  |  | .12 | .04 | -.08 | -.03 | -.01 | .09 | .10 | -.06 | -.01 | .07 | .01 | .05 |
| 1. Applicant experience transformativeness |  | 5.85 | .97 |  |  |  |  |  | -.11 | -.03 | -.03 | .06 | .01 | .32*** | -.22*** | -.02 | .02 | .04 | -.05 |
| 1. Age |  | 45.84 | 12.75 |  |  |  |  |  |  | .08 | .15* | -.07 | .13* | .03 | .09 | .20** | .10 | .05 | .10 |
| 1. Gender |  | .50 | .50 |  |  |  |  |  |  |  | .06 | .03 | -.01 | .11 | .08 | .09 | .07 | -.03 | .09 |
| 1. Ethnicity |  | .92 | .26 |  |  |  |  |  |  |  |  | -.24*** | -.12* | -.03 | .11 | .07 | .06 | .08 | -.02 |
| 1. Education |  | 3.77 | .97 |  |  |  |  |  |  |  |  |  | .30*** | -.02 | -.14* | .03 | -.10 | .05 | .01 |
| 1. SES |  | 5.77 | 1.59 |  |  |  |  |  |  |  |  |  |  | .06 | .03 | .06 | -.13* | .00 | .09 |
| 1. Open-mindedness | .95 | 4.07 | .96 |  |  |  |  |  |  |  |  |  |  |  | -.27*** | -.02 | -.01 | .00 | .08 |
| 1. Political ideology | .95 | 3.97 | 2.14 |  |  |  |  |  |  |  |  |  |  |  |  | .04 | -.07 | -.10 | .00 |
| 1. Hiring experience |  | 2.30 | 1.68 |  |  |  |  |  |  |  |  |  |  |  |  |  | .15* | -.01 | .11 |
| 1. Contact ex-prisoner |  | .60 | .73 |  |  |  |  |  |  |  |  |  |  |  |  |  |  | .13* | -.10 |
| 1. Victim status |  | .68 | .47 |  |  |  |  |  |  |  |  |  |  |  |  |  |  |  | -.15* |
| 1. Social desirability | .74 | 9.59 | 3.25 |  |  |  |  |  |  |  |  |  |  |  |  |  |  |  |  |
| *Note. N = 266,* ^a^ 1 = good , Gender 1 = male, Ethnicity 1 = white  * *p* < .05, ** *p* < .01, *** *p* < .001 | | | | | | | | | | | | | | | | | | | |

| **Table D2.2**  *Multiple Linear Regression Models Predicting Fusion to the Ex-Prisoner based on Personal- and Perceived Experience Transformativeness, Their Interaction Term and Control Variables in Study 4b (UK)* | | | | | | | | | | | | | | | | |
| --- | --- | --- | --- | --- | --- | --- | --- | --- | --- | --- | --- | --- | --- | --- | --- | --- |
| Variables | Model 1 | | | | Model 2 | | | | Model 3 | | | | Model 4 | | | |
|  | B | SE | *β* | *p* | B | SE | *β* | *p* | B | SE | *β* | *p* | B | SE | *β* | *p* |
| Participant experience transformativeness | 0.19 | 0.06 | .18 | .003 | 0.16 | 0.06 | .15 | .011 | 0.05 | 0.35 | .04 | .894 | .164 | .062 | .155 | .008 |
| Applicant experience transformativeness | - | - | - | - | 0.27 | 0.07 | .22 | <.001 | 0.15 | 0.37 | .12 | .680 | .210 | .075 | .170 | .006 |
| Participant X Applicant experience transformativeness | - | - | - | - | - | - | - | - | 0.02 | 0.06 | .16 | .738 | - | - | - | - |
| Open-mindedness | - | - | - | - | - | - | - | - | - | - | - | - | .214 | .076 | .172 | .005 |
| Gender | - | - | - | - | - | - | - | - | - | - | - | - | .384 | .139 | .161 | .006 |
| Model Summary | *F*(1,264) = 8.81, *p* = .003, R^2^ = .03 | | | | *F*(2,263) = 11.47, *p* < .001, R^2^ = .08 | | | | *F*(3,262) = 7.67, *p* <.001, R^2^ = .08 | | | | *F*(4,261) = 10.56, *p* <.001, R^2^ = .14 | | | |
| *Note.* Gender 1 = Male | | | | | | | | | | | | | | | | |

| **Table D2.3**  *Multiple Linear Regression Analyses Predicting Willingness to Hire Ex-Prisoner (Model 2) and Perceived Future Chances of the Ex-Prisoner(Model 3) via Identity Fusion, Based on Model 4 of Hayes’ (2022) Process Macro in Study 4b* | | | | | | | | | | | | | | | |
| --- | --- | --- | --- | --- | --- | --- | --- | --- | --- | --- | --- | --- | --- | --- | --- |
| Variables | Model 1 (Identity fusion) | | | | | Model 2 (Willingness to hire) | | | | | Model 3 (Perceived chances to desist) | | | | |
|  | B | SE | *t* | *β* | *p* | B | SE | *t* | *β* | *p* | B | SE | *t* | *β* | *p* |
| Identity fusion | - | - | - | - | - | 0.23 | 0.04 | 5.64 | .29 | <.001 | 0.11 | 0.03 | 3.17 | .19 | .002 |
| Participant experience transformativeness | 0.16 | 0.06 | 2.65 | .15 | .009 | -0.08 | 0.04 | -1.98 | -.10 | .049 | -0.03 | 0.03 | -0.76 | -.04 | .446 |
| Applicant experience transformativeness | 0.20 | 0.08 | 2.69 | .17 | .008 | 0.39 | 0.05 | 7.43 | .39 | <.001 | 0.18 | 0.04 | 4.20 | .26 | <.001 |
| Gender | 0.39 | 0.14 | 2.79 | .17 | .006 | 0.00 | 0.10 | 0.01 | .00 | .994 | -0.07 | 0.08 | -0.85 | -.05 | .395 |
| Open-mindedness | 0.21 | 0.08 | 2.61 | .16 | .009 | 0.18 | 0.05 | 3.36 | .18 | <.001 | 0.09 | 0.04 | 2.20 | .13 | .028 |
| Political ideology | -0.02 | 0.03 | -0.47 | -.03 | .639 | -0.02 | 0.02 | -0.92 | -.05 | .360 | - | - | - | - | - |
|  |  | | | | |  | | | | |  | | | | |
| Model Summary | *F*(5, 260) = 8.47, *p* <.001, R^2^ = .14 | | | | | *F*(6, 259) = 28.77, *p* <.001, R^2^ = .40 | | | | | *F*(5, 260) = 10.91, *p* <.001, R^2^ = .17 | | | | |
|  |  | | | | |  | | | | |  | | | | |
| Effects of participant experience transformativeness |  | | | | |  | | | | |  | | | | |
| Total effect |  | | | | | B = -0.05, SE = 0.04, 95% CI [-.132, .042] | | | | | B = -0.01, SE = 0.03, 95% CI [-.077, .060] | | | | |
| Indirect effect |  | | | | | B = 0.04, SE = 0.02, 95% CI [.011, .071] | | | | | B = 0.02, SE = 0.01, 95% CI [.004, .038] | | | | |
| Effects of applicant experience transformativeness |  | | | | |  | | | | |  | | | | |
| Total effect |  | | | | | B = 0.44, SE = 0.05, 95% CI [.330, .545] | | | | | B = 0.19, SE = 0.04, 95% CI [.117, .283] | | | | |
| Indirect effect |  | | | | | B = 0.05, SE = 0.02, 95% CI [.013, .092] | | | | | B = 0.02, SE = 0.01, 95% CI [.005, .046] | | | | |
| *Note.* Gender 1 = Male. Total/indirect effects of participant and applicant experience transformativeness were estimated separately | | | | | | | | | | | | | | | |
