# Supplementary material for: Can transformative experiences bridge the gap between receiving communities and formerly incarcerated persons?
Source: Br J Soc Psychol. 2025 May 6;64(3):e12886. doi: 10.1111/bjso.12886 (PMC12053958; doi:10.1111/bjso.12886)
Supplement: Supplementary file 2 — Appendix S2. [file BJSO-64-0-s001.docx]

Supplementary Information 2

Contents

[**Supplementary Information A: Stimuli for Studies 1a-b** 2](#_Toc157162530)

[**Study 1a** 2](#_Toc157162531)

[**Study 1b** 2](#_Toc157162532)

[**Supplementary Information B: Stimuli for Study 2** 4](#_Toc157162533)

[**Supplementary Information C: Stimuli for Studies 3a-b** 8](#_Toc157162534)

[**Study 3a (US)** 9](#_Toc157162535)

[**Study 3b (UK)** 13](#_Toc157162536)

[**Supplementary Information D: Stimuli for Studies 4a-b** 16](#_Toc157162537)

[**Study 4a (US)** 16](#_Toc157162538)

[**Study 4b (UK)** 16](#_Toc157162539)

[**Supplementary Information E: List of Scales for all Studies** 18](#_Toc157162540)

# **Supplementary Information A: Stimuli for Studies 1a-b**

Introduction text:

“Imagine you are a hiring manager at a building supply store. After reviewing a list of job applicants for an open cashier position, you determine that one candidate is the best qualified. However, the candidate has a special circumstance – he has a criminal record. A background check confirmed that the candidate has had two non-felony convictions in the past five years. He served six months in 2018 for one offense. He served 8 months in 2022 for the second offense.”

## **Study 1a**

Direct experience condition:

“During his interview, the applicant was asked about other important things to know about him. He said he felt his life was transformed by the loss of his mother due to cancer last year while he was in prison. He said it was really hard on him to not be able to be there for her. At first he was angry with himself for not being able to be there for his mother in her last days. Over time, he said he was able to fully grieve her loss and accept it by staying connected with his tight-knit family. He said he's especially motivated to stay on the straight and narrow because of his difficult experience.”

Indirect experience condition:

“During his interview, the applicant was asked about other important things to know about him. He described that while in prison, he felt his life was transformed after he witnessed his cellmate lose his mother due to cancer last year. He said that it was really hard on his cellmate to not be able to be there for her. At first his cellmate was angry with himself for not being able to be there for his mother in her last days. Over time, his cellmate said he was able to fully grieve her loss and accept it by staying connected with his tight-knit family. The applicant said he's especially motivated to stay on the straight and narrow because of his cellmate's difficult experience”

Control condition:

He described that after his last experience in prison, he is especially motivated to stay on the straight and narrow.

## **Study 1b**

Direct experience condition

“During his interview, the applicant was asked about other important things to know about him. He said he felt his life was transformed by the loss of his mother due to cancer last year while he was in prison. He said it was really hard on him to not be able to be there for her. At first he was angry with himself for not being able to be there for his mother in her last days. Over time, he said he was able to fully grieve her loss and accept it by staying connected with his tight-knit family. He said he's especially motivated to stay on the straight and narrow because of his difficult experience.”

Indirect experience condition:

“During his interview, the applicant was asked about other important things to know about him. He described that while in prison, he felt his life was transformed after he witnessed his cellmate lose his mother due to cancer last year. He said that it was really hard on his cellmate to not be able to be there for her. At first his cellmate was angry with himself for not being able to be there for his mother in her last days. Over time, his cellmate said he was able to fully grieve her loss and accept it by staying connected with his tight-knit family. The applicant said he's especially motivated to stay on the straight and narrow because of his cellmate's difficult experience”

Control condition:

He described that while in prison, he felt his life was transformed and that he's especially motivated to stay on the straight and narrow after his experience.

# **Supplementary Information B: Stimuli for Study 2**

Introduction text

Imagine you are a hiring manager at a building supply store. After reviewing a list of job applicants for an open cashier position, you determine that one candidate is the best qualified. However, the candidate has a special circumstance – he has a criminal record. A background check confirmed that the candidate has a conviction for which he served 22 months in prison.

You have a look at his social media presence and find a Facebook page. Please examine all information on the page to form a more accurate impression of the applicant.”

Dysphoric experience condition:


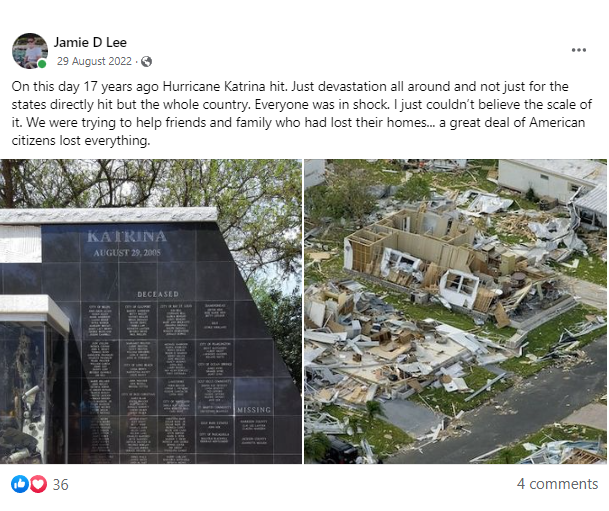


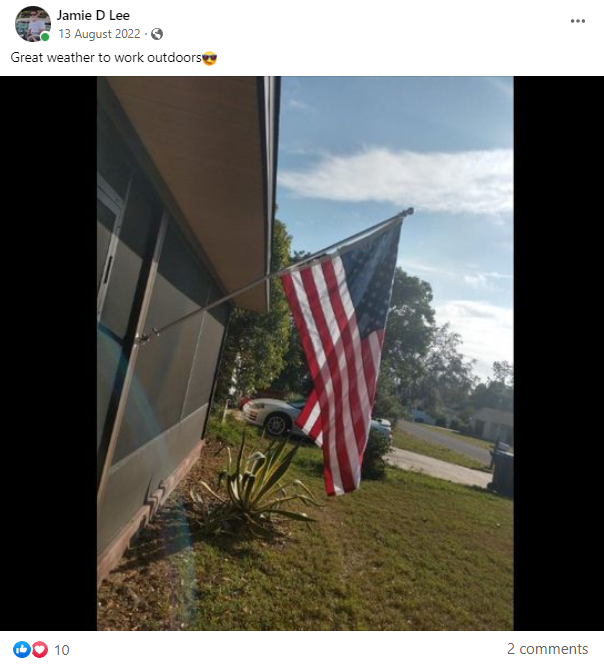

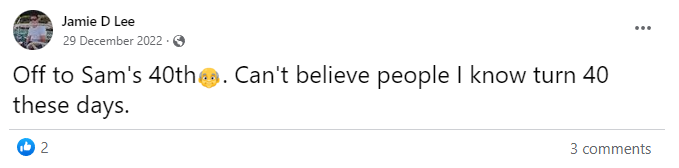

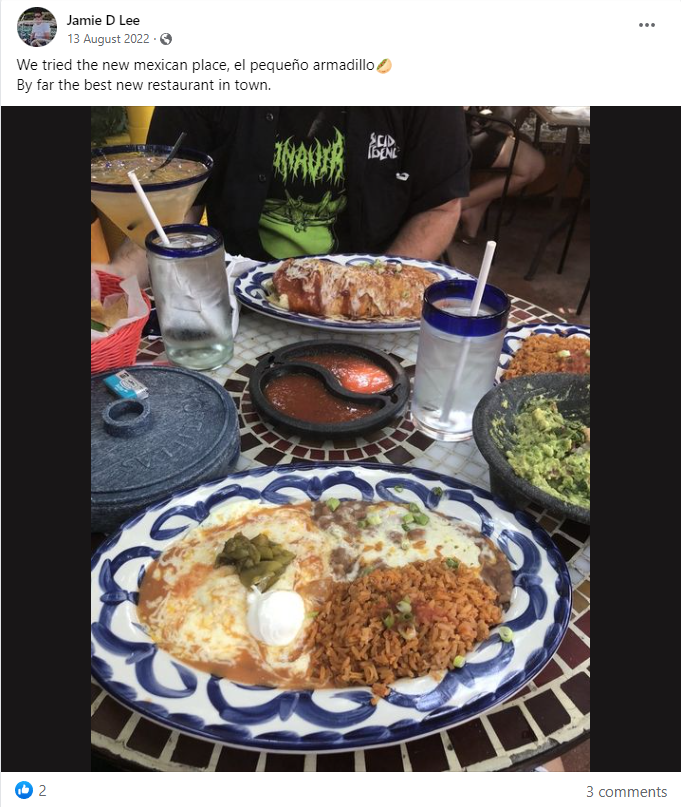


Regular experience condition:


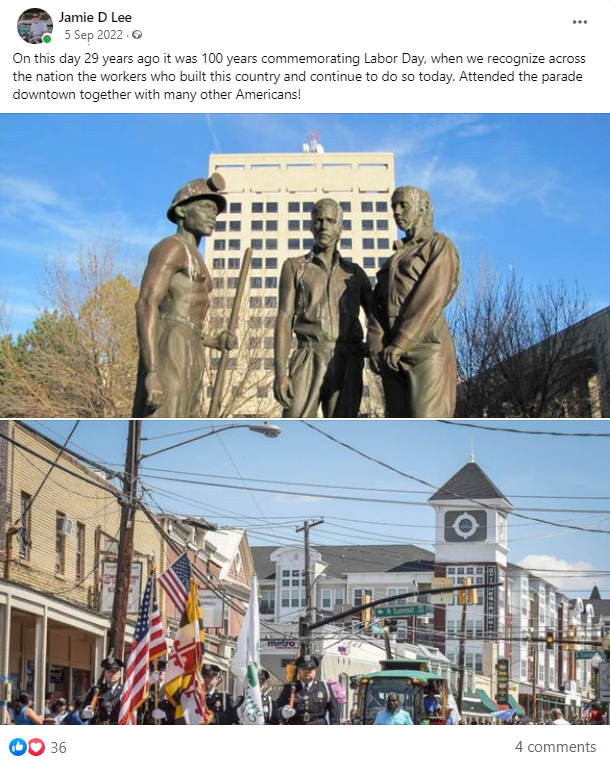


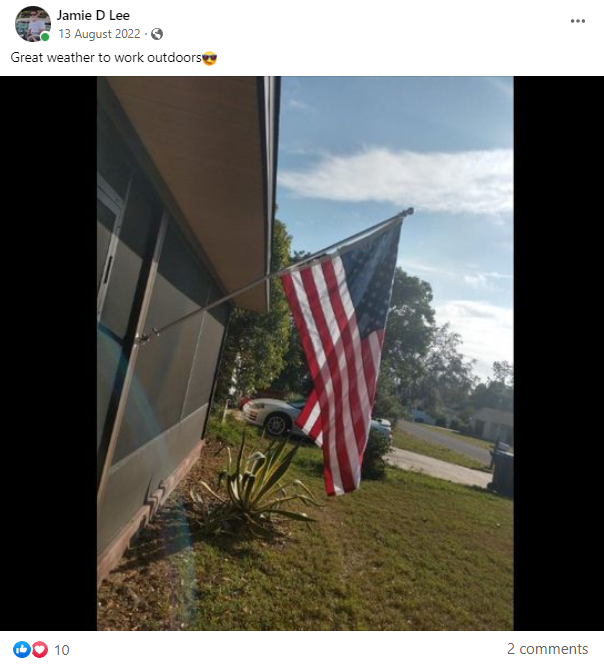


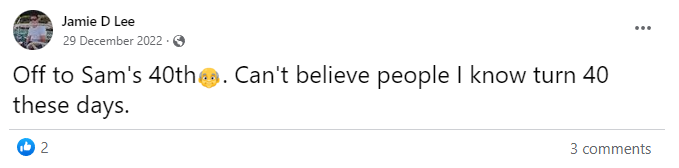

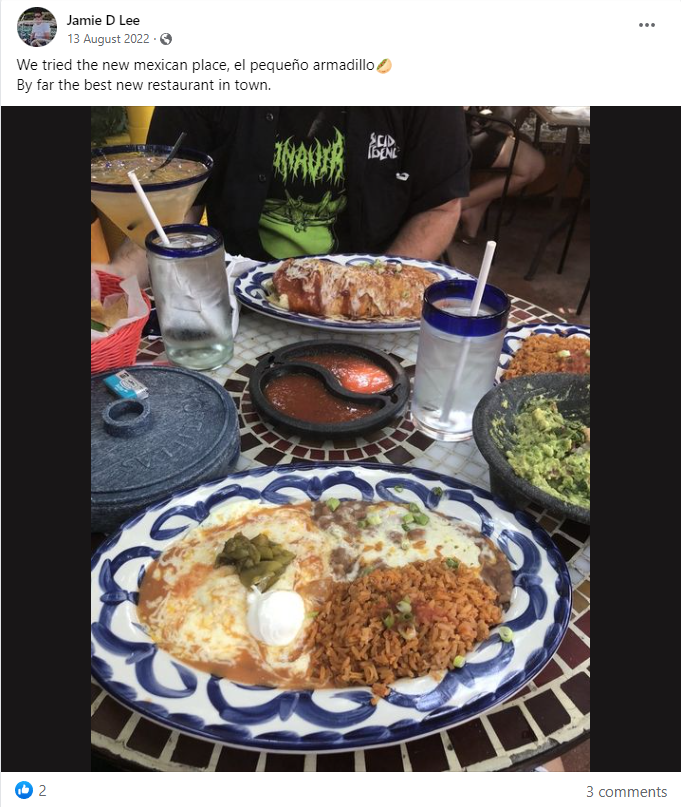


Control condition:


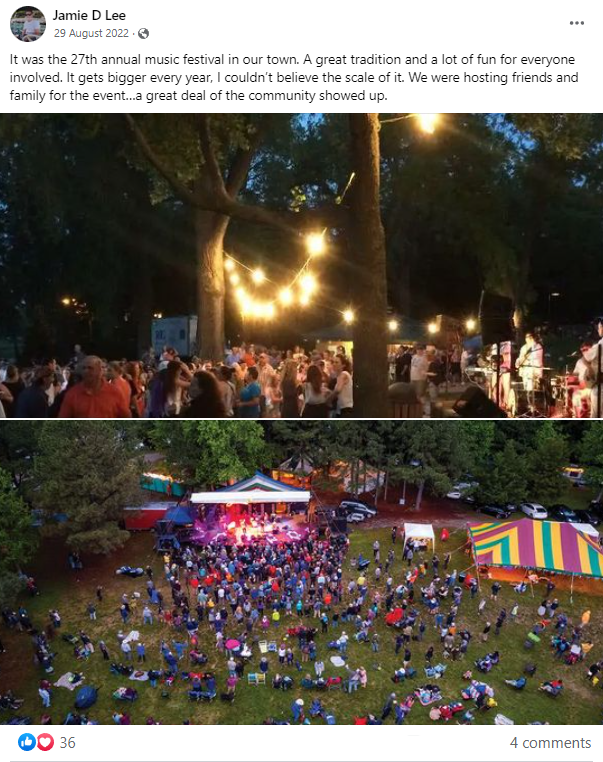


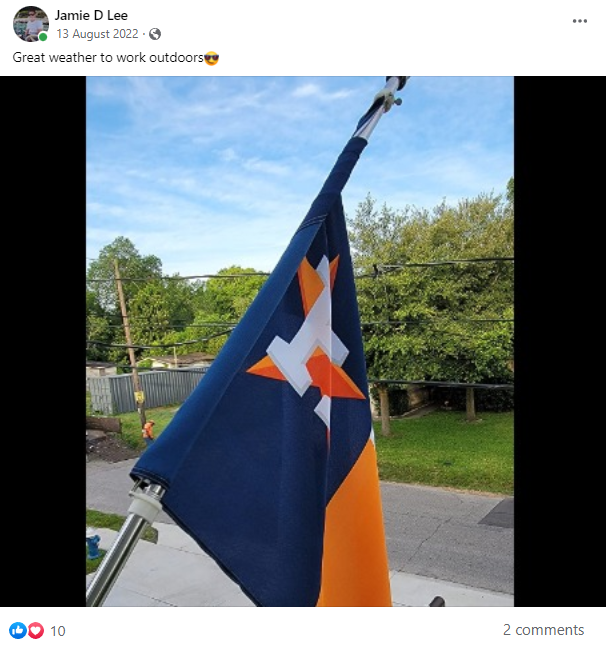


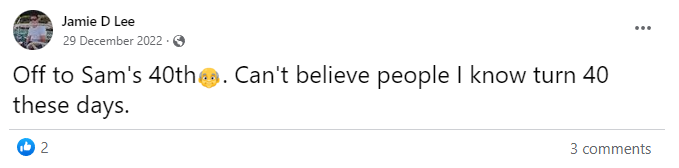

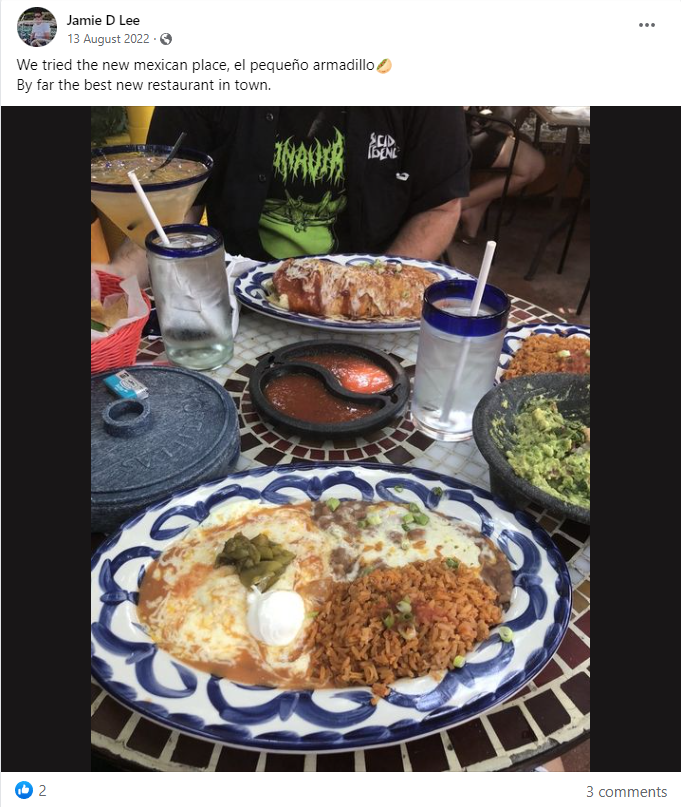


# **Supplementary Information C: Stimuli for Studies 3a-b**

Introduction text

Imagine you are a hiring manager at a building supply store. After reviewing a list of job applicants for an open cashier position, you determine that one candidate is the best qualified. However, the candidate has a special circumstance – he has a criminal record. A background check confirmed that the candidate has a conviction for which he served 22 months in prison.

You have a look at his social media presence and find a Facebook page. Please examine all information on the page to form a more accurate impression of the applicant.”

## **Study 3a (US)**

Dysphoric experience condition:


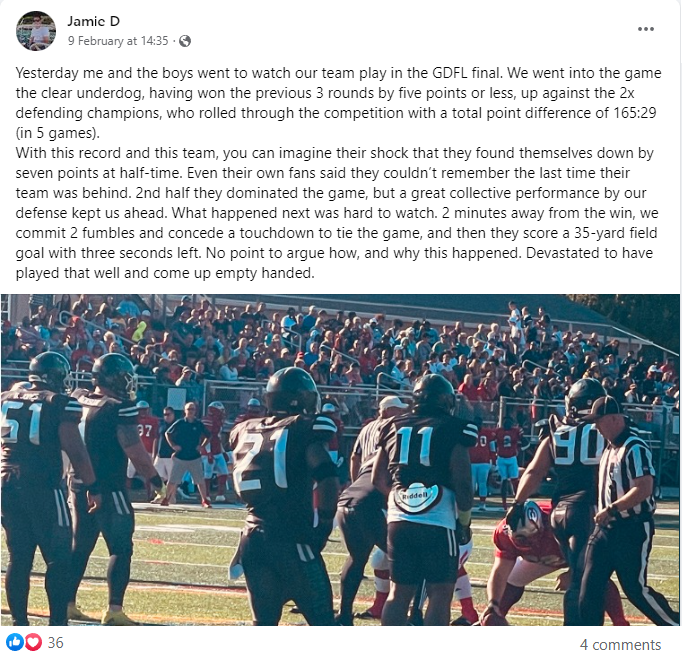


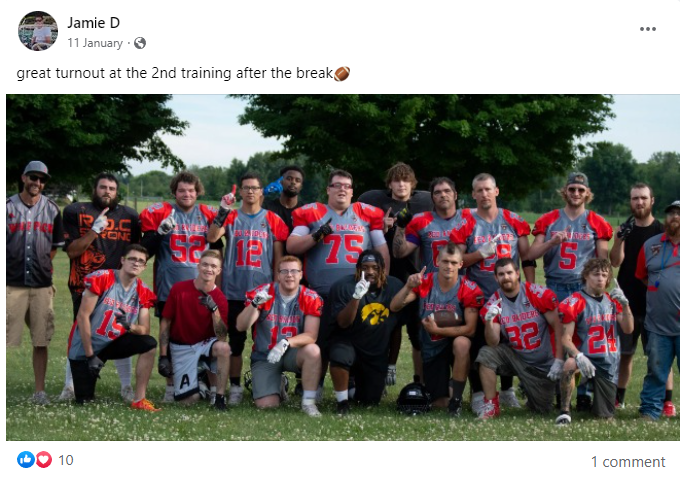


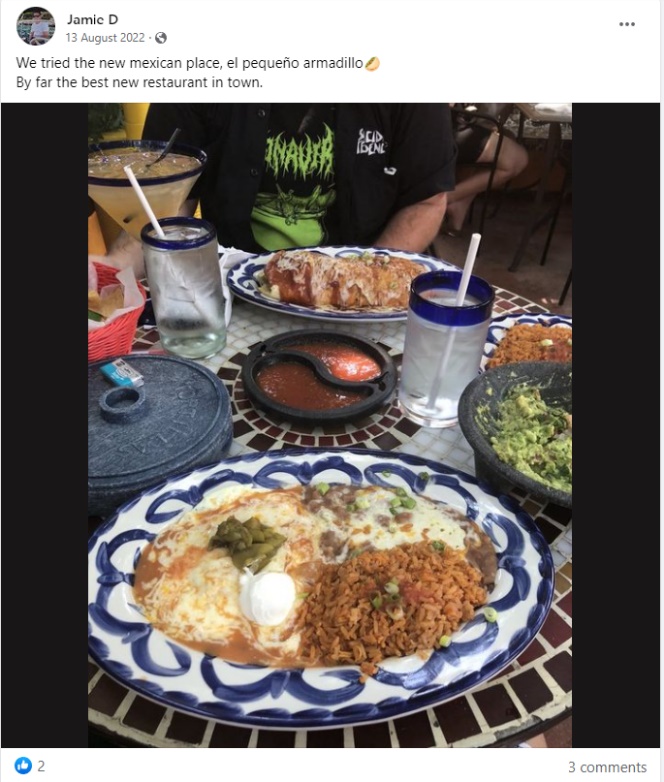

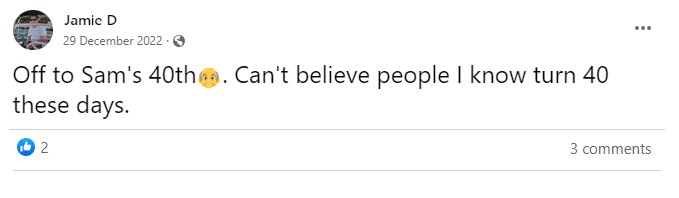


**Regular experience condition:**


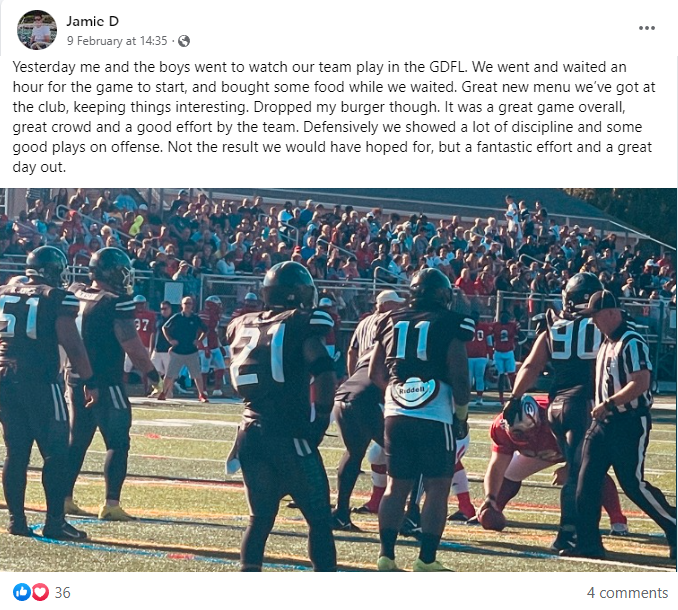


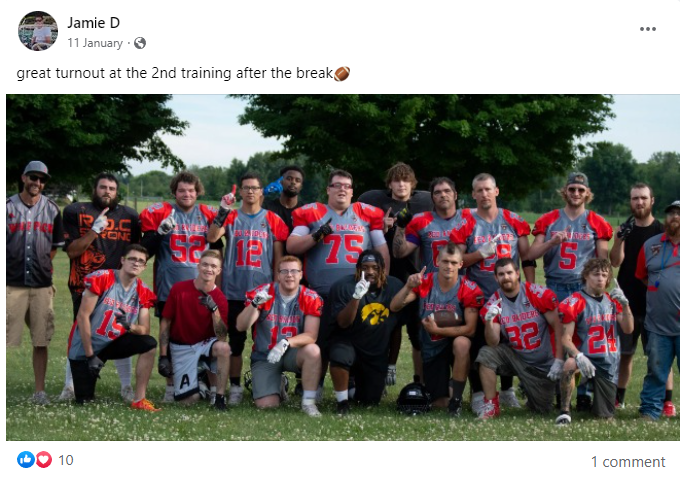


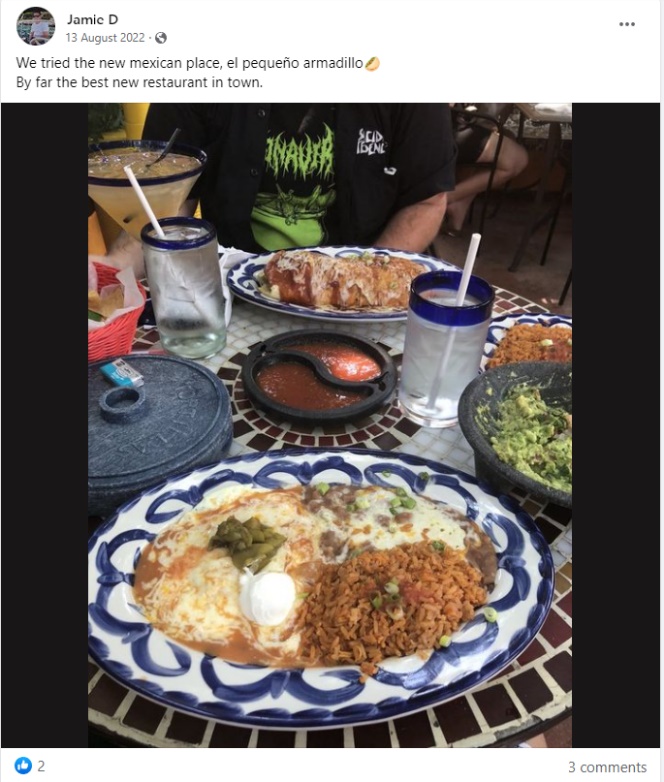

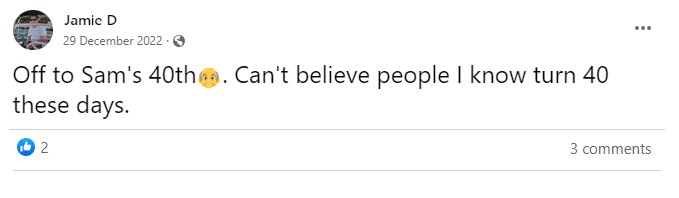


**Control condition:**


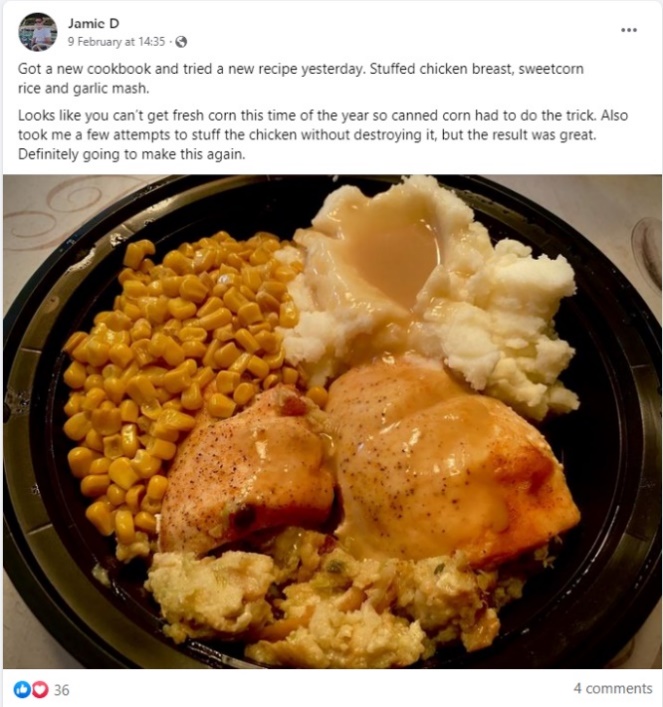

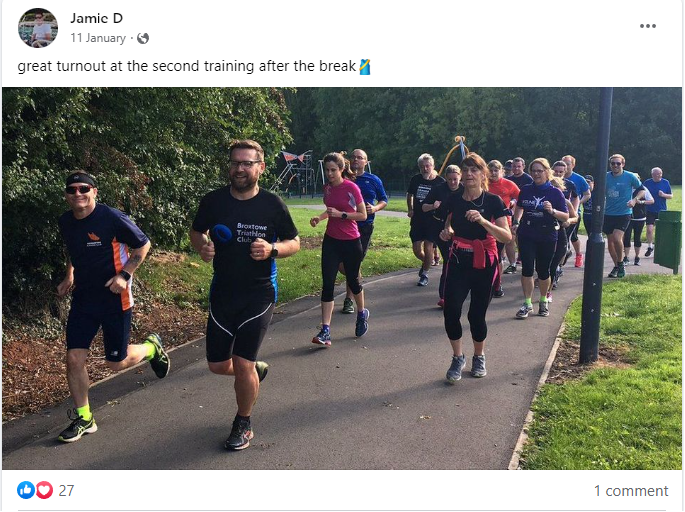


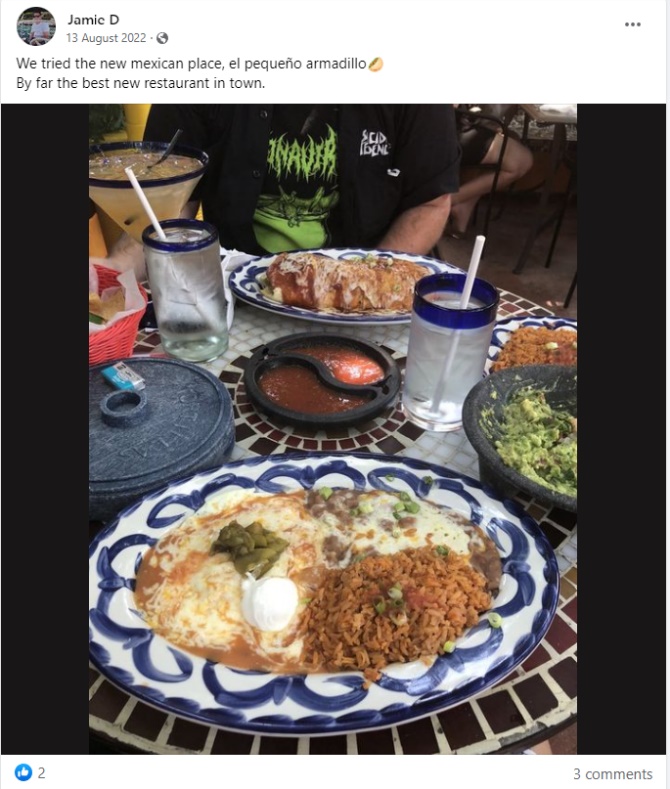

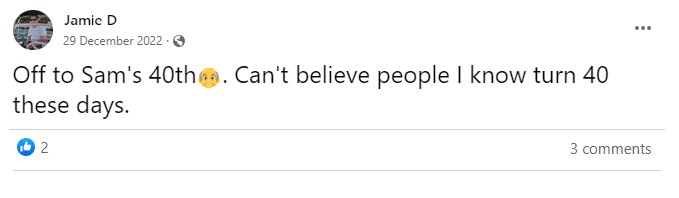


## **Study 3b (UK)**


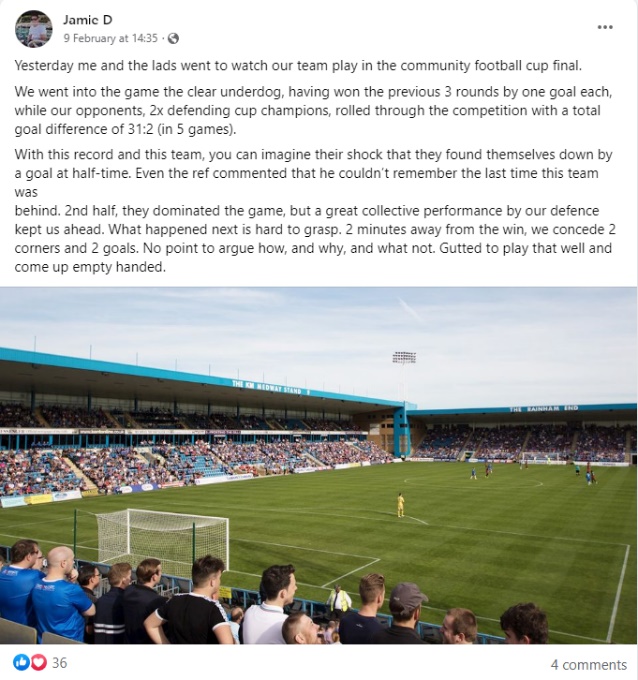
Dysphoric experience condition:


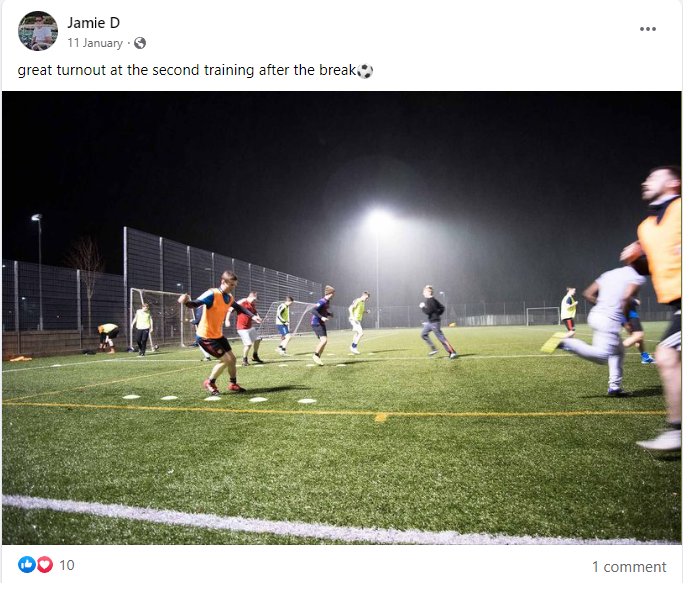


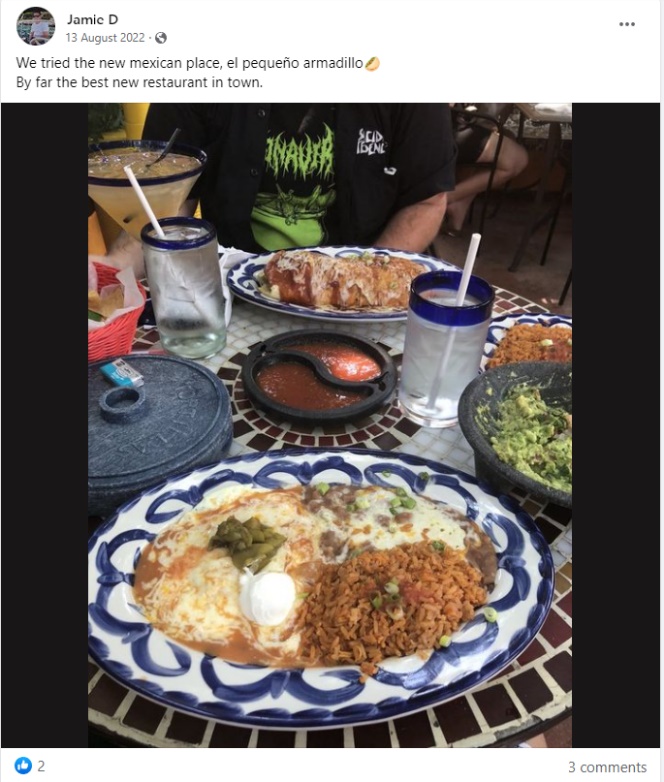

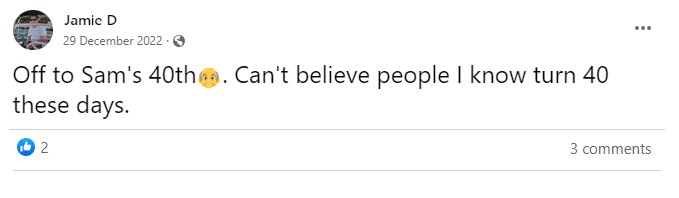


Regular experience condition


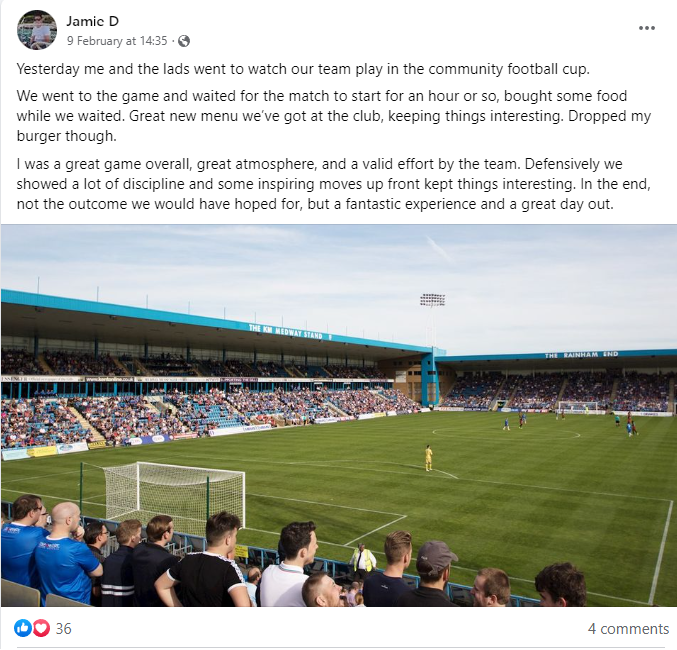

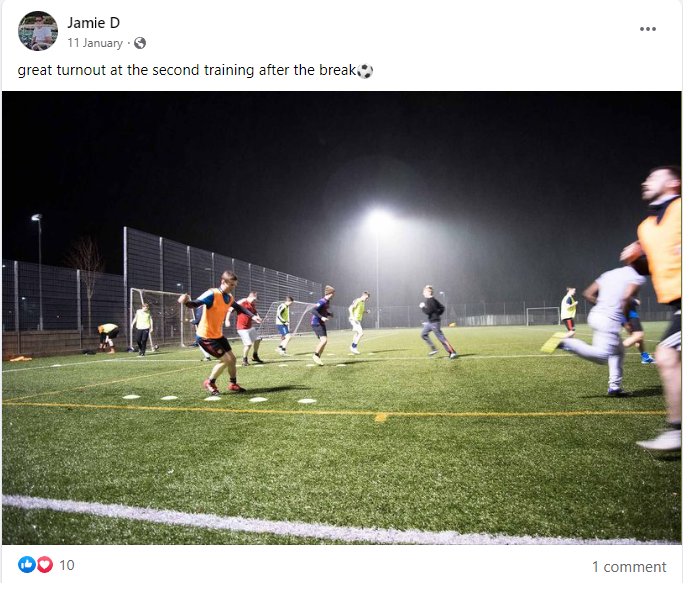

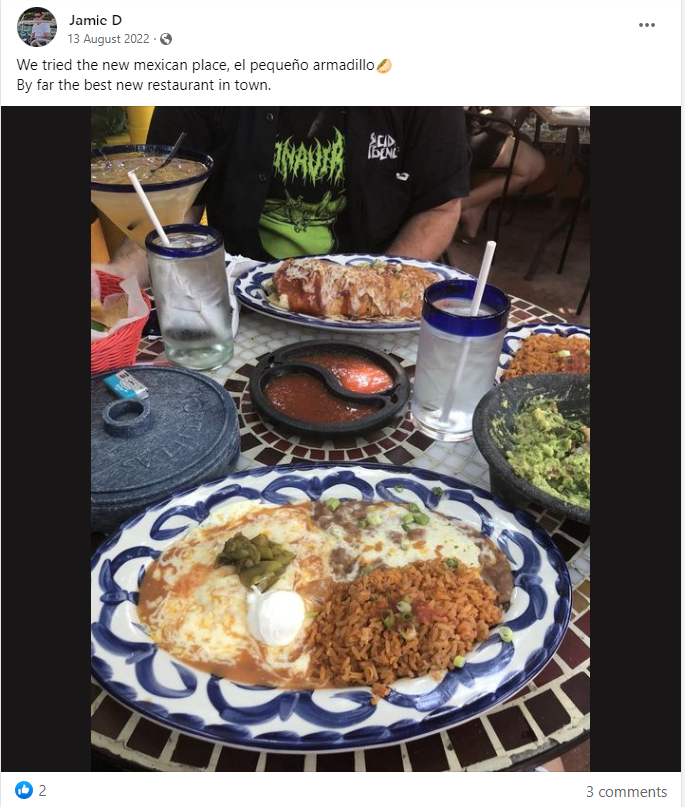

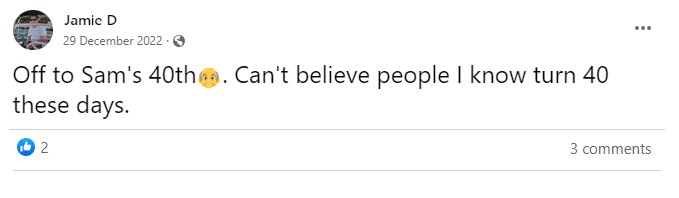


Control condition:


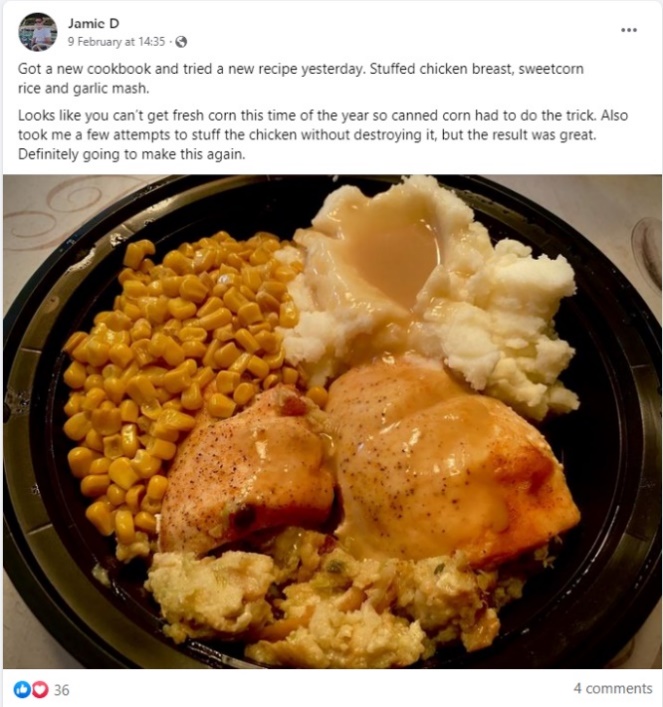

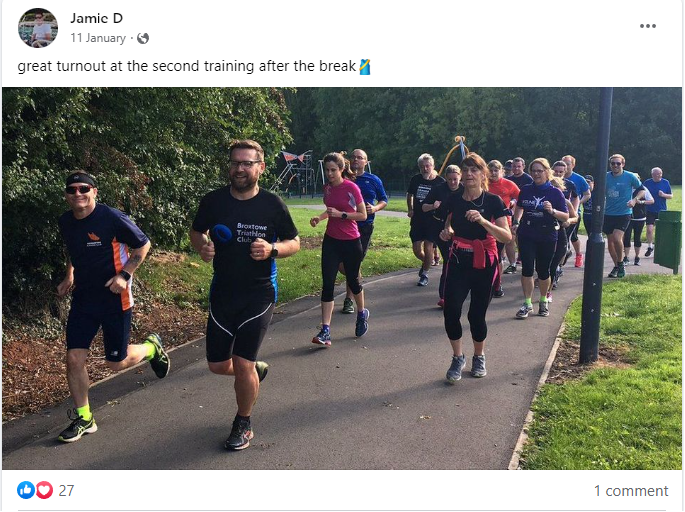


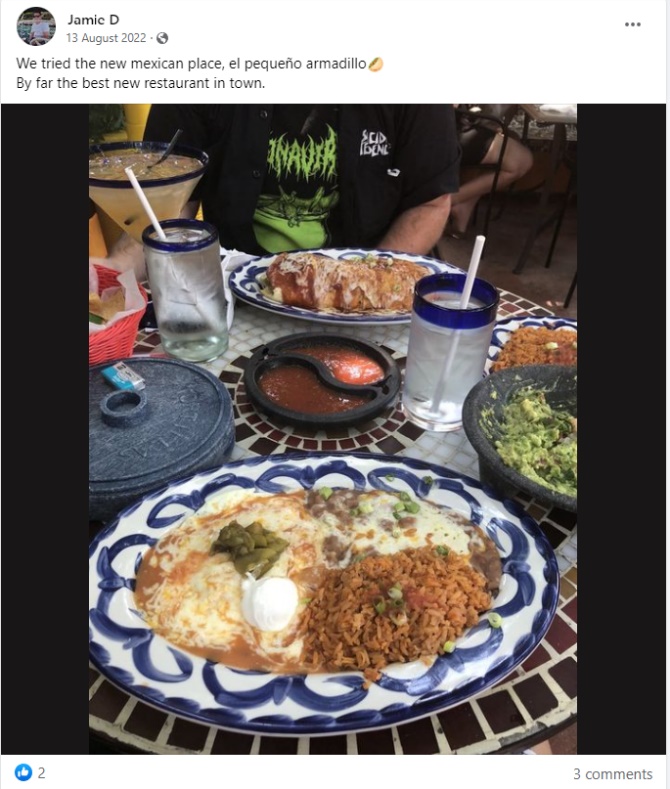

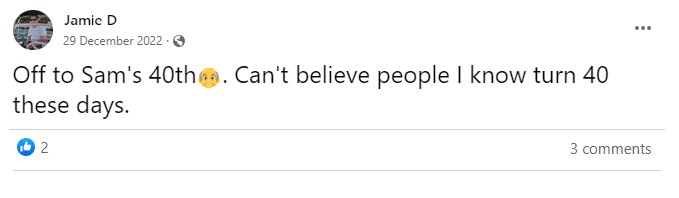


# **Supplementary Information D: Stimuli for Studies 4a-b**

Vignette Introduction text:

Imagine you are a hiring manager at a building supply store. After reviewing a list of job applicants for an open cashier position, you determine that one candidate is the best qualified. However, the candidate has a special circumstance – he has a criminal record. A background check confirmed that the candidate has a conviction for which he served 22 months in prison.

**Study 4a (US)**

Good prison condition:

During his interview, the applicant is asked about other important things to know about him. He talks about the impact of his time in prison and how he spent the majority of his sentence in a medium-security prison. The facility was one of the newer prisons in the US and has been referred to as one of America's best prisons. He explains that the conditions were overall very good, no overcrowding, clean toilets and bathrooms, plenty of opportunities for sports and education. He also mentions that the atmosphere in the prison was very calm, there was not much violence or self-harm among inmates. At the end he emphasises that he's especially motivated to stay on the straight and narrow because of this experience.

Bad prison condition:

During his interview, the applicant is asked about other important things to know about him. He talks about the impact of his time in prison and how he spent the majority of his sentence in a medium-security prison. The facility was one of the older prisons in the US and has been referred to as one of America’s worst prisons. He explains that the conditions were overall very poor, overcrowded, dirty toilets and bathrooms, almost no opportunities for sports and education. He also mentions that the atmosphere in the prison was very tense, that there was a lot of violence and self-harm among inmates. At the end he emphasises that he's especially motivated to stay on the straight and narrow because of this experience.

## **Study 4b (UK)**

Good prison condition:

During his interview, the applicant is asked about other important things to know about him. He talks about the impact of his time in prison and how he spent the majority of his sentence in a medium-security prison. The facility was one of the newer prisons in the UK and has been referred to as one of Britain's best prisons. He explains that the conditions were overall very good, no overcrowding, clean toilets and bathrooms, plenty of opportunities for sports and education. He also mentions that the atmosphere in the prison was very calm, there was not much violence or self-harm among inmates. At the end he emphasises that he's especially motivated to stay on the straight and narrow because of this experience.

Bad prison condition:

During his interview, the applicant is asked about other important things to know about him. He talks about the impact of his time in prison and how he spent the majority of his sentence in a medium-security prison. The facility was one of the older prisons in the UK and has been referred to as one of Britain’s worst prisons. He explains that the conditions were overall very poor, overcrowded, dirty toilets and bathrooms, almost no opportunities for sports and education. He also mentions that the atmosphere in the prison was very tense, that there was a lot of violence and self-harm among inmates. At the end he emphasises that he's especially motivated to stay on the straight and narrow because of this experience.

# **Supplementary Information E: List of Scales for all Studies**

| **Scale** | **Item(s)** | **Response options** |
| --- | --- | --- |
| Willingness to hire (based on Reich et al. (2024)) | To what extent would you be willing to hire this job applicant?” | 1 = totally unwilling, 6 = totally willing |
| Perceived future chances (referred to as ‘willingness to support employee’ in pre-registrations for S1 & S3) | How would you rate the applicant’s chances to stay out of trouble with the law? | 1 = Poor, 2 = Quite poor, 3 = Neither good nor bad, 4 = Good, 5 = Excellent |
| Identity fusion (based on Gomez et al., 2011) | - I am one with the applicant. - I have a deep emotional bond with the applicant. - I am strong because of the applicant. - I make the applicant strong. | 1 = strongly disagree, 7 = strongly agree. |
| Experience Transformativeness (based on Buhrmester et al., 2018) (referred to as ‘imagistic memories’ in pre-registrations for S1 & S3) | Take a moment to think about a past experience of  [S1a/b: of loss and bereavement of family members in your own life. If you have not experienced the loss of a family member, think about a close friend or colleague that you have lost.]  [S2: of a natural disaster that affected you personally, people that are close to you, or people in a group you consider yourself a part of.]  [S3a/b: of bitter sport disappointment and defeat of your favourite team or a team you played on.]  [S4a/b: To start, we want you to take a moment and think about a particularly positive (negative) experience or event in your life that has had a significant impact on you as a person (e.g., an achievement, milestone, good fortune, etc.) (e.g., a set-back, failure, misfortune, etc.]  The following statements are about experiences of this kind. Please rate the extent to which you agree with each statement as it describes you.   - “This experience was…(1) emotionally intense for me, - (2) personally transformative for me - (3) shared deeply with my friends. - (4) I vividly remember this experience   **Note.** For the measure of ‘perceived transformative experiences’ in S4a-b items referred to “he/him/his” | 1 = strongly disagree, 7 = strongly agree. |
| Contact ex-prisoners ( based on Hirschfield & Piquero, 2010). | How many people have you known personally or professionally who have been to prison? | 1 = none, 2 = a few, 3 = several, 4 = many |
| Political ideology | In political matters, people talk of ‘the left’ and ‘the right’.   - How would you place your views on this scale in general? - How would you place your views on this scale when you think about social issues? - How would you place your views on this scale when you think about economic issues? | 0 = left, 1, 2, 3, 4, 5 = center, 6, 7, 8, 9, 10 = right) |
| Open-mindedness/ Implicit Person Theory Measure (Dweck, 1999) | - The kind of person someone is, is something very basic about them and it can’t be changed very much. - People can do things differently, but the important parts of who they are can’t really be changed. - Everyone, no matter who they are, can significantly change their basic characteristics. - As much as I hate to admit it, you can’t teach an old dog new tricks. People can’t really change their deepest attributes. - People can always substantially change the kind of person they are. - Everyone is a certain kind of person, and there is not much that can be done to really change that. - No matter what kind of person someone is, they can always change very much. - All people can change even their most basic qualities.” | (1 = strongly disagree, 6 = strongly agree). |
| Victim status | Have you or a family member ever been a victim of crime? | 1= Yes, 0 = No |
| Age | How old are you? | Dropdown menu (in years) |
| Gender | How do you describe yourself? | male, female, non-binary/third gender, prefer to self-describe, prefer not to say |
| Ethnicity | How do you describe your ethnicity? | Asian, Black, Mixed, North African, White, Prefer not to say |
| Subjective socio-economic status (based on Adler et al., 1994) | Think of the ladder below as representing where people stand in society. At the top of the ladder are the people who are best off – those who have the most money, most education, and the best jobs. At the bottom are the people who are worst off – who have the least money, least education, and the worst jobs or no job. The higher up you are on this ladder, the closer you are to people at the very top and the lower you are, the closer you are to the bottom. Where would you put yourself on the ladder? Please select the number in the box below which corresponds to the rung where you think you stand. | 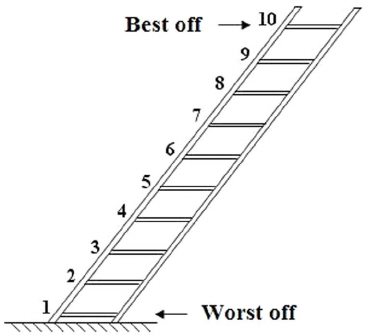 |
| Education | What is the highest level of education you have completed? | - No formal qualifications - School qualifications (e.g., CSE, GCSE, O-Level) - College qualifications (e.g., Apprenticeship, A-Level) - Undergraduate degree or equivalent - Postgraduate degree - Don't know - Prefer not to say |
| Hiring experience | How many hiring decisions do you estimate you have been involved in? | (None, 1-10, 11-20, 21-30, 31-40, 41-50, more than 50) |
| Football fandom | How strongly do you support your favourite football team? | 1 = not at all, 2 = a little, 3 = somewhat, 4 = Big fan, 5 = massive fan |
| Symbolic patriotism (from the 2004 NES, based on Huddy & Khatib, 2007) | How good does it make you feel when you see the American flag flying? | 1 = not at all, 5 = extremely |
| Social desirability (Stoeber, 2001) | - I sometimes litter. - I always admit my mistakes openly and face the potential negative consequences. - In traffic I am always polite and considerate of others. - I always accept others' opinions, even when they don't agree with my own. - I take out my bad moods on others now and then. - There has been an occasion when I took advantage of someone else. - In conversations I always listen attentively and let others finish their sentences. - I never hesitate to help someone in case of emergency. - When I have made a promise, I keep it--no ifs, ands or buts. - I occasionally speak badly of others behind their back. - I would never live off other people. - I always stay friendly and courteous with other people, even when I am stressed out. - During arguments I always stay objective and matter-of-fact. - I always stay friendly and courteous with other people, even when I am stressed out. - There has been at least one occasion when I failed to return an item that I borrowed. - I always eat a healthy diet. - Sometimes I only help because I expect something in return. | 1 = True, 0 = False |
